# Circulating proteins and risk of pancreatic cancer: a case-subcohort study among Chinese adults

**DOI:** 10.1093/ije/dyab274

**Published:** 2022-01-22

**Authors:** Christiana Kartsonaki, Yuanjie Pang, Iona Millwood, Ling Yang, Yu Guo, Robin Walters, Jun Lv, Michael Hill, Canqing Yu, Yiping Chen, Xiaofang Chen, Eric O’Neill, Junshi Chen, Ruth C Travis, Robert Clarke, Liming Li, Zhengming Chen, Michael V Holmes

**Affiliations:** Clinical Trial Service Unit & Epidemiological Studies Unit (CTSU), Nuffield Department of Population Health, University of Oxford, Oxford, UK; Medical Research Council Population Health Research Unit (MRC PHRU), Nuffield Department of Population Health, University of Oxford, Oxford, UK; Department of Epidemiology and Biostatistics, School of Public Health, Peking University, Beijing, China; Clinical Trial Service Unit & Epidemiological Studies Unit (CTSU), Nuffield Department of Population Health, University of Oxford, Oxford, UK; Medical Research Council Population Health Research Unit (MRC PHRU), Nuffield Department of Population Health, University of Oxford, Oxford, UK; Clinical Trial Service Unit & Epidemiological Studies Unit (CTSU), Nuffield Department of Population Health, University of Oxford, Oxford, UK; Medical Research Council Population Health Research Unit (MRC PHRU), Nuffield Department of Population Health, University of Oxford, Oxford, UK; CKB Project Department, Chinese Academy of Medical Sciences, Beijing, China; Clinical Trial Service Unit & Epidemiological Studies Unit (CTSU), Nuffield Department of Population Health, University of Oxford, Oxford, UK; Medical Research Council Population Health Research Unit (MRC PHRU), Nuffield Department of Population Health, University of Oxford, Oxford, UK; Department of Epidemiology and Biostatistics, School of Public Health, Peking University, Beijing, China; Clinical Trial Service Unit & Epidemiological Studies Unit (CTSU), Nuffield Department of Population Health, University of Oxford, Oxford, UK; Department of Epidemiology and Biostatistics, School of Public Health, Peking University, Beijing, China; Clinical Trial Service Unit & Epidemiological Studies Unit (CTSU), Nuffield Department of Population Health, University of Oxford, Oxford, UK; Medical Research Council Population Health Research Unit (MRC PHRU), Nuffield Department of Population Health, University of Oxford, Oxford, UK; NCDs Prevention and Control Department, Pengzhou CDC, Pengzhou City, Sichuan Province, China; Department of Oncology, University of Oxford, Oxford, UK; NHD Key Laboratory of Food Safety Risk Assessment, China National Center for Food Safety Risk Assessment, Beijing, China; Cancer Epidemiology Unit (CEU), Nuffield Department of Population Health, University of Oxford, Oxford, UK; Clinical Trial Service Unit & Epidemiological Studies Unit (CTSU), Nuffield Department of Population Health, University of Oxford, Oxford, UK; Department of Epidemiology and Biostatistics, School of Public Health, Peking University, Beijing, China; Clinical Trial Service Unit & Epidemiological Studies Unit (CTSU), Nuffield Department of Population Health, University of Oxford, Oxford, UK; Medical Research Council Population Health Research Unit (MRC PHRU), Nuffield Department of Population Health, University of Oxford, Oxford, UK; Clinical Trial Service Unit & Epidemiological Studies Unit (CTSU), Nuffield Department of Population Health, University of Oxford, Oxford, UK; Medical Research Council Population Health Research Unit (MRC PHRU), Nuffield Department of Population Health, University of Oxford, Oxford, UK; National Institute for Health Research Oxford Biomedical Research Centre, John Radcliffe University Hospital, Oxford, UK

**Keywords:** Pancreatic cancer, proteomics, biomarkers, risk prediction, early diagnosis

## Abstract

**Background:**

Pancreatic cancer has a very poor prognosis. Biomarkers that may help predict or diagnose pancreatic cancer may lead to earlier diagnosis and improved survival.

**Methods:**

The prospective China Kadoorie Biobank (CKB) recruited 512* *891 adults aged 30–79 years during 2004–08, recording 702 incident cases of pancreatic cancer during 9 years of follow-up. We conducted a case-subcohort study measuring 92 proteins in 610 cases and a subcohort of 623 individuals, using the OLINK immuno-oncology panel in stored baseline plasma samples. Cox regression with the Prentice pseudo-partial likelihood was used to estimate adjusted hazard ratios (HRs) for risk of pancreatic cancer by protein levels.

**Results:**

Among 1233 individuals (including 610 cases), several chemokines, interleukins, growth factors and membrane proteins were associated with risk of pancreatic cancer, with adjusted HRs per 1 standard deviation (SD) of 0.86 to 1.86, including monocyte chemotactic protein 3 (MCP3/CCL7) {1.29 [95% CI (confidence interval) (1.10, 1.51)]}, angiopoietin-2 (ANGPT2) [1.27 (1.10, 1.48)], interleukin-18 (IL18) [1.24 (1.07, 1.43)] and interleukin-6 (IL6) [1.21 (1.06, 1.38)]. Associations between some proteins [e.g. matrix metalloproteinase-7 (MMP7), hepatocyte growth factor (HGF) and tumour necrosis factor receptor superfamily member 9 [TNFRSF9)] and risk of pancreatic cancer were time-varying, with higher levels associated with higher short-term risk. Within the first year, the discriminatory ability of a model with known risk factors (age, age squared, sex, region, smoking, alcohol, education, diabetes and family history of cancer) was increased when several proteins were incorporated (weighted C-statistic changed from 0.85 to 0.99; *P* for difference = 4.5 × 10^–5^), although only a small increase in discrimination (0.77 to 0.79, *P* = 0.04) was achieved for long-term risk.

**Conclusions:**

Several plasma proteins were associated with subsequent diagnosis of pancreatic cancer. The potential clinical utility of these biomarkers warrants further investigation.

Key Messages

- Pancreatic cancer is difficult to diagnose and it is difficult to predict the risk of an individual developing it. Its prognosis is very poor and it is often diagnosed late. A few biomarkers exist but their utility is limited.
- In this case-subcohort study including >600 pancreatic cancer cases, we identified several circulating proteins which were associated with short- and long-term risk of pancreatic cancer.
- Measurement of such proteins in blood samples may help identify individuals at high risk of pancreatic cancer or may aid in its diagnosis.

## Introduction

Pancreatic cancer has a 5-year survival of 5–10% and a median survival of 4–6 months.^1^ Most patients are diagnosed at a late stage when surgical resection is not possible and treatment options are limited.^2^ This is mainly due to patients developing symptoms late in the course of disease, symptoms being non-specific,^3^ lack of effective screening tools, and challenges in diagnosis,^4^ which is currently based mainly on computed tomography (CT) and/or magnetic resonance imaging (MRI) with magnetic resonance cholangiopancreatography (MRCP), or biopsy or fine-needle aspiration using endoscopic ultrasound (EUS).^5^ Non-invasive tests of predictive utility therefore have the potential to transform patient care.

The aetiology of pancreatic cancer remains poorly understood, although several risk factors have been identified, such as diabetes, chronic pancreatitis, smoking, family history of certain cancers and some germline mutations, adiposity, alcohol consumption, gallstones, dietary factors and some chronic infections.^1^^,^^6–10^ Inflammation plays an important role in pancreatic carcinogenesis.^11^^,^^12^ Precursor lesions exist but many are undetectable by imaging.^13^ However, pancreatic intraepithelial neoplasia (PanIN) lesions may secrete factors that modify their microenvironment.^14^

Although some risk factors, signs and symptoms can help identify individuals at high risk, predicting risk of pancreatic cancer is challenging. A few biomarkers have been identified, carbohydrate antigen 19–9 (CA 19–9) being the most well established, but their discriminatory ability is limited and they are not recommended for screening asymptomatic individuals.^15^^,^^16^ Other tumour markers and proteins have been studied but they have not been shown to substantially improve on the sensitivity and specificity of CA 19–9 alone.^17^ A compendium of secreted proteins overexpressed in pancreatic cancer has been published^18^ and such blood-based biomarkers may have a role in predicting or diagnosing the disease. In this case-subcohort study within the China Kadoorie Biobank (CKB), we aimed to examine the prospective associations of >90 protein biomarkers with development of pancreatic cancer and to assess the extent to which they could help predict risk of a future diagnosis.

## Methods

### Study population

The CKB is a prospective cohort study of 512* *891 Chinese adults aged 30–79 years who were recruited from 10 geographically defined localities (five urban and five rural) in China during 2004–08.^19^ Ethics approval from the Oxford University Tropical Research Ethics Committee, the Chinese Centre for Disease Control and Prevention (CDC) Ethical Review Committee and the local CDC of each study area was obtained, and all participants provided written informed consent.

### Case-subcohort study of pancreatic cancer

We designed a case-subcohort study to examine the associations of proteins with risk of pancreatic cancer. All 700 pancreatic cancer cases (ICD-10 C25) that accumulated until 1 January 2016 and had an available plasma sample were included. A subcohort of 700 participants was sampled using simple random sampling from a randomly selected subset of the baseline cohort.

### Measurement of protein biomarkers

The OLINK immuno-oncology panel of 92 proteins was used, which uses proximity extension assay (PEA) technology to obtain normalized protein expression (NPX) values for the 92 proteins. These proteins are involved in tumour immunity, chemotaxis, vascular and tissue remodelling, apoptosis and autophagy (Supplementary Table S1, available as Supplementary data at *IJE* online). The grouping of the 92 proteins according to their main protein class and function is shown in Supplementary Table S2, available as Supplementary data at *IJE* online. The limit of detection (LOD) for each protein is given in Supplementary Table S3, available as Supplementary data at *IJE* online.

### Statistical analysis

In total, plasma samples of 1397 participants were assayed. Participants with a history of cancer at baseline (*n *= 21) were excluded from the main analyses. Moreover, 145 samples with either a quality control warning or precipitation (partly overlapping with those with prior cancer) were also excluded, leaving 1233 individuals (610 cases and 623 subcohort members) for the main analyses.

The associations between proteins and risk of pancreatic cancer were assessed using Cox proportional hazards models, using the Prentice pseudo-partial likelihood.^20^ Models in the main analysis were stratified by region and adjusted for age, age squared, sex, smoking, alcohol drinking, educational attainment, diabetes, and time since last meal, and time in study was used as the time scale.

Proteins were standardized (i.e. values of each marker were divided by its standard deviation) in analyses where they were treated as continuous variables. For each marker, adjusted hazard ratios (HRs) and 95% confidence intervals (CIs) per 1 standard deviation (SD) increase in protein expression were estimated. The shape of the associations was assessed by splitting protein values into groups at their quartiles and additionally by using splines (penalized splines with four degrees of freedom). The plausibility of the proportional hazards assumption was assessed using plots of scaled Schoenfeld residuals and the associated chi square tests.^21^^,^^22^ We explored time dependence of associations by examining whether associations varied by the number of years between blood collection and time at risk (four groups: <1, 1 to <2, 2 to <5, ≥5 years) and by including an interaction with log(time + 0.01).

We interpreted *P*-values <0.05 as providing some evidence of an association. In addition, transformed *P*-values (− log *P*) were plotted against their expected values based on the Rényi decomposition,^23^ and adjusted *P*-values were calculated using the false discovery rate correction of Benjamini and Hochberg^24^ to aid interpretation.

Multivariable models with several proteins were fitted using the approach of Cox and Battey.^25^ Discrimination of risk prediction models was assessed using a weighted C-index.^26^ Further details are given in Supplementary Methods, available as Supplementary data at *IJE* online.

## Results

### Characteristics of individuals in the case-subcohort study

Of the 1233 participants included in the main analysis, the mean age at study baseline of pancreatic cancer cases was higher than that of subcohort participants [60.3 (SD 9.0) vs 52.1 (10.5)]. There was a lower proportion of females among cases than in the subcohort (50.6% vs 60.9%), but similar proportions of living in urban regions and similar levels of adiposity. Moreover, cases were more likely to have had regularly smoked, regularly consumed alcohol, to have rated their health as poor and to have diabetes at baseline (13.6% vs 6.3%). Among pancreatic cancer cases, the median time from study entry to diagnosis was 5.3 years [interquartile range (IQR) 4.3, range 0.05 to 11.1] and mean age at diagnosis was 66.0 (SD 8.9) (Table 1).

**Table 1:** Baseline characteristics of pancreatic cancer cases and subcohort participants

	Cases (*n* = 610)	Subcohort (*n* = 623)
Mean age (SD), years	60.3 (9.0)	52.1 (10.5)
Female, %	50.6	60.9
Living in urban area, %	48.7	50.1
Middle school education or above, %	36.9	52.2
Household income ≥35* *000 yuan/year, %	20.2	18.0
Ever-regular smoker, %		
Male	80.8	73.7
Female	7.1	4.2
Ever-regular alcohol drinking, %		
Male	46.7	34.2
Female	3.9	2.6
Mean MET (SD), hours/day	19.1 (14.0)	20.4 (14.5)
Mean BMI (SD), kg/m^2^	23.8 (3.5)	23.8 (3.5)
Mean body fat percentage (SD)	26.9 (9.1)	28.5 (8.5)
Mean SBP (SD), mmHg	137.2 (21.2)	131.3 (21.8)
Diabetes,^a^ %	13.6	6.3
Family history of diabetes, %	5.9	8.3
Family history of cancer, %	17.2	18.3
Poor self-rated health, %	15.1	8.5

BMI, body mass index; SBP, systolic blood pressure; MET, metabolic equivalent of task.

a Self-reported or screen-detected.

### Distribution of proteins

Most proteins were approximately normally distributed. The distributions of some markers were skewed, and for a few markers a high proportion of individuals had a value below the LOD (Supplementary Figure S1, available as Supplementary data at *IJE* online). Correlations between markers were low to moderate (Supplementary Figure S2, available as Supplementary data at *IJE* online).

### Associations with risk of pancreatic cancer

**Figure 1:**
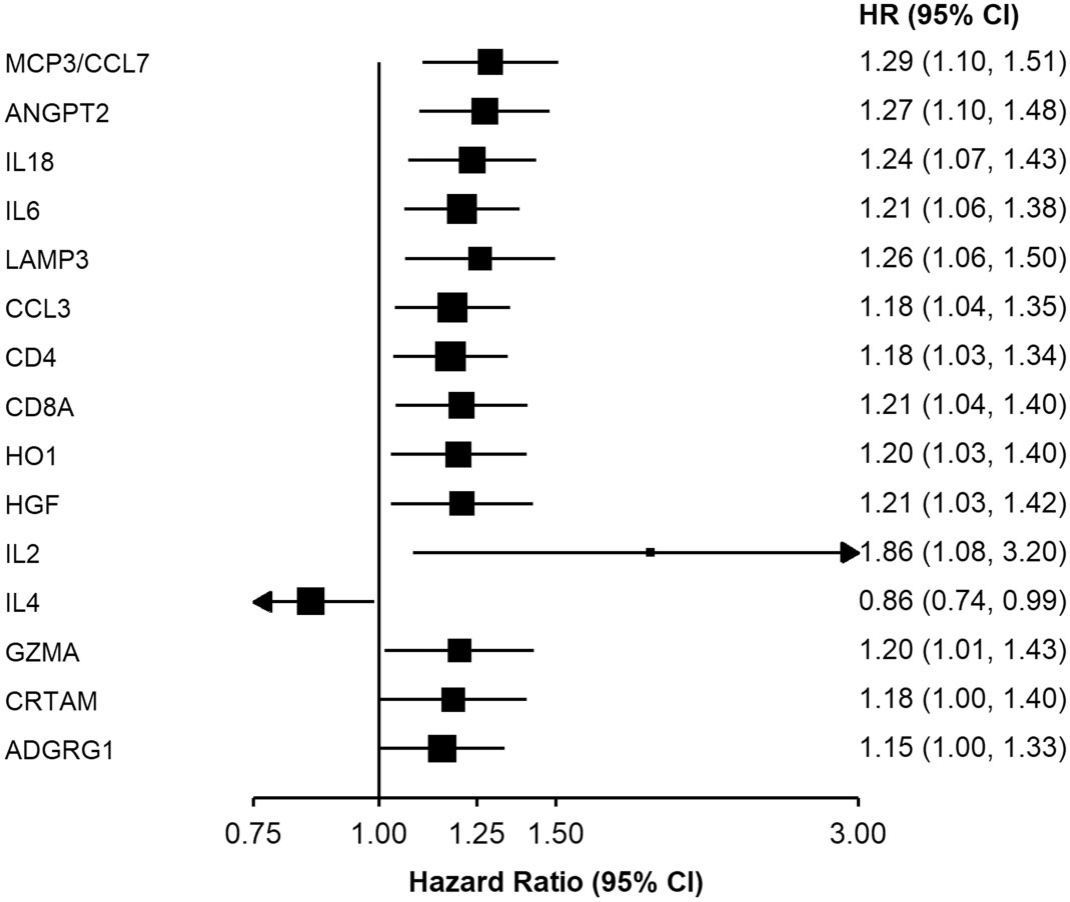
Adjusted hazard ratios for pancreatic cancer per standard deviation increase in normalized protein expression for selected proteins. Model was adjusted for age, age squared, sex, smoking status, alcohol drinking, education, diabetes, and time since last meal, and stratified by region. Time in study was used as the time scale. The boxes are HRs and the horizontal lines are 95% CIs. The area of the box is inversely proportional to the variance of the logHR. MCP3/CCL7: monocyte chemotactic protein 3; ANGPT2: angiopoietin-2; IL18: interleukin-18; IL6: interleukin-6; LAMP3: lysosome-associated membrane glycoprotein 3; CCL3: C-C motif chemokine 3; CD4: T cell surface glycoprotein; CD8A: T cell surface glycoprotein CD8 alpha chain; HO1: haeme oxygenase 1; HGF: hepatocyte growth factor; IL2: interleukin-2; IL4: interleukin-4; GZMA: granzyme A; CRTAM: cytotoxic and regulatory T cell molecule; ADGRG1: adhesion G-protein coupled receptor G1

**Figure 2:**
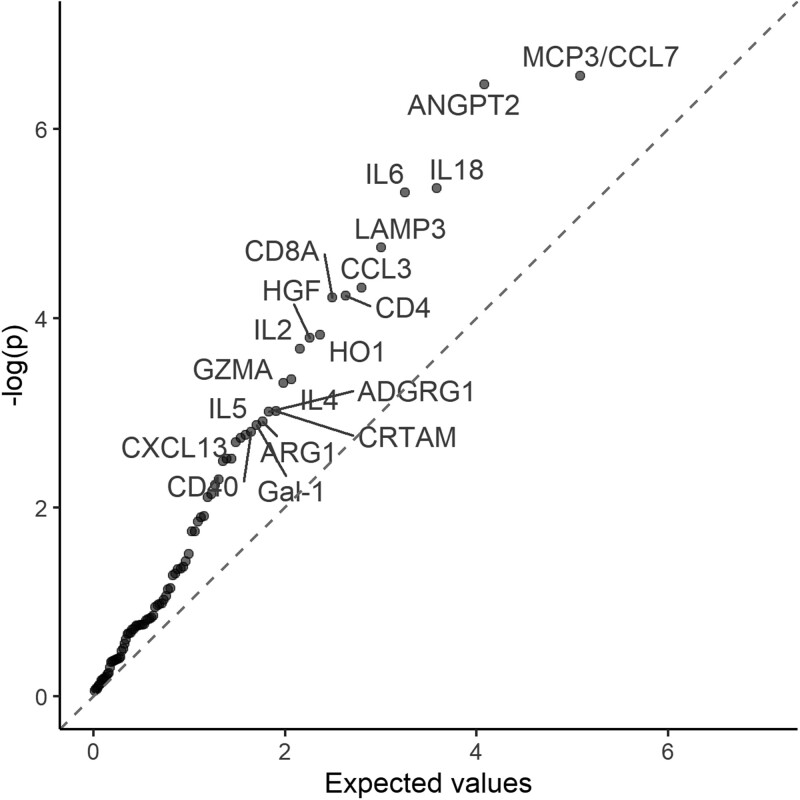
Rényi plot of transformed *P*-values against their expected values. Models were adjusted for age, age squared, sex, smoking status, alcohol drinking, education, diabetes, and time since last meal, and stratified by region. Time in study was used as the time scale. The dashed line is a line of slope 1. Protein names are given in Supplementary table S1.

Several proteins were found to be associated with risk of pancreatic cancer (Figure 1 and Supplementary Table S4, available as Supplementary data at *IJE* online). For most of these proteins, higher levels were associated with a higher risk of pancreatic cancer, including monocyte chemotactic protein 3 (MCP3/CCL7), angiopoietin-2 (ANGPT2), interleukin-18 (IL18), interleukin-6 (IL6), lysosome-associated membrane glycoprotein 3 (LAMP3), C-C motif chemokine 3 (CCL3), T cell surface glycoprotein CD4 (CD4), T cell surface glycoprotein CD8 alpha chain (CD8A), haeme oxygenase 1 (HO1), hepatocyte growth factor (HGF), interleukin-2 (IL2), granzyme A (GZMA), cytotoxic and regulatory T cell molecule (CRTAM) and adhesion G-protein coupled receptor G1 (ADGRG1), with adjusted HRs per standard deviation (SD) increment in NPX ranging from 1.15 [95% CI (1.00, 1.33)] for ADGRG1 to 1.86 (1.08, 3.20) for IL2. Interleukin-4 (IL4) was inversely associated with pancreatic cancer risk. The plot of transformed *P*-values against their expected values supports their associations with pancreatic cancer risk (Figure 2).

Associations were similar when models were only adjusted for age and sex and stratified by region (Supplementary Figure S3 and Supplementary Table S5, available as Supplementary data at *IJE* online). When dichotomizing the 13 proteins for which ≥500 individuals had values below LOD into less than and greater than or equal to LOD, tumour necrosis factor (TNF) was associated with a higher risk of pancreatic cancer [HR = 2.36, 95% CI (1.31, 4.25); *P* = 0.0043]. The findings were otherwise in concordance with the analysis treating them as continuous variables, although associations tended to be less precisely estimated (Supplementary Figure S4, available as Supplementary data at *IJE* online). Supplementary Figure S5, available as Supplementary data at *IJE* online, shows associations of proteins per SD higher NPX by protein class. Among the chemokines, MCP3 was most strongly associated with pancreatic cancer risk. C-C motif chemokines showed a trend towards a positive association, whereas most C-X-C motif chemokines showed no evidence of association. Among interleukins, IL2, IL6 and IL18 were positively associated and IL4 was inversely associated with risk. A few members of the TNF(R) superfamily, two growth factors (ANGPT2 and HGF) and two enzymes (GZMA and HO1) were positively associated with risk. Of the membrane proteins, CD4 and CD8A were positively associated with risk.

**Figure 3:**
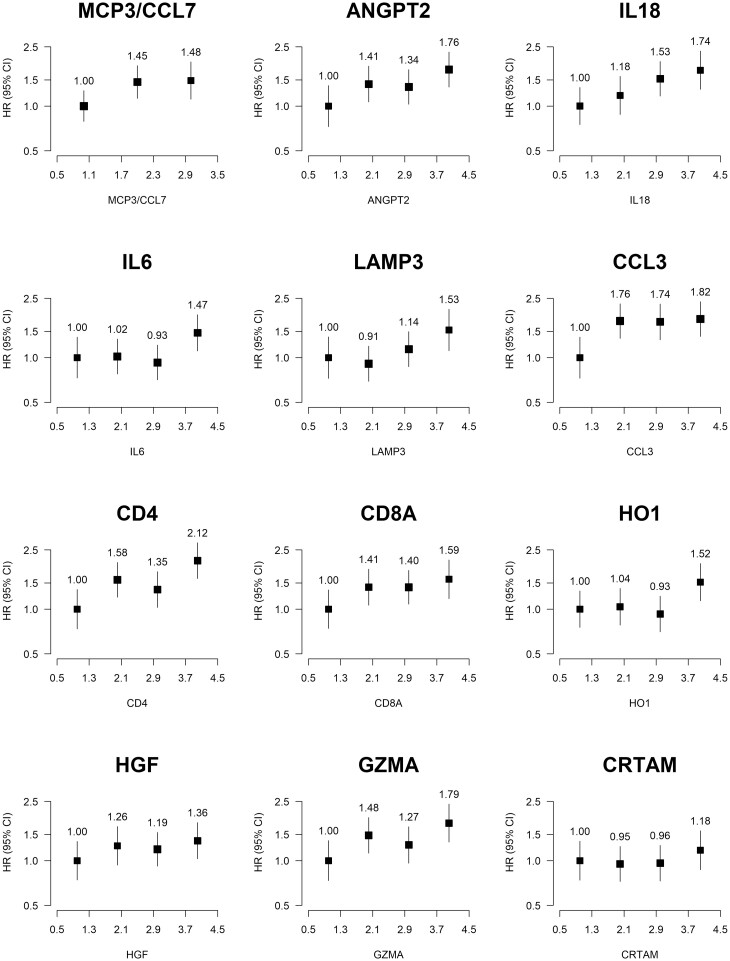
Adjusted hazard ratios for pancreatic cancer associated with selected proteins by normalized protein expression split at quartiles. Proteins were split at tertiles when quartiles were not unique. Models were adjusted for age, age squared, sex, smoking status, alcohol drinking, education, diabetes, and time since last meal, and stratified by region. Time in study was used as the time scale. The boxes are HRs and the vertical lines 95% CIs. The area of the box is inversely proportional to the variance of the logHR. The number above the box is the HR. MCP3/CCL7: monocyte chemotactic protein 3; ANGPT2: angiopoietin-2; IL18: interleukin-18; IL6: interleukin-6; LAMP3: lysosome-associated membrane glycoprotein 3; CCL3: C-C motif chemokine 3; CD4: T cell surface glycoprotein; CD8A: T cell surface glycoprotein CD8 alpha chain; HO1: haeme oxygenase 1; HGF: hepatocyte growth factor; GZMA: granzyme A; CRTAM: cytotoxic and regulatory T cell molecule

When examining the shape of associations for proteins identified as being associated with pancreatic cancer risk among proteins with <500 individuals with values below LOD (Figure 3), the associations appear monotonic and broadly consistent with a linear increase in risk. Moreover, some of the proteins not found to be significantly associated with risk of pancreatic cancer in the analysis of linear associations show monotonic trends with risk (Supplementary Figures S6 and S7, available as Supplementary data at *IJE* online), such as Galectin 1 [Gal-1], CD40, TNFRSF9 and programmed cell death protein 1 [PDCD1].

### Time-varying associations

Inspection of plots of scaled Schoenfeld residuals and associated chi square tests showed evidence of time-varying associations for some proteins (Supplementary Figure S8, available as Supplementary data at *IJE* online), but for most proteins the proportional hazards assumption was plausible (Supplementary Figure S9, available as Supplementary data at *IJE* online).

**Figure 4:**
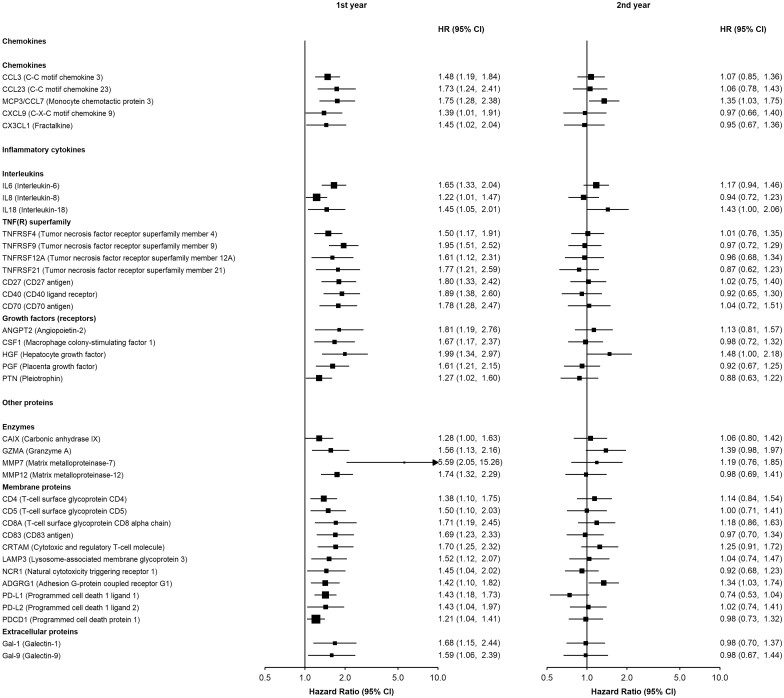
Adjusted hazard ratios for pancreatic cancer within the first and second year since study entry per standard deviation higher normalized protein expression. Models were adjusted for age, age squared, sex, smoking status, alcohol drinking, education, diabetes, and time since last meal, and stratified by region. Time in study was used as the time scale. The boxes are HRs and the horizontal lines 95% CIs. The area of the box is inversely proportional to the variance of the logHR. During the first and second years there were 39 and 47 cases, respectively.

For proteins with evidence of time-varying associations, HRs were higher in the first few years of follow-up and attenuated afterwards, as expected. Among pancreatic cancer cases, 39 (6.4%) were diagnosed within a year from baseline (i.e. when blood was collected), 47 (7.7%) at 1 to less than 2 years, 199 (32.6%) were diagnosed 2 to less than 5 years and 325 (53.3%) were diagnosed 5 years or more after blood collection. Models including an interaction of the protein with a function of time showed that for proteins showing evidence of a time-varying association, the HR was initially greater than 1 and decreased with log time, except for IL1α for which there was initially an inverse association which attenuated over time (Supplementary Table S6). When exploring the time dependence of associations by examining whether HRs varied by the number of years between blood collection and time at risk, several chemokines [CCL3, MCP3/CCL7, CCL23, fractalkine (CX3CL1), CXCL9], interleukins (IL6, IL8, IL18), members of the tumour necrosis factor superfamily (TNFRSF4, TNFRSF9, TNFRSF12A, TNFRSF21, CD27, CD40, CD70), growth factors [ANGPT2, macrophage colony-stimulating factor 1 (CSF1), HGF, placental growth factor (PGF), pleiotrophin (PTN), vascular endothelial growth factor A (VEGFA)], enzymes [carbonic anhydrase IX (CAIX), GZMA, MMP7, MMP12], membrane proteins [T cell surface glycoproteins CD4, CD5, CD8A, CD83, cytotoxic and regulatory T cell molecule (CRTAM), LAMP3, natural cytotoxicity triggering receptor 1 (NCR1), ADGRG1, angiopoietin-1 receptor (TIE2), programmed cell death 1 ligand 1 (PD-L1), programmed cell death 1 ligand 2 (PD-L2), PDCD1] and extracellular proteins [Galectin 1 (Gal-1) and 9 (Gal-9)] had time-varying associations with risk (Figure 4; and Supplementary Figure S10, available as Supplementary data at *IJE* online). Some of these proteins remained associated throughout follow-up (Supplementary Figure S11, available as Supplementary data at *IJE* online).

### Subgroup analyses

Analysis of subgroups showed a few differences in associations by age (Supplementary Figure S12, available as Supplementary data at *IJE* online), sex (Supplementary Figure S13, available as Supplementary data at *IJE* online), rural/urban residence (Supplementary Figure S14, available as Supplementary data at *IJE* online), diabetes status at baseline (Supplementary Figure S15, available as Supplementary data at *IJE* online) or regular smoking at baseline (Supplementary Figure S16, available as Supplementary data at *IJE* online). However, the number of individuals in each subgroup was relatively small and these subgroup analyses are exploratory.

### Sensitivity analysis

Using age as the underlying time scale with delayed entry at age at baseline (Supplementary Figure S17, Supplementary Tables S7 and S8, available as Supplementary data at *IJE* online), or including individuals with a history of cancer or with samples with quality control (QC) warnings or precipitation (Supplementary Table S9, available as Supplementary data at *IJE* online) showed similar results.

### Multivariable analyses for risk prediction

Sets of proteins identified using the Cox–Battey approach largely overlapped with the proteins identified in analyses where proteins were fitted one at a time (Supplementary Table S10, available as Supplementary data at *IJE* online). MCP3 and ANGPT2 were identified in all subsets.

Adding proteins to a model with established risk factors (age, age squared, sex, region, smoking, alcohol, education, diabetes and family history of cancer) led to small increases in discriminatory ability. Adding ANGPT2 and MCP3 yielded a small increase in the weighted C statistic, from 0.767 [standard error (se) 0.013] to 0.770 (se 0.013); additionally, including ARG1, IL4, IL2, CD8A, IFNβ, HO1, LAMP3, IL18, IL6, CCL3, CCL23 yielded a further small increase to 0.773 (se 0.013) and additionally including KLRD1, MIC-A/B, TNFRSF21, IL5, ADGRG1, CRTAM, CD4, MCP2, CD244, TNF, CCL19, MMP7, HGF, LAP-TGFβ1, CD40, ICOSLG, Gal1 and CXCL13 to 0.779 (se 0.013). Adding squared terms for each protein to allow for non-linear relationships yielded a C of 0.787 (se 0.013), *P* = 0.035 compared with the model which does not include proteins.

When restricting time to the first year after study entry, the model with the same established risk factors had a weighted C statistic of 0.845 (se 0.035). Adding MMP7 and IL1α yielded a weighted C of 0.888 (se 0.029), additionally adding IL4, PDCD1, ARG1, CD70, TRAIL, PD-L2, IL13, CCL23, CSF1, IL6, ANGPT2, IFNβ, MMP12, TNFRSF9, MCP3, CD27, CD40 and Gal-1 increased it to 0.921 (se 0.024), and further adding LAMP3, LAP TGF β1, GZMA, CXCL10, IL8, TNFRSF12A, CD4, FGF2, IL33, CD28, NCR1, MCP2, CRTAM, CD83 and HGF increased it to 0.939 (se 0.018, *P* = 0.002 compared with base model) (Table 2). Adding squared terms for all proteins yielded a C of 0.990 (se 0.007), but this model may be unstable due to the large number of explanatory variables and relatively small number of events.

**Table 2:** Exploratory investigation of discriminatory ability of sets of proteins to predict long- and short-term risk of incident pancreatic cancer

Variables included	Weighted C statistic (se)	95% CI	P*
Long term risk^a^			
Age, age squared, sex, region, smoking, alcohol, education, diabetes and family history of cancer	0.767 (0.013)	(0.74, 0.79)	–
+ ANGPT2, MCP3	0.770 (0.013)	(0.74, 0.80)	0.42
+ ARG1, IL4, IL2, CD8A, IFNβ, HO1, LAMP3, IL18, IL6, CCL3, CCL23	0.773 (0.013)	(0.75, 0.80)	0.31
+ KLRD1, MIC-A/B, TNFRSF21, IL5, ADGRG1, CRTAM, CD4, MCP2, CD244, TNF, CCL19, MMP7, HGF, LAP-TGFβ1, CD40, ICOSLG, Gal-1, CXCL13	0.779 (0.013)	(0.75, 0.80)	0.10
+ squared terms^b^	0.787 (0.013)	(0.76, 0.81)	0.035
Short-term risk (first year)^c^			
Age, age squared, sex, region, smoking, alcohol, education, diabetes and family history of cancer	0.845 (0.035)	(0.78, 0.91)	–
+ MMP7, IL1α	0.888 (0.029)	(0.83, 0.94)	0.09
+ IL4, PDCD1, ARG1, CD70, TRAIL, PD-L2, IL13, CCL23, CSF1, IL6, ANGPT2, IFNβ, MMP12, TNFRSF9, MCP3, CD27, CD40, Gal-1	0.921 (0.024)	(0.87, 0.97)	0.007
+ LAMP3, LAP TGF β1, GZMA, CXCL10, IL8, TNFRSF12A, CD4, FGF2, IL33, CD28, NCR1, MCP2, CRTAM, CD83, HGF	0.939 (0.018)	(0.90, 0.97)	0.002
+ squared terms^b^	0.990 (0.007)	(0.98, 1.00)	4.5 × 10^−5^

a 608 cases (two individuals have missing values for one protein each) and 623 subcohort members included.

b Adding squared terms for all proteins (to allow for non-linear relationships).

c 39 cases and 623 subcohort members included.

* *P*-value for comparison with model with age, age squared, sex, region, smoking, alcohol, education, diabetes and family history of cancer.

## Discussion

In this case-subcohort study of Chinese adults, several protein biomarkers were shown to be associated with pancreatic cancer risk, including chemokines, interleukins, growth factors, enzymes and membrane proteins, with most showing a dose-response association. Some of the associations varied over follow-up time, suggesting that the associated risks may be elevated in the years preceding diagnosis and these proteins may therefore have potential utility in predicting short-term risk. Multivariable analyses showed that adding these protein markers to conventional risk factors may lead to some improvement in discrimination when predicting pancreatic cancer risk, particularly in the short term.

Some of the markers that we have found to be associated with a higher risk throughout follow-up have been shown to be implicated in pancreatic disease. For example, MCP3/CCL7, IL4 and IL3 have been previously shown to be involved in the tumour microenvironment of pancreatic ductal adenocarcinoma and play a complex role in the regulation of tumour-promoting inflammation.^27^ ANGPT2 is a vascular growth factor involved in angiogenesis, one of the main hallmarks of cancer.^28^ It has been considered as a target for antiangiogenic therapy^29^ and shown to be secreted by hepatocellular carcinoma exosomes, small extracellular vesicles which are involved in the communication between cells.^30^ The *ANGPT2* gene has been shown to be mutated in pancreatic neuroendocrine tumours in Asian patients.^31^ Furthermore, ANGPT2-TIE2 signalling has been shown to be involved in tumour resistance to anti-VEGFA therapy^32^ and in metastasis of neuroendocrine tumours.^33^ In addition, prior studies have implicated IL18 in pancreatitis and pancreatic cancer^34^ and higher serum levels of IL18 have been shown to be associated with prognosis in pancreatic adenocarcinoma patients.^35^ Another interleukin, IL6 has also been implicated in pancreatic cancer and has been shown to be associated with a poorer prognosis^36^ and disease progression,^37^ and its receptor is being explored as a potential drug target for the disease.^38^ However, a nested case-control study within the EPIC cohort found no evidence of an association of IL6 with risk of pancreatic cancer, but found weak evidence of associations for members of the TNF superfamily.^39^ Similarly, a pooled analysis of five prospective cohort studies involving 470 pancreatic cancer cases found no evidence of an association of IL6, C-reactive protein (CRP) or TNFα receptor 2 with pancreatic cancer risk.^40^ The difference in findings for IL6 compared with the present study may be due to the association being driven by higher levels of IL6 in the time preceding diagnosis. CCL3 and other CC chemokines have complex roles in the tumour microenvironment.^41^ LAMP3 has not been previously studied in relation to pancreatic cancer, but lysosome-associated membrane proteins are involved in autophagy and have been proposed to have functions in tumour progression and metastatic spread.^42^

Among markers found to be associated with short-term risk in the present study, MMP7 had the greatest magnitude of association. MMP7 is involved in the injury response of mucosal epithelia and the degradation of extracellular matrix components and has been previously shown to be overexpressed in pancreatic ductal adenocarcinoma and its precursors, PanIN and intraductal papillary mucinous neoplasms, with MMP7 changes apparent even in intermediate-grade PanIN.^43^ In cancer, the programmed death 1 (PD-1) protein binds the ligands PD-L1 and PD-L2 to attenuate T cell receptor signalling, thus allowing the tumour to evade the cytotoxic T cell response.^44^ PD-L1 is one of the main targets of immune checkpoint inhibitors and pembrolizumab, an anti PD-1 monoclonal antibody, is effective in some pancreatic cancers with DNA mismatch repair deficiencies.^45^ This pathway is considered as a potential target for the development of immunotherapy for pancreatic cancer.^46^ NCR1 is one of the activating receptors of natural killer cells and has been considered as a target to make the immune system recognize cancer cells.^47^

When we combined proteins with conventional risk factors, only small increases in the discrimination were achieved but when restricting analyses to the first year of follow-up, the increase was substantial, suggesting potential utility of these biomarkers for short-term prediction. Early detection, even if just a few months-years prior to conventional diagnosis, may be beneficial to patients and facilitate surgical resection. Further examination of the potential utility of the markers identified and mechanisms underlying these associations is warranted. Such markers may be used in combination with other risk factors to develop risk prediction models in order to identify individuals at an increased risk of pancreatic cancer who may benefit from screening or surveillance programmes. Future studies are required to assess whether these markers are useful for longitudinal surveillance of high-risk individuals, or as diagnostic biomarkers, to help distinguish pancreatic cancer from differential diagnoses in symptomatic individuals, perhaps in combination with existing biomarkers such as CA19-9, potentially complementing other diagnostic modalities.

The differences between the markers associated with short-term and with long-term risk are likely due to changes in protein levels in the presence of yet undiagnosed pancreatic cancer or the presence of precursor lesions. Whether markers associated with long-term risk are causally related to risk of pancreatic cancer or are only markers of a long natural history of the disease needs to be assessed in further studies, employing genetic epidemiological studies such as Mendelian randomization.^48^

The primary strength of the study is its prospective design; the use of blood samples drawn before diagnosis of pancreatic cancer allows the identification of biomarkers present up to several years before its diagnosis. The study also has limitations. First, even though our study includes a relatively large number of incident cases of pancreatic cancer, given the relatively low incidence rate of this cancer, the sample size might not be large enough to identify some associations of more modest magnitude, in particular when investigating time-varying relationships. Second, although the majority of these cancers are likely to be pancreatic ductal adenocarcinoma,^49^ we do not have detailed information on histological subtypes or on stage at diagnosis for all cases. Third, we only measured 92 proteins, which is a small proportion of the proteome and does not include CA19-9. Fourth, we did not have data to independently validate our findings. However, a recent paper^50^ used the same panel in a case-control study of patients with pancreatic ductal adenocarcinoma (PDAC), patients with premalignant conditions and healthy controls, and identified markers which were associated with PDAC which largely overlapped with our findings (Supplementary Table S11).

In summary, we have identified a number of protein biomarkers that are associated with future risk of pancreatic cancer and a set of proteins which are associated with higher short-term risk. Future studies are warranted to replicate our findings and assess the potential utility of proteins in predicting the risk of pancreatic cancer, among both unselected and high-risk individuals, and in aiding the diagnostic process. Moreover, future studies could assess larger panels of proteins, as identifying more proteins associated with risk may improve our ability to predict future risk of pancreatic cancer. Additionally, our findings may provide motivation to characterize the mechanistic roles these proteins may play in the development and progression of pancreatic cancer, and future studies are needed to assess whether they could represent therapeutic targets.

## Members of the China Kadoorie Biobank Collaborative Group

International Steering Committee: Junshi Chen, Zhengming Chen (PI), Robert Clarke, Rory Collins, Yu Guo, Liming Li (PI), Chen Wang, Jun Lv, Richard Peto, Robin Walters.

International coordinating Centre, Oxford: Daniel Avery, Ruth Boxall, Derrick Bennett, Ka Hung Chan, Yumei Chang, Yiping Chen, Zhengming Chen, Jonathan Clarke, Robert Clarke, Huaidong Du, Zammy Fairhurst-Hunter, Simon Gilbert, Alex Hacker, Mike Hill, Michael Holmes, Pek Kei Im, Andri Iona, Maria Kakkoura, Christiana Kartsonaki, Rene Kerosi, Kuang Lin, Iona Millwood, Qunhua Nie, Alfred Pozarickij, Paul Ryder, Sam Sansome, Dan Schmidt, Saredo Said, Paul Sherliker, Rajani Sohoni, Becky Stevens, Iain Turnbull, Robin Walters, Lin Wang, Neil Wright, Ling Yang, Xiaoming Yang, Pang Yao.

National coordinating Centre, Beijing: Yu Guo, Xiao Han, Can Hou, Chun Li, Chao Liu, Jun Lv, Pei Pei, Canqing Yu.

Regional coordinating Centres: Guangxi Provincial CDC: Naying Chen, Duo Liu, Zhenzhu Tang. Liuzhou CDC: Ningyu Chen, Qilian Jiang, Jian Lan, Mingqiang Li, Yun Liu, Fanwen Meng, Jinhuai Meng, Rong Pan, Yulu Qin, Ping Wang, Sisi Wang, Liuping Wei, Liyuan Zhou; Gansu Provincial CDC: Caixia Dong, Pengfei Ge, Xiaolan Ren; Maiji CDC: Zhongxiao Li, Enke Mao, Tao Wang, Hui Zhang, Xi Zhang; Hainan Provincial CDC: Jinyan Chen, Ximin Hu, Xiaohuan Wang; Meilan CDC: Zhendong Guo, Huimei Li, Yilei Li, Min Weng, Shukuan Wu; Heilongjiang Provincial CDC: Shichun Yan, Mingyuan Zou, Xue Zhou; Nangang CDC: Ziyan Guo, Quan Kang, Yanjie Li, Bo Yu, Qinai Xu; Henan Provincial CDC: Liang Chang, Lei Fan, Shixian Feng, Ding Zhang, Gang Zhou; Huixian CDC: Yulian Gao, Tianyou He, Pan He, Chen Hu, Huarong Sun, Xukui Zhang; Hunan Provincial CDC: Biyun Chen, Zhongxi Fu, Yuelong Huang, Huilin Liu, Qiaohua Xu, Li Yin; Liuyang CDC: Huajun Long, Xin Xu, Hao Zhang, Libo Zhang; Jiangsu Provincial CDC: Jian Su, Ran Tao, Ming Wu, Jie Yang, Jinyi Zhou, Yonglin Zhou; Suzhou CDC: Yihe Hu, Yujie Hua, Jianrong Jin Fang Liu, Jingchao Liu, Yan Lu, Liangcai Ma, Aiyu Tang, Jun Zhang; Qingdao Qingdao CDC: Liang Cheng, Ranran Du, Ruqin Gao, Feifei Li, Shanpeng Li, Yongmei Liu, Feng Ning, Zengchang Pang, Xiaohui Sun, Xiaocao Tian, Shaojie Wang, Yaoming Zhai, Hua Zhang; Licang CDC: Wei Hou, Silu Lv, Junzheng Wang; Sichuan Provincial CDC: Xiaofang Chen, Xianping Wu, Ningmei Zhang, Weiwei Zhou; Pengzhou CDC: Xiaofang Chen, Jianguo Li, Jiaqiu Liu, Guojin Luo, Qiang Sun, Xunfu Zhong; Zhejiang Provincial CDC: Weiwei Gong, Ruying Hu, Hao Wang, Meng Wan, Min Yu; Tongxiang CDC: Lingli Chen, Qijun Gu, Dongxia Pan, Chunmei Wang, Kaixu Xie, Xiaoyi Zhang.

## Ethics approval

Ethics approval from the Oxford University Tropical Research Ethics Committee, the Chinese Centre for Disease Control and Prevention (CDC) Ethical Review Committee and the local CDC of each study area were obtained, and all participants provided written informed consent.

## Author contributions

C.K. drafted the manuscript, designed the case-subcohort study and conducted statistical analysis. M.V.H., Z.C., C.K., R.C., R.C.T., L.L., I.M., R.W., L.Y. obtained funding for the proteomics assays. Y.P., I.M., L.Y., Y.G., J.L., M.H., C.Y., Y.C., X.C., E.O.N., J.C. were involved in data acquisition and management and interpretation of results. All authors reviewed, edited and approved the manuscript.

## Data availability

The China Kadoorie Biobank (CKB) is a global resource for the investigation of lifestyle, environmental, blood biochemical and genetic factors as determinants of common diseases. The CKB study group is committed to making the cohort data available to the scientific community in China, the UK and worldwide to advance knowledge about the causes, prevention and treatment of disease. For detailed information on what data are currently available to open access users and how to apply for it, visit: [http://www.ckbiobank.org/site/Data+Access]. Researchers who are interested in obtaining from the China Kadoorie Biobank study the raw data that underlie this paper should contact [ckbaccess@ndph.ox.ac.uk]. A research proposal will be requested to ensure that any analysis is performed by bona fide researchers and—where data are not currently available to open access researchers—that analysis is restricted to the topic covered in this paper.

## Supplementary data

Supplementary data are available at *IJE* online.

## Funding

Baseline survey: Kadoorie Charitable Foundation, Hong Kong. Long-term continuation: UK Wellcome Trust (088158/Z/09/Z, 104085/Z/14/Z), Chinese Ministry of Science and Technology (2011BAI09B01, 2012–14), Chinese National Natural Science Foundation (81390541). For the purpose of Open Access, the author has applied a CC-BY public copyright licence to any Author Accepted Manuscript version arising from this submission. M.V.H. is supported by a British Heart Foundation Intermediate Clinical Research Fellowship (FS/18/23/33512) and the National Institute for Health Research Oxford Biomedical Research Centre. Y.P. acknowledges support from the China Postdoctoral Science Foundation (2019TQ0008 and 2020M670071). R.C.T. is supported by a Cancer Research UK programme grant (C8221/A29017). The proteomics analysis was funded by NDPH Pump Priming Award, Pancreatic Cancer UK (A102016RIFProfZChen), and Cancer Research UK Oxford Centre (C552/A17720).

## Supplementary Material

dyab274_Supplementary_Data

## References

[1] GBD 2017 Pancreatic Cancer Collaborators. The global, regional, and national burden of pancreatic cancer and its attributable risk factors in 195 countries and territories, 1990-2017: a systematic analysis for the Global Burden of Disease Study 2017. Lancet Gastroenterol Hepatol 2019;4:934–47. 31648972 10.1016/S2468-1253(19)30347-4 PMC7026711

[2] Cancer Research UK. Pancreatic Cancer Statistics. 2015. https://www.cancerresearchuk.org/health-professional/cancer-statistics/statistics-by-cancer-type/pancreatic-cancer (1 March 2020, date last accessed).

[3] Kamisawa T , Wood LD, Itoi T, Takaori K. Pancreatic cancer. Lancet 2016;388:73–85. 26830752 10.1016/S0140-6736(16)00141-0

[4] Canto MI , Hruban RH. Diagnosis: a step closer to screening for curable pancreatic cancer? Nat Rev Gastroenterol Hepatol 2015;12:431–32. 26122477 10.1038/nrgastro.2015.112

[5] Tummala P , Junaidi O, Agarwal B. Imaging of pancreatic cancer: an overview. J Gastrointest Oncol 2011;2:168–74. 22811847 10.3978/j.issn.2078-6891.2011.036 PMC3397617

[6] Antoniou AC , Cunningham AP, Peto J et al The BOADICEA model of genetic susceptibility to breast and ovarian cancers: updates and extensions. Br J Cancer 2008;98:1457–66. 18349832 10.1038/sj.bjc.6604305 PMC2361716

[7] International Agency for Research on Cancer. *IARC Monographs on the Identification of Carcinogenic Hazards to Humans*. List of Classifications. https://monographs.iarc.fr/lisT of-classifications/ (27 February 2020, date last accessed)

[8] Maisonneuve P , Lowenfels AB. Risk factors for pancreatic cancer: a summary review of meta-analytical studies. Int J Epidemiol 2015;44:186–98. 25502106 10.1093/ije/dyu240

[9] Mizrahi JD , Surana R, Valle JW, Shroff RT. Pancreatic cancer. Lancet 2020;395:2008–20. 32593337 10.1016/S0140-6736(20)30974-0

[10] Pang Y , Holmes MV, Chen Z, Kartsonaki C. A review of lifestyle, metabolic risk factors, and blood-based biomarkers for early diagnosis of pancreatic ductal adenocarcinoma. J Gastroenterol Hepatol 2019;34:330–45. 30550622 10.1111/jgh.14576 PMC6378598

[11] Greer JB , Whitcomb DC. Inflammation and pancreatic cancer: an evidence-based review. Curr Opin Pharmacol 2009;9:411–18. 19589727 10.1016/j.coph.2009.06.011

[12] Zambirinis CP , Pushalkar S, Saxena D, Miller G. Pancreatic cancer, inflammation, and microbiome. Cancer J 2014;20:195–202. 24855007 10.1097/PPO.0000000000000045 PMC4112373

[13] Kim JY , Hong S-M. Precursor lesions of pancreatic cancer. Oncol Res Treat 2018;41:603–10. 30269131 10.1159/000493554

[14] Storz P , Crawford HC. Carcinogenesis of pancreatic ductal adenocarcinoma. Gastroenterology 2020;158:2072–81. 32199881 10.1053/j.gastro.2020.02.059 PMC7282937

[15] Goonetilleke KS , Siriwardena AK. Systematic review of carbohydrate antigen (CA 19-9) as a biochemical marker in the diagnosis of pancreatic cancer. Eur J Surg Oncol 2007;33:266–70. 17097848 10.1016/j.ejso.2006.10.004

[16] Owens DK , Davidson KW, Krist AH et al; US Preventive Services Task Force. Screening for pancreatic cancer: US Preventive Services Task Force reaffirmation recommendation statement. JAMA 2019;322:438–44. 31386141 10.1001/jama.2019.10232

[17] Duffy MJ , Sturgeon C, Lamerz R et al Tumor markers in pancreatic cancer: a European Group on Tumor Markers (EGTM) status report. Ann Oncol 2010;21:441–47. 19690057 10.1093/annonc/mdp332

[18] Harsha HC , Kandasamy K, Ranganathan P et al A compendium of potential biomarkers of pancreatic cancer. PLoS Med 2009;6:e1000046. 19360088 10.1371/journal.pmed.1000046 PMC2661257

[19] Chen Z , Chen J, Collins R et al; China Kadoorie Biobank (CKB) collaborative group. China Kadoorie Biobank of 0.5 million people: survey methods, baseline characteristics and long-term follow-up. Int J Epidemiol 2011;40:1652–66. 22158673 10.1093/ije/dyr120 PMC3235021

[20] Prentice RL. A case-cohort design for epidemiologic cohort studies and disease prevention trials. Biometrika 1986;73:1–11.

[21] Schoenfeld D. Partial residuals for the proportional hazards regression model. Biometrika 1982;69:239–41.

[22] Xue X , Xie X, Gunter M et al Testing the proportional hazards assumption in case-cohort analysis. BMC Med Res Methodol 2013;13:88. 23834739 10.1186/1471-2288-13-88 PMC3710085

[23] Cox DR , Kartsonaki C. On the analysis of large numbers of p-values. Int Stat Rev 2019;87:505–13.

[24] Benjamini Y , Hochberg Y. Controlling the false discovery rate: a practical and powerful approach to multiple testing. J R Stat Soc Ser B Methodol 1995;57:289–300.

[25] Cox DR , Battey HS. Large numbers of explanatory variables, a semi-descriptive analysis. Proc Natl Acad Sci U S A 2017;114:8592–95. 28739925 10.1073/pnas.1703764114 PMC5559019

[26] Sanderson J , Thompson SG, White IR, Aspelund T, Pennells L. Derivation and assessment of risk prediction models using case-cohort data. BMC Med Res Methodol 2013;13:113. 24034146 10.1186/1471-2288-13-113 PMC3848813

[27] De Monte L , Wörmann S, Brunetto E et al Basophil recruitment into tumor-draining lymph nodes correlates with Th2 inflammation and reduced survival in pancreatic cancer patients. Cancer Res 2016;76:1792–803. 26873846 10.1158/0008-5472.CAN-15-1801-T

[28] Chen Z , Zhu S, Hong J et al Gastric tumor-derived ANGPT2 regulation by DARPP32 promotes angiogenesis. Gut 2016;65:925–34. 25779598 10.1136/gutjnl-2014-308416 PMC4573388

[29] Rigamonti N , De Palma M. A role for angiopoietin-2 in organ-specific metastasis. Cell Rep 2013;4:621–23. 23993444 10.1016/j.celrep.2013.07.034

[30] Xie J-Y , Wei J-X, Lv L-H et al Angiopoietin-2 induces angiogenesis via exosomes in human hepatocellular carcinoma. Cell Commun Signal 2020;18:46. 32183816 10.1186/s12964-020-00535-8 PMC7077328

[31] Chou W-C , Lin P-H, Yeh Y-C et al Genes involved in angiogenesis and mTOR pathways are frequently mutated in Asian patients with pancreatic neuroendocrine tumors. Int J Biol Sci 2016;12:1523–32. 27994516 10.7150/ijbs.16233 PMC5166493

[32] Rigamonti N , Kadioglu E, Keklikoglou I, Wyser Rmili C, Leow CC, De Palma M. Role of angiopoietin-2 in adaptive tumor resistance to VEGF signaling blockade. Cell Rep 2014;8:696–706. 25088418 10.1016/j.celrep.2014.06.059

[33] Melen-Mucha G , Niedziela A, Mucha S et al Elevated peripheral blood plasma concentrations of tie-2 and angiopoietin 2 in patients with neuroendocrine tumors. Int J Mol Sci 2012;13:1444–60. 22408401 10.3390/ijms13021444 PMC3291970

[34] Li Z , Yu X, Werner J, Bazhin AV, D’Haese JG. The role of interleukin-18 in pancreatitis and pancreatic cancer. Cytokine Growth Factor Rev 2019;50:1–12. 31753718 10.1016/j.cytogfr.2019.11.001

[35] Carbone A , Vizio B, Novarino A et al IL-18 paradox in pancreatic carcinoma: elevated serum levels of free IL-18 are correlated with poor survival. J Immunother 2009;32:920–31. 19816189 10.1097/CJI.0b013e3181b29168

[36] Palmquist C , Dehlendorff C, Calatayud D, Hansen CP, Hasselby JP, Johansen JS. Prediction of unresectability and prognosis in patients undergoing surgery on suspicion of pancreatic cancer using carbohydrate antigen 19-9, interleukin 6, and YKL-40. Pancreas 2020;49:53–61. 31856080 10.1097/MPA.0000000000001466

[37] Ramsey ML , Talbert E, Ahn D et al Circulating interleukin-6 is associated with disease progression, but not cachexia in pancreatic cancer. Pancreatology 2019;19:80–87. 30497874 10.1016/j.pan.2018.11.002 PMC6613190

[38] Long KB , Tooker G, Tooker E et al IL6 receptor blockade enhances chemotherapy efficacy in pancreatic ductal adenocarcinoma. Mol Cancer Ther 2017;16:1898–908. 28611107 10.1158/1535-7163.MCT-16-0899 PMC5587413

[39] Grote VA , Kaaks R, Nieters A et al Inflammation marker and risk of pancreatic cancer: a nested case-control study within the EPIC cohort. Br J Cancer 2012;106:1866–74. 22617158 10.1038/bjc.2012.172 PMC3364108

[40] Bao Y , Giovannucci EL, Kraft P et al Inflammatory plasma markers and pancreatic cancer risk: a prospective study of five U.S. cohorts. Cancer Epidemiol Biomarkers Prev 2013;22:855–61. 23462920 10.1158/1055-9965.EPI-12-1458 PMC3650127

[41] Zambirinis CP , Miller G. Cancer manipulation of host physiology – lessons from pancreatic cancer. Trends Mol Med 2017;23:465–81. 28400243 10.1016/j.molmed.2017.03.003 PMC5480288

[42] Alessandrini F , Pezzè L, Ciribilli Y. LAMPs: shedding light on cancer biology. Semin Oncol 2017;44:239–53. 29526252 10.1053/j.seminoncol.2017.10.013

[43] Buchholz M , Braun M, Heidenblut A et al Transcriptome analysis of microdissected pancreatic intraepithelial neoplastic lesions. Oncogene 2005;24:6626–36. 16103885 10.1038/sj.onc.1208804

[44] Keenan TE , Burke KP, Van Allen EM. Genomic correlates of response to immune checkpoint blockade. Nat Med 2019;25:389–402. 30842677 10.1038/s41591-019-0382-x PMC6599710

[45] Marabelle A , Le DT, Ascierto PA et al Efficacy of pembrolizumab in patients with noncolorectal high microsatellite instability/mismatch repair-deficient cancer: results from the phase II KEYNOTE-158 study. J Clin Oncol 2020;38:1–10. 31682550 10.1200/JCO.19.02105 PMC8184060

[46] Yang C-Y , Fan MH, Miao CH, Liao YJ, Yuan R-H, Liu CL. Engineering chimeric antigen receptor T cells against immune checkpoint inhibitors PD-1/PD-L1 for treating pancreatic cancer. Mol Ther Oncolytics 2020;17:571–85. 32637575 10.1016/j.omto.2020.05.009 PMC7321819

[47] Tal Y , Yaakobi S, Horovitz-Fried M et al An NCR1-based chimeric receptor endows T cells with multiple anti-tumor specificities. Oncotarget 2014;5:10949–58. 25431955 10.18632/oncotarget.1919 PMC4279421

[48] Holmes MV, , Ala-Korpela M, , Smith GD. Mendelian randomization in cardiometabolic disease: challenges in evaluating causality. Nat Rev Cardiol 2017;14:577–90. 28569269 10.1038/nrcardio.2017.78 PMC5600813

[49] Luo G , Fan Z, Gong Y et al Characteristics and outcomes of pancreatic cancer by histological subtypes. Pancreas 2019;48:817–22. 31210663 10.1097/MPA.0000000000001338

[50] Lindgaard SC , Sztupinszki Z, Maag E et al Circulating protein biomarkers for use in pancreatic ductal adenocarcinoma identification. Clin Cancer Res 2021;27:2592–603. 33737308 10.1158/1078-0432.CCR-20-4215

