# Supplementary material for: Circulating proteins and risk of pancreatic cancer: a case-subcohort study among Chinese adults
Source: Int J Epidemiol. Author manuscript; Available in PMC 2022 Jun 16. (PMC9189974; doi:10.1093/ije/dyab274)

**Supplementary material**

**Circulating proteins and risk of pancreatic cancer: a case-subcohort study among Chinese adults**

Christiana Kartsonaki^1, 2^, Yuanjie Pang^3^, Iona Millwood^1, 2^, Ling Yang^1, 2^, Yu Guo^4^, Robin Walters^1, 2^, Jun Lv^3^, Michael Hill^1^, Canqing Yu^3^, Yiping Chen^1, 2^, Xiaofang Chen^5^, Eric O’ Neill^6^, Junshi Chen^7^, Ruth C. Travis^8^, Robert Clarke^1^, Liming Li^3^, Zhengming Chen^1, 2^, Michael V. Holmes^1, 2, 9^

1. Clinical Trial Service Unit & Epidemiological Studies Unit (CTSU), Nuffield Department of Population Health, Big Data Institute Building, Roosevelt Drive, University of Oxford, UK

2. Medical Research Council Population Health Research Unit (MRC PHRU), Nuffield Department of Population Health, University of Oxford, UK

3. Department of Epidemiology and Biostatistics, School of Public Health, Peking University, 38 Xueyuan Road, Beijing 100191, China

4. Chinese Academy of Medical Sciences, 9 Dongdan San Tiao, Beijing 100730, China

5. NCDs Prevention and Control Department, Pengzhou CDC

6. Department of Oncology, University of Oxford, UK

7. National Center for Food Safety Risk Assessment, 37 Guangqu Road, Beijing 100021, China

8. Cancer Epidemiology Unit (CEU), Nuffield Department of Population Health, Richard Doll Building, Roosevelt Drive, University of Oxford, UK

9. National Institute for Health Research Oxford Biomedical Research Centre, John Radcliffe University Hospital, Oxford, UK

Address for correspondence:

Christiana Kartsonaki

MRC PHRU

NDPH, Big Data Institute Building

University of Oxford

Old Road Campus

Oxford, OX3 7LF, UK

Tel: 44-1865-743644

Fax: 44-1865-743985

December 2021

Contents

[Supplementary Methods 3](#_Toc93356601)

[Table S1. List of 92 proteins quantified by the OLINK immuno-oncology assay and biological processes in which they are involved 7](#_Toc93356602)

[Table S2. Grouping of the 92 proteins measured by OLINK immuno-oncology assay 12](#_Toc93356603)

[Table S3. Limit of detection (LOD) of 92 proteins 16](#_Toc93356604)

[Table S4. Hazard ratios (95% CIs) for all proteins (per SD higher NPX), adjusted for age, age^2^, sex, region, smoking, alcohol, education, diabetes, fasting time and stratified by region, with time in study as the time scale 18](#_Toc93356605)

[Table S5. Hazard ratios (95% CIs) for all proteins (per SD higher NPX), adjusted for age and sex and stratified by region, with time in study as the time scale 20](#_Toc93356606)

[Table S6. Hazard ratios (95% CIs) for all proteins (per SD higher NPX) and their interaction with time since sample collection, adjusted for age, age^2^, sex, region, smoking, alcohol drinking, education, diabetes and time since last meal, with time in study as the time scale 22](#_Toc93356607)

[Table S7. Hazard ratios (95% CIs) for all proteins (per SD higher NPX), adjusted for age and sex and stratified by region, with age as the time scale and entry at age at baseline 27](#_Toc93356608)

[Table S8. Hazard ratios (95% CIs) for all proteins (per SD higher NPX), adjusted for age, age^2^, sex, region, smoking, alcohol, education, diabetes, fasting time and stratified by region, with age as the time scale and entry at age at baseline 30](#_Toc93356609)

[Table S9. Hazard ratios (95% CIs) for all proteins (per SD higher NPX), adjusted for age and sex and stratified by region, with time in study as the time scale; no exclusions (using all individuals) 32](#_Toc93356610)

[Table S10. Hazard ratios (95% CIs) for all proteins (per SD higher NPX) from multivariable models, identified using the Cox–Battey approach, adjusted for age, age^2^, sex, smoking, alcohol drinking, education, diabetes, time since last meal, and family history of cancer, stratified by region, with time in study as the time scale. 35](#_Toc93356611)

[Table S11. Comparison of the findings of Lindgaard et al. and of the present study 37](#_Toc93356612)

[Figure S1. Histograms of proteins 40](#_Toc93356613)

[Figure S2. Correlations between proteins in the subcohort 42](#_Toc93356614)

[Figure S3. Hazard ratios (95% CI) for selected proteins (for a SD higher NPX), adjusted for age, sex and stratified by region. 43](#_Toc93356615)

[Figure S4. Hazard ratios (95% CI) for NPX greater than versus less than or equal to the limit of detection, for proteins for which at least 500 individuals had values below the limit of detection. 44](#_Toc93356616)

[Figure S5. Adjusted hazard ratios for pancreatic cancer associated with per SD higher NPX by category of proteins. 45](#_Toc93356617)

[Figure S6. Hazard ratios (95% CI) for all proteins with at least 500 individuals with values over the limit of detection, by NPX split at quartiles. 46](#_Toc93356618)

[Figure S7. Spline fits for NPX for all proteins with at least 500 individuals with values over the limit of detection. Models were adjusted for age, age^2^, sex, smoking status, alcohol drinking, education, diabetes, time since last meal and stratified by region. Time in study was used as the time scale. x axis values are NPX divided by its standard deviation. 47](#_Toc93356619)

[Figure S8. Scaled Schoenfeld residuals for selected proteins for which there is evidence of time dependence of hazard ratios. 48](#_Toc93356620)

[Figure S9. Scaled Schoenfeld residuals for selected proteins for which there is no evidence of time dependence of hazard ratios. 49](#_Toc93356621)

[Figure S10. Transformed p-values against their expected values for associations within the first year of follow-up. 55](#_Toc93356622)

[Figure S11. Hazard ratios (95% CI) per SD higher NPX by time since study entry. 56](#_Toc93356623)

[Figure S12. Hazard ratios (95% CI) per SD higher NPX in individuals aged <60 and ≥60 years at sample collection. 58](#_Toc93356624)

[Figure S13. Hazard ratios (95% CI) per SD higher NPX in males and females. 59](#_Toc93356625)

[Figure S14. Hazard ratios (95% CI) per SD higher NPX in individuals from rural and urban regions. 60](#_Toc93356626)

[Figure S15. Hazard ratios (95% CI) per SD higher NPX by diabetes status at sample collection. 61](#_Toc93356627)

[Figure S16. Hazard ratios (95% CI) per SD higher NPX by smoking status at sample collection. 62](#_Toc93356628)

[Figure S17. Hazard ratios (95% CI) for selected proteins (for a SD higher NPX), with age as the time scale and entry at age at baseline. 63](#_Toc93356629)

#

# Supplementary Methods

**Data collection**

At local study assessment clinics, participants completed an interviewer-administered laptop-based questionnaire on sociodemographic characteristics, smoking, alcohol consumption, diet, tea drinking, physical activity, personal and family medical history, and current medication. Personal medical history included history of diabetes, coronary heart disease, stroke, cancer, and other major conditions. Family medical history included diabetes, coronary heart disease, stroke, psychiatric disorders, and cancer in parents, siblings, or children. A range of physical measurements were recorded by trained technicians, including height, weight, hip and waist circumference, bioimpedance, lung function, blood pressure, and heart rate.

Standing height was measured to the nearest 0.1 cm using a stadiometer. Weight was measured to the nearest 0.1 kg using a body composition analyser (TANITA-TBF-300GS; Tanita), with subtraction of weight of clothing by 0.5 kg in summer, 1.0 kg in spring/autumn and 2.0–2.5 kg in winter. BMI was calculated as the measured weight in kilograms divided by the square of the measured height in metres.

A 10‐ml non‐fasting (with the time since the participant last ate recorded) blood sample was collected from participants into an EDTA vacutainer (BD Hemogard^TM^, USA). Immediate on‐site testing of random plasma glucose (RPG) level was undertaken using the SureStep Plus System (Johnson & Johnson), regularly calibrated with manufacturer quality control solution. Participants with glucose levels ≥7.8 mmol/L and <11.1 mmol/L were invited to return for a fasting plasma glucose (FPG) test the next day ^1^. RPG data were unavailable for 8,306 participants due to a supply issue. Previously diagnosed diabetes was defined by the question 'Has a doctor ever told you that you had diabetes?'. Among those without previously diagnosed diabetes, screen‐detected diabetes was defined as RPG ≥7.0 mmol/L and time since last eating ≥8 h, or ≥11.1 mmol/L with time since last eating <8 h, or a FPG ≥7.0 mmol/L on subsequent testing ^2^. For those with previously diagnosed diabetes, duration of diabetes was defined as the time interval between diagnosis and baseline visit.

Smoking status was classified as never (smoked <100 cigarettes in lifetime), occasional, former, or regular smoker ^3^. Former smokers were defined as those who had smoked a total of at least 100 cigarettes or equivalent but had quit smoking for ≥6 months. Regular smokers were defined as those who reported having ever smoked one or more cigarettes (or their equivalent) daily for at least 6 months. Among regular smokers who had stopped 6 or more months before recruitment, approximately half did so because of ill health, and they were still counted as smokers in the main analyses. Among regular smokers, the amount of smoking was categorized as <20, 20–25, and ≥25 cigarettes per day.

Data collected on alcohol drinking included whether the participant had drunk alcohol regularly (i.e. at least once a week on a regular basis) during the past 12 months. Abstainers were defined as those who had never or almost never drank alcohol in the past 12 months and had not drunk weekly in the past. Occasional drinkers were defined as those who in the past 12 months had drunk alcohol occasionally, during certain seasons, or monthly but less than weekly, and had not drunk weekly in the past. Reduced-intake drinkers were those who in the past 12 months had drunk alcohol occasionally, during certain seasons, or monthly but less than weekly, but had drunk weekly in the past. Ex-weekly drinkers were those who had drunk weekly in the past but had never or almost never drank alcohol in the past 12 months. Weekly drinkers were those who usually drank at least once a week during the past 12 months.

**Mortality and morbidity follow-up**

The vital status of each participant was determined periodically through China CDC's Disease Surveillance Points (DSP) system ^4^, supplemented by regular checks against local residential records and health insurance records and by annual active confirmation through street committees or village administrators. In addition, information about occurrence of major diseases and any episodes of hospitalisation was collected through linkage, via each participant's unique national identification number, with disease registries (for cancer, ischemic heart disease, stroke, and diabetes) and the national health insurance system which has almost universal coverage in the study areas. All events were coded using International Classification of Diseases 10th Revision (ICD-10) by trained staff who were blinded to baseline information. By 1.1.2016, 37,289 (7.3%) participants had died, 4,875 (1.0%) were lost to follow-up and 24,414 (4.8%) had developed cancer, including 702 (0.1%) with pancreatic cancer (ICD-10 C25).

**Measurement of protein biomarkers**

Blood samples were collected at baseline using standardised procedures and shipped on dry ice to Oxford for long-term storage in liquid nitrogen tanks ^5^. Stored baseline plasma samples for the cases and subcohort were retrieved, thawed, and subaliquoted at the Wolfson laboratory, CTSU, and 100 µL aliquots were shipped on dry ice to the OLINK Laboratory at Uppsala, Sweden, for the OLINK Immuno-Oncology assay (Olink Biosciences, Uppsala, Sweden). The assay uses a 96-well microplate format, including 90 samples and six external quality control standards. Each sample is mixed with 92 pairs of oligonucleotide-labelled antibodies and four internal technical controls. When both high-specificity antibodies bind the target protein in the sample, the attached oligonucleotides form a unique DNA reporter sequence that is subsequently amplified and quantified with standard PCR. Samples were randomly distributed on plates with laboratory staff blinded to case status and other participant information. PCR values above the fluorescence detection threshold were log2-transformed and corrected for technical variation based on negative and inter-plate controls (IPC). The lower limits of detection (LOD) were determined using negative control samples (buffer without antigen). Details of the OLINK assay have been reported elsewhere, including measurements of assay performance and validation ^6–8^.

**Statistical analysis**

Principal component analysis was used to examine the data and detect individuals with extreme values. Histograms were plotted to visually inspect the distributions of proteins. We calculated Pearson’s correlations between proteins.

Interleukin-21 (IL21) and interleukin-35 (IL35) were excluded because almost all participants had values below the lower LOD, leaving 90 proteins for all analyses. However, the 4 individuals with detectable values of IL21, one of whom also had detectable IL35, were pancreatic cancer cases. Of the 90 proteins, for 13 proteins (interleukin-1α [IL1α], adhesion G-protein coupled receptor G1 [ADGRG1], fibroblast growth factor 2 [FGF2], interferon-β [IFNβ], nitric oxide synthase 3 [NOS3], interleukin-2 [IL2], interleukin-33 [IL33], interleukin-5 [IL5], C-X-C motif chemokine 12 [CXCL12], interferon-γ [IFNγ], interleukin-4 [IL4], interleukin-13 [IL13], and tumour necrosis factor [TNF]) 500 or more individuals had values below the limit of detection. These proteins were dichotomised into whether the value was greater than the limit of detection or less than or equal to the limit of detection, in addition to the analysis treating them as continuous.

Models in the main analysis were stratified by region (10 regions) and adjusted for age (numeric), age^2^, sex, smoking status (3 groups: never, ex-regular, occasional, current regular smoking), alcohol drinking (3 groups: never regular, ex-regular and current regular drinking), educational attainment (3 groups: no formal education or primary school, middle or high school, technical school/college or university), diabetes (2 groups: no diabetes, screen-detected/self-reported diabetes) and time since last meal (numeric), and time in study was used as the time scale. We used time in study as the time scale as we considered it to be a more natural time scale due to the possibility of time-varying associations if altered values of some proteins are manifestations of subclinical disease, which would lead to proteins being associated with a higher risk of a pancreatic cancer diagnosis during the first few months/years of follow-up. Models adjusting for age and sex and stratifying by region were also fitted.

Models were fitted in subgroups by age at sample collection (<60 vs ≥60), sex, rural/urban region, diabetes, and regular smoking, to assess whether the associations differed by any of these factors, adjusting for the same variables as in the main analysis. Sensitivity analyses were conducted including the samples that had quality control warnings or precipitation (i.e. including all 1397 participants' samples assayed), and with age as the underlying time scale.

Multivariable models with several proteins were fitted using the approach of Cox and Battey ^9^. No transformations or interactions of variables were considered. The 90 markers were laid on a 5×5×4 cuboid. The remaining 10 positions were filled with normally distributed random variables with mean zero and variance one. A regression was fitted with each set of explanatory variables indexed by each dimension of the cuboid, adjusting for age, age^2^, sex, smoking, alcohol, education, diabetes, fasting time, family history of cancer and stratifying by region. The markers most highly associated with pancreatic cancer risk were kept from each regression. This was defined either as the 3 or 2 most significant, or those with │z│ > 2.5. Then for each of the four criteria markers identified as such 3 times were selected and included in a model, adjusting for the covariates above. The coefficients from the multivariable models were compared to coefficients obtained from models with one protein at a time.

To assess whether including selected proteins adds to the discriminatory ability of a model with established risk factors, four models were fitted: model 1 included age, age^2^, sex, smoking status (3 groups), alcohol drinking (3 groups), educational attainment (3 groups), diabetes (2 groups), family history of cancer (2 groups), and region; model 2 additionally included the proteins identified from the multivariable models as being most associated with risk of pancreatic cancer, that is variables selected three times from the Cox–Battey procedure when keeping those with │z│ > 2.5; model 3 additionally included proteins selected when keeping the 2 most significant; and model 4 additionally including those selected when keeping the 3 most significant­. Discrimination of risk prediction models was assessed using a weighted C-index ^10,11^. This process was repeated with time censored one year after study entry to identify combinations of proteins which may predict short-term risk of pancreatic cancer.

Analysis was done using R version 3.6.3 ^12^ and packages ‘survival’ ^13^, ‘Epi’ ^14^, ‘reshape2’ ^15(p2)^, ‘ggplot2’ ^16^, ‘ckbplotr’ ^17^ and ‘ManyTests’ ^18^.

**References**

1. Bragg F, Li L, Smith M, et al. Associations of blood glucose and prevalent diabetes with risk of cardiovascular disease in 500 000 adult Chinese: the China Kadoorie Biobank. *Diabet Med J Br Diabet Assoc*. 2014;31(5):540-551. doi:10.1111/dme.12392

2. Pang Y, Kartsonaki C, Guo Y, et al. Diabetes, plasma glucose and incidence of pancreatic cancer: A prospective study of 0.5 million Chinese adults and a meta-analysis of 22 cohort studies. *Int J Cancer*. 2017;140(8):1781-1788. doi:10.1002/ijc.30599

3. Chen Z, Peto R, Zhou M, et al. Contrasting male and female trends in tobacco-attributed mortality in China: evidence from successive nationwide prospective cohort studies. *The Lancet*. 2015;386(10002):1447-1456. doi:10.1016/S0140-6736(15)00340-2

4. Yang G, Rao C, Ma J, et al. Validation of verbal autopsy procedures for adult deaths in China. *Int J Epidemiol*. 2006;35(3):741-748. doi:10.1093/ije/dyi181

5. Chen Z, Chen J, Collins R, et al. China Kadoorie Biobank of 0.5 million people: survey methods, baseline characteristics and long-term follow-up. *Int J Epidemiol*. 2011;40(6):1652-1666. doi:10.1093/ije/dyr120

6. Assarsson E, Lundberg M, Holmquist G, et al. Homogenous 96-plex PEA immunoassay exhibiting high sensitivity, specificity, and excellent scalability. *PloS One*. 2014;9(4):e95192. doi:10.1371/journal.pone.0095192

7. Lundberg M, Thorsen SB, Assarsson E, et al. Multiplexed homogeneous proximity ligation assays for high-throughput protein biomarker research in serological material. *Mol Cell Proteomics MCP*. 2011;10(4):M110.004978. doi:10.1074/mcp.M110.004978

8. Pang Y, Kartsonaki C, Lv J, et al. Associations of Adiposity, Circulating Protein Biomarkers, and Risk of Major Vascular Diseases. *JAMA Cardiol*. 2021;6(3):276-286. doi:10.1001/jamacardio.2020.6041

9. Cox DR, Battey HS. Large numbers of explanatory variables, a semi-descriptive analysis. *Proc Natl Acad Sci*. 2017;114(32):8592-8595. doi:10.1073/pnas.1703764114

10. Ganna A, Reilly M, de Faire U, Pedersen N, Magnusson P, Ingelsson E. Risk prediction measures for case-cohort and nested case-control designs: an application to cardiovascular disease. *Am J Epidemiol*. 2012;175(7):715-724. doi:10.1093/aje/kwr374

11. Sanderson J, Thompson SG, White IR, Aspelund T, Pennells L. Derivation and assessment of risk prediction models using case-cohort data. *BMC Med Res Methodol*. 2013;13:113. doi:10.1186/1471-2288-13-113

12. R: The R Project for Statistical Computing. Accessed February 28, 2020. https://www.r-project.org/

13. Therneau TM, until 2009) TL (original S->R port and R maintainer. *Survival: Survival Analysis*.; 2019. Accessed February 28, 2020. https://CRAN.R-project.org/package=survival

14. Carstensen B, Plummer M, Laara E, Hills M. *Epi: Statistical Analysis in Epidemiology*.; 2020. Accessed November 11, 2020. https://CRAN.R-project.org/package=Epi

15. Wickham H. *Reshape2: Flexibly Reshape Data: A Reboot of the Reshape Package*.; 2020. Accessed November 11, 2020. https://CRAN.R-project.org/package=reshape2

16. Create Elegant Data Visualisations Using the Grammar of Graphics. Accessed November 11, 2020. https://ggplot2.tidyverse.org/

17. Wright N. ckbplotr: Create CKB Plots. Published 2020. Accessed November 11, 2020. https://github.com/neilstats/ckbplotr

18. Kartsonaki C. *ManyTests: Multiple Testing Procedures of Cox (2011) and Wong and Cox (2007)*.; 2016. Accessed March 1, 2020. https://CRAN.R-project.org/package=ManyTests

# Table S1. List of 92 proteins quantified by the OLINK immuno-oncology assay and biological processes in which they are involved

| **Label** | **Name** | **Biological process** | | | |  | **Linked to pancreatic cancer** |  |
| --- | --- | --- | --- | --- | --- | --- | --- | --- |
| ADA | Adenosine deaminase | M |  |  | V |  |  |  |
| ADGRG1 | Adhesion G-protein coupled receptor G1 |  |  |  |  |  |  |  |
| ANG1 | Angiopoietin-1 |  |  |  | V |  |  |  |
| ANGPT2 | Angiopoietin-2 |  |  |  | V |  | Yes |  |
| ARG1 | Arginase-1 |  |  |  |  |  | Yes |  |
| CAIX | Carbonic anhydrase IX | M |  |  | V |  | Yes |  |
| CASP8 | Caspase-8 | A |  |  |  |  | Yes |  |
| CCL3 | C-C motif chemokine 3 | C |  |  |  |  | Yes |  |
| CCL4 | C-C motif chemokine 4 | C |  |  |  |  | Yes |  |
| CCL17 | C-C motif chemokine 17 | C |  | S |  |  |  |  |
| CCL19 | C-C motif chemokine 19 | C |  | S |  |  |  |  |
| CCL20 | C-C motif chemokine 20 | C |  | S |  |  | Yes |  |
| CCL23 | C-C motif chemokine 23 | C |  |  | V |  |  |  |
| CD4 | T-cell surface glycoprotein CD4 |  | P | S |  |  |  |  |
| CD5 | T-cell surface glycoprotein CD5 |  | P | S |  |  |  |  |
| CD8A | T-cell surface glycoprotein CD8 alpha chain |  | P |  |  |  |  |  |
| CD27 | CD27 antigen |  | P |  |  |  |  |  |
| CD28 | T-cell surface glycoprotein CD28 |  | P |  |  |  |  |  |
| CD40 | CD40L receptor |  | P |  |  |  | Yes |  |
| CD40L | CD40-L | A | P |  |  |  |  |  |
| CD70 | CD70 antigen |  | P |  |  |  |  |  |
| CD83 | CD83 antigen |  | P |  |  |  | Yes |  |
| CD244 | Natural killer cell receptor 2B4 |  | P |  |  |  |  |  |
| CRTAM | Cytotoxic and regulatory T-cell molecule |  | P |  |  |  |  |  |
| CSF1 | Macrophage colony-stimulating factor 1 |  |  | S |  |  | Yes |  |
| CX3CL1 | Fractalkine | C | P |  |  |  |  |  |
| CXCL1 | C-X-C motif chemokine 1 | C |  | S | V |  |  |  |
| CXCL5 | C-X-C motif chemokine 5 | C |  | S | V |  | Yes |  |
| CXCL9 | C-X-C motif chemokine 9 | C | P |  | V |  | Yes |  |
| **Label** | **Name** | **Biological process** | | | |  | **Linked to pancreatic cancer** |  |
| CXCL10 | C-X-C motif chemokine 10 | C | P |  | V |  | Yes |  |
| CXCL11 | C-X-C motif chemokine 11 | C | P | S | V |  | Yes |  |
| CXCL12 | Stromal cell-derived factor 1 | C |  | S | V |  |  |  |
| CXCL13 | C-X-C motif chemokine 13 | C | P | S |  |  |  |  |
| DCN | Decorin |  |  |  | V |  | Yes |  |
| EGF | Pro-epidermal growth factor |  |  |  | V |  | Yes |  |
| FasL | Fas antigen ligand | A |  |  |  |  | Yes |  |
| FGF2 | Fibroblast growth factor 2 |  |  |  | V |  | Yes |  |
| Gal1 | Galectin-1 |  |  | S | V |  |  |  |
| Gal9 | Galectin-9 | A |  | S | V |  |  |  |
| GZMA | Granzyme A | A |  |  |  |  |  |  |
| GZMB | Granzyme B | A |  |  |  |  |  |  |
| GZMH | Granzyme H | A |  |  |  |  |  |  |
| HGF | Hepatocyte growth factor |  |  |  | V |  | Yes |  |
| HO1 | Heme oxygenase 1 | M |  |  |  |  |  |  |
| ICOSLG | ICOS ligand |  | P |  |  |  |  |  |
| IFN beta | Interferon beta |  | P |  |  |  |  |  |
| IFN gamma | Interferon gamma |  | P |  |  |  | Yes |  |
| IL1 alpha | Interleukin-1 alpha |  | P | S | V |  |  |  |
| IL2 | Interleukin-2 |  | P |  |  |  | Yes |  |
| IL4 | Interleukin-4 |  |  | S |  |  |  |  |
| IL5 | Interleukin-5 |  |  | S |  |  |  |  |
| IL6 | Interleukin-6 |  | P | S |  |  | Yes |  |
| IL7 | Interleukin-7 |  | P |  |  |  |  |  |
| IL8 | Interleukin-8 | C |  | S | V |  |  |  |
| IL10 | Interleukin-10 |  |  | S |  |  | Yes |  |
| IL12 | Interleukin-12 |  |  |  |  |  |  |  |
| IL12RB1 | Interleukin-12 receptor subunit beta-1 |  | P |  |  |  |  |  |
| IL13 | Interleukin-13 |  |  | S |  |  |  |  |
| IL18 | Interleukin-18 |  | P | S |  |  | Yes |  |
| IL21 | Interleukin-21 |  | P |  |  |  |  |  |
| IL33 | Interleukin-33 |  |  | S |  |  |  |  |
| IL35 | Interleukin-35 |  |  | S |  |  |  |  |
| **Label** | **Name** | **Biological process** | | | |  | **Linked to pancreatic cancer** |  |
| KLRD1 | Natural killer cells antigen CD94 |  | P |  |  |  |  |  |
| LAMP3 | Lysosome-associated membrane glycoprotein 3 | |  | S |  |  |  |  |
| LAP TGF-beta-1 | Latency-associated peptide transforming growth factor beta-1 | | | S |  |  |  |  |
| MCP1 | Monocyte chemotactic protein 1 | C |  |  | V |  |  |  |
| MCP2 | Monocyte chemotactic protein 2 | C |  |  |  |  |  |  |
| MCP3 | Monocyte chemotactic protein 3 | C |  |  |  |  |  |  |
| MCP4 | Monocyte chemotactic protein 4 | C |  | S | V |  |  |  |
| MIC-A/B | MHC class I polypeptide-related sequence A/B |  |  | S |  |  |  |  |
| MMP7 | Matrix metalloproteinase-7 | A |  | S |  |  | Yes |  |
| MMP12 | Matrix metalloproteinase-12 |  |  | S | V |  | Yes |  |
| NCR1 | Natural cytotoxicity triggering receptor 1 |  | P |  |  |  |  |  |
| NOS3 | Nitric oxide synthase, endothelial |  |  |  | V |  |  |  |
| PD L1 | Programmed cell death 1 ligand 1 |  |  | S |  |  |  |  |
| PD L2 | Programmed cell death 1 ligand 2 |  |  | S |  |  |  |  |
| PDCD1 | Programmed cell death protein 1 |  |  | S |  |  |  |  |
| PDGF subunit B | Platelet-derived growth factor subunit B |  |  |  | V |  |  |  |
| PGF | Placenta growth factor |  |  |  | V |  |  |  |
| PTN | Pleiotrophin |  |  |  | V |  |  |  |
| TIE2 | Angiopoietin-1 receptor |  |  |  | V |  |  |  |
| TNF | Tumour necrosis factor alpha |  | P | S | V |  | Yes |  |
| TNFRSF4 | Tumour necrosis factor receptor superfamily member 4 | | P |  |  |  |  |  |
| TNFRSF9 | Tumour necrosis factor receptor superfamily member 9 | | P |  |  |  |  |  |
| TNFRSF12A | Tumour necrosis factor receptor superfamily member 12A | A |  |  | V |  |  |  |
| TNFRSF21 | Tumour necrosis factor receptor superfamily member 21 | A |  |  |  |  |  |  |
| TNFSF14 | Tumour necrosis factor ligand superfamily member 14 | | P |  |  |  |  |  |
| TRAIL | TNF-related apoptosis-inducing ligand | A |  |  |  |  |  |  |
| TWEAK | Tumour necrosis factor (Ligand) superfamily, member 12 | A |  |  | V |  |  |  |
| VEGFA | Vascular endothelial growth factor A |  |  |  | V |  | Yes |  |
| VEGFC | Vascular endothelial growth factor C |  |  |  | V |  | Yes |  |
| VEGFR2 | Vascular endothelial growth factor receptor 2 |  |  |  | V |  |  |  |

Abbreviations: A, apoptosis/cell killing; C, chemotaxis; M, metabolism/autophagy; P, promote tumour immunity; S, supress tumour immunity; V, vascular & tissue remodelling.

Linked to pancreatic cancer indicates molecules listed by Harsha et al. as potential biomarkers of pancreatic cancer.

# Table S2. Grouping of the 92 proteins measured by OLINK immuno-oncology assay

| **Label, group** | **Name** | **Protein type** |
| --- | --- | --- |
| **Chemokines** |  |  |
| CCL3 | C-C motif chemokine 3 | Chemokine CC family |
| CCL4 | C-C motif chemokine 4 | Chemokine CC family |
| CCL17 | C-C motif chemokine 17 | Chemokine CC family |
| CCL19 | C-C motif chemokine 19 | Chemokine CC family |
| CCL20 | C-C motif chemokine 20 | Chemokine CC family |
| CCL23 | C-C motif chemokine 23 | Chemokine CC family |
| MCP1/CCL2 | Monocyte chemotactic protein 1 | Chemokine CC family |
| MCP2/CCL8 | Monocyte chemotactic protein 2 | Chemokine CC family |
| MCP3/CCL7 | Monocyte chemotactic protein 3 | Chemokine CC family |
| MCP4 | Monocyte chemotactic protein 4 | Chemokine CC family |
| CXCL1 | C-X-C motif chemokine 1 | Chemokine CxC family, growth factor |
| CXCL5 | C-X-C motif chemokine 5 | Chemokine CxC family |
| CXCL9 | C-X-C motif chemokine 9 | Chemokine CxC family |
| CXCL10 | C-X-C motif chemokine 10 | Chemokine CxC family |
| CXCL11 | C-X-C motif chemokine 11 | Chemokine CxC family |
| CXCL12 | Stromal cell-derived factor 1 | Chemokine CxC family, growth factor |
| CXCL13 | C-X-C motif chemokine 13 | Chemokine CxC family |
| CX3CL1 | Fractalkine | Intercrine delta family |
|  |  |  |
| **Inflammatory cytokines** | |  |
| ***Interleukins*** |  |  |
| IL1 alpha | Interleukin-1 alpha | IL1 family, mitogen |
| IL2 | Interleukin-2 | IL2 family, growth factor |
| IL4 | Interleukin-4 | IL4/IL13 family, growth factor |
| IL5 | Interleukin-5 | IL5 family, growth factor |
| **Label, group** | **Name** | **Protein type** |
| IL6 | Interleukin-6 | IL6 superfamily, growth factor |
| IL7 | Interleukin-7 | IL7/IL9 family, growth factor |
| IL8 | Interleukin-8 | Chemokine CxC family |
| IL10 | Interleukin-10 | IL10 family |
| IL12 | Interleukin-12 | IL6 superfamily, growth factor |
| IL12RB1 | Interleukin-12 receptor subunit beta-1 | Type I cytokine receptor family, cytokine receptor |
| IL13 | Interleukin-13 | IL4/IL13 family |
| IL18 | Interleukin-18 | IL1 family |
| IL21 | Interleukin-21 | IL15/IL21 family, growth factor |
| IL33 | Interleukin-33 | IL1 family |
| IL35 | Interleukin-35 | IL12 family |
| ***Interferon*** |  |  |
| IFN-beta | Interferon beta | Alpha/beta interferon family |
| IFN-gamma | Interferon gamma | Gamma interferon family |
| ***TNF(R) superfamily*** | |  |
| TNF | Tumor necrosis factor | TNF superfamily |
| TNFSF14 | Tumor necrosis factor ligand superfamily member 14 | TNF superfamily |
| TRAIL | TNF-related apoptosis-inducing ligand | TNF superfamily |
| TWEAK | Tumor necrosis factor (ligand) superfamily, member 12 | TNF superfamily |
| TNFRSF4 | Tumor necrosis factor receptor superfamily member 4 | TNFR superfamily |
| TNFRSF9 | Tumor necrosis factor receptor superfamily member 9 | TNFR superfamily |
| TNFRSF12A | Tumor necrosis factor receptor superfamily member 12A | TNFR superfamily |
| TNFRSF21 | Tumor necrosis factor receptor superfamily member 21 | TNFR superfamily |
| CD27 | CD27 antigen | TNFR superfamily |
| CD40 | CD40 ligand receptor | TNFR superfamily |
| CD40L | CD40 ligand | TNF superfamily |
| CD70 | CD70 antigen | TNF superfamily |
| **Label, group** | **Name** | **Protein type** |
| FasLG | Fas antigen ligand | TNF superfamily |
|  |  |  |
| **Growth factors (receptors)** | |  |
| ANG1 | Angiopoietin-1 | Growth factor, VEGF family |
| ANGPT2 | Angiopoietin-2 | Growth factor |
| CSF1 | Macrophage colony-stimulating factor 1 | Growth factor, cytokine |
| EGF | Pro-epidermal growth factor | Growth factor |
| FGF2 | Fibroblast growth factor 2 | Growth factor |
| HGF | Hepatocyte growth factor | Growth factor |
| LAP TGF-beta-1 | Latency-associated peptide transforming growth factor beta-1 | Growth factor, TGF beta superfamily |
| PDGF subunit B | Platelet-derived growth factor subunit B | Growth factor, PDGF/VEGF family |
| PGF | Placenta growth factor | Growth factor, PDGF/VEGF family |
| PTN | Pleiotrophin | Growth factor, pleiotrophin |
| VEGFA | Vascular endothelial growth factor A | Growth factor, VEGF family |
| VEGFC | Vascular endothelial growth factor C | Growth factor, VEGF family |
| VEGFR2 | Vascular endothelial growth factor receptor 2 | Growth factor, PDGF/VEGF receptor family |
|  |  |  |
| **Other proteins** |  |  |
| ***Enzymes*** |  |  |
| ADA | Adenosine deaminase | Purine metabolism, breaking down adenosine into nucleic acids |
| ARG1 | Arginase-1 | Urea metabolism, converting L-arginine to urea and L-ornithine |
| CAIX | Carbonic anhydrase IX | Reversible hydration of carbon dioxide |
| CASP8 | Caspase-8 | Cysteine protease, activating the TNFRSF6/FAS mediated and TNFRSF1A induced cell death |
| GZMA | Granzyme A | Protease, involved in granzyme-mediated apoptotic signaling pathway |
| GZMB | Granzyme B | Protease, involved in granzyme-mediated apoptotic signaling pathway |
| GZMH | Granzyme H | Protease, involved in granzyme-mediated apoptotic signaling pathway |
| **Label, group** | **Name** | **Protein type** |
| HO1 | Heme oxygenase 1 | Heme catabolism, cleaving the heme ring to form biliverdin |
| MMP7 | Matrix metalloproteinase-7 | Protease, activating procollagenase |
| MMP12 | Matrix metalloproteinase-12 | Protease, with elastolytic activity |
| NOS3 | Nitric oxide synthase, endothelial | Mediating VEGF-induced angiogenesis |
| ***Membrane proteins*** | |  |
| CD4 | T-cell surface glycoprotein CD4 | T-cell surface glycoprotein (adaptive) |
| CD5 | T-cell surface glycoprotein CD5 | T-cell surface glycoprotein (adaptive) |
| CD8A | T-cell surface glycoprotein CD8 alpha chain | T-cell surface glycoprotein (adaptive) |
| CD28 | T-cell surface glycoprotein CD28 | T-cell surface glycoprotein (adaptive) |
| CD83 | CD83 antigen | Dendritic cell surface protein (innate) |
| CD244 | Natural killer cell receptor 2B4 | Glycoprotein receptor on NK, monocytes, basophils (innate) |
| CRTAM | Cytotoxic and regulatory T-cell molecule | T-cell molecule (innate and adaptive) |
| ICOSLG | ICOS ligand | T-cell surface receptor ICOS (adaptive) |
| KLRD1 | Natural killer cells antigen CD94 | Receptor on NK cells and some cytotoxic T-cells (for MHC class MHC class I HLA-E molecules) (innate and adpative) |
| LAMP3 | Lysosome-associated membrane glycoprotein 3 | Lysosome membrane protein (adaptive immunity) |
| NCR1 | Natural cytotoxicity triggering receptor 1 | Cell surface receptor (innate immunity) |
| MIC-A/B | MHC class I polypeptide-related sequence A/B | MHC class I, expressed on the cell surface of all nucleated cells and also platelets (widely expressed) |
| ADGRG1 | Adhesion G-protein coupled receptor G1 | Receptor expressed in liver, muscle, neural, cytotoxic lymphoid cells (widely expressed) |
| TIE2 | Angiopoietin-1 receptor | Enzyme/ cell surface protein (widely expressed) |
| PD-L1 | Programmed cell death 1 ligand 1 | Ligand of the inhibitory receptor PD-1 (adaptive immunity) |
| PD-L2 | Programmed cell death 1 ligand 2 | Ligand of the inhibitory receptor PD-2 (adaptive immunity) |
| PDCD1 | Programmed cell death protein 1 | Cell surface receptor (adaptive immunity) |
| ***Extracellular proteins*** | |  |
| Gal-1 | Galectin-1 | Galectin |
| Gal-9 | Galectin-9 | Galectin |
| DCN | Decorin | Proteoglycan |

#

# Table S3. Limit of detection (LOD) of 92 proteins

| **Protein** | **LOD** |  |  | **Protein** | **LOD** |
| --- | --- | --- | --- | --- | --- |
| ADA | 0.267 |  |  | IL2 | 0.923 |
| ADGRG1 | 1.152 |  |  | IL4 | 0.995 |
| ANG1 | 0.8 |  |  | IL5 | 2.199 |
| ANGPT2 | 0.62 |  |  | IL6 | 1.118 |
| ARG1 | 1.591 |  |  | IL7 | 0.924 |
| CAIX | 0.149 |  |  | IL8 | 1.028 |
| CASP8 | 1.299 |  |  | IL10 | 1.465 |
| CCL3 | 0.925 |  |  | IL12 | 1.296 |
| CCL4 | 0.645 |  |  | IL12RB1 | 1.101 |
| CCL17 | 0.483 |  |  | IL13 | 1.429 |
| CCL19 | 1.101 |  |  | IL18 | 1.64 |
| CCL20 | 1.217 |  |  | IL21 | 1.437 |
| CCL23 | 0.338 |  |  | IL33 | 0.93 |
| CD4 | -1.608 |  |  | IL35 | 2.463 |
| CD5 | -0.071 |  |  | KLRD1 | 0.721 |
| CD8A | 0.776 |  |  | LAMP3 | 1.043 |
| CD27 | 0.391 |  |  | LAP TGF-beta-1 | -0.712 |
| CD28 | 0.538 |  |  | MCP1 | 0.662 |
| CD40 | 0.807 |  |  | MCP2 | 0.897 |
| CD40L | 1.506 |  |  | MCP3 | 1.219 |
| CD70 | 1.065 |  |  | MCP4 | 0.125 |
| CD83 | 0.11 |  |  | MIC-A/B | 0.545 |
| CD244 | 1.476 |  |  | MMP12 | 0.48 |
| CRTAM | 0.749 |  |  | MMP7 | 1.556 |
| CSF1 | 1.012 |  |  | NCR1 | 1.702 |
| CX3CL1 | 1.044 |  |  | NOS3 | 1.383 |
| CXCL1 | 0.986 |  |  | PD L1 | 1.804 |
| CXCL5 | 1.564 |  |  | PD L2 | 0.728 |
| CXCL9 | 0.721 |  |  | PDCD1 | 1.426 |
| CXCL10 | 0.877 |  |  | PDGF subunit B | 0.164 |
| CXCL11 | 0.785 |  |  | PGF | 0.698 |
| CXCL12 | 0.92 |  |  | PTN | 0.078 |
| CXCL13 | 1.379 |  |  | TIE2 | 0.553 |
| DCN | 0.366 |  |  | TNF | 1.804 |
| EGF | 0.289 |  |  | TNFRSF4 | 1.545 |
| FasL | 0.479 |  |  | TNFRSF9 | -0.385 |
| FGF2 | 0.479 |  |  | TNFRSF12A | 0.503 |
| Gal1 | 1.537 |  |  | TNFRSF21 | 0.136 |
| Gal9 | 0.606 |  |  | TNFSF14 | 0.306 |
| GZMA | 0.991 |  |  | TRAIL | 0.156 |
| GZMB | 0.758 |  |  | TWEAK | -0.421 |
| GZMH | 1.6 |  |  | VEGFA | 0.762 |
| HGF | 0.608 |  |  | VEGFC | -0.284 |
| HO1 | 1.431 |  |  | VEGFR2 | 0.068 |
| ICOSLG | 0.901 |  |  |  |  |
| IFN beta | 0.442 |  |  |  |  |
| IFN gamma | 0.76 |  |  |  |  |
| IL1 alpha | 0.423 |  |  |  |  |

LOD: limit of detection, normalised protein expression

# Table S4. Hazard ratios (95% CIs) for all proteins (per SD higher NPX), adjusted for age, age^2^, sex, region, smoking, alcohol, education, diabetes, fasting time and stratified by region, with time in study as the time scale

#

|  | **logHR** | **HR** | **se(logHR)** | **95% CI** | **z** | **Pr(>\|z\|)** | **p_adj_** |
| --- | --- | --- | --- | --- | --- | --- | --- |
| MCP3/CCL7 | 0.2542 | 1.29 | 0.08 | (1.10, 1.51) | 3.193 | 1.40E-03 | 4.70E-02 |
| ANGPT2 | 0.2413 | 1.27 | 0.076 | (1.10, 1.48) | 3.167 | 1.50E-03 | 4.70E-02 |
| IL18 | 0.2124 | 1.24 | 0.075 | (1.07, 1.43) | 2.831 | 4.60E-03 | 8.80E-02 |
| IL6 | 0.1894 | 1.21 | 0.067 | (1.06, 1.38) | 2.817 | 4.80E-03 | 8.80E-02 |
| LAMP3 | 0.2307 | 1.26 | 0.088 | (1.06, 1.50) | 2.624 | 8.70E-03 | 1.30E-01 |
| CCL3 | 0.1669 | 1.18 | 0.067 | (1.04, 1.35) | 2.477 | 1.30E-02 | 1.50E-01 |
| CD4 | 0.1631 | 1.18 | 0.067 | (1.03, 1.34) | 2.446 | 1.40E-02 | 1.50E-01 |
| CD8A | 0.1883 | 1.21 | 0.077 | (1.04, 1.40) | 2.438 | 1.50E-02 | 1.50E-01 |
| HO1 | 0.1817 | 1.2 | 0.079 | (1.03, 1.40) | 2.293 | 2.20E-02 | 1.90E-01 |
| HGF | 0.1892 | 1.21 | 0.083 | (1.03, 1.42) | 2.28 | 2.30E-02 | 1.90E-01 |
| IL2 | 0.6207 | 1.86 | 0.277 | (1.08, 3.20) | 2.237 | 2.50E-02 | 1.90E-01 |
| IL4 | -0.1566 | 0.86 | 0.074 | (0.74, 0.99) | -2.108 | 3.50E-02 | 2.40E-01 |
| GZMA | 0.183 | 1.2 | 0.087 | (1.01, 1.43) | 2.093 | 3.60E-02 | 2.40E-01 |
| CRTAM | 0.1691 | 1.18 | 0.086 | (1.00, 1.40) | 1.968 | 4.90E-02 | 2.80E-01 |
| ADGRG1 | 0.1436 | 1.15 | 0.073 | (1.00, 1.33) | 1.967 | 4.90E-02 | 2.80E-01 |
| ARG1 | -0.1494 | 0.86 | 0.078 | (0.74, 1.00) | -1.922 | 5.50E-02 | 2.80E-01 |
| Gal-1 | 0.1586 | 1.17 | 0.083 | (1.00, 1.38) | 1.906 | 5.70E-02 | 2.80E-01 |
| CD40 | 0.147 | 1.16 | 0.078 | (0.99, 1.35) | 1.873 | 6.10E-02 | 2.80E-01 |
| IL5 | -0.1542 | 0.86 | 0.083 | (0.73, 1.01) | -1.859 | 6.30E-02 | 2.80E-01 |
| CXCL13 | 0.1503 | 1.16 | 0.081 | (0.99, 1.36) | 1.846 | 6.50E-02 | 2.80E-01 |
| CCL23 | 0.136 | 1.15 | 0.075 | (0.99, 1.33) | 1.825 | 6.80E-02 | 2.80E-01 |
| MIC-A/B | -0.1337 | 0.87 | 0.077 | (0.75, 1.02) | -1.746 | 8.10E-02 | 3.00E-01 |
| CCL17 | 0.147 | 1.16 | 0.084 | (0.98, 1.37) | 1.745 | 8.10E-02 | 3.00E-01 |
| TNFRSF9 | 0.1389 | 1.15 | 0.08 | (0.98, 1.34) | 1.734 | 8.30E-02 | 3.00E-01 |
| IFN-beta | -0.0901 | 0.91 | 0.055 | (0.82, 1.02) | -1.641 | 1.00E-01 | 3.50E-01 |
| CCL19 | 0.1214 | 1.13 | 0.075 | (0.97, 1.31) | 1.611 | 1.10E-01 | 3.60E-01 |
| MMP7 | 0.1373 | 1.15 | 0.088 | (0.97, 1.36) | 1.565 | 1.20E-01 | 3.80E-01 |
| TIE2 | 0.1174 | 1.12 | 0.076 | (0.97, 1.30) | 1.547 | 1.20E-01 | 3.80E-01 |
| CD83 | 0.1151 | 1.12 | 0.08 | (0.96, 1.31) | 1.446 | 1.50E-01 | 4.40E-01 |
| PDCD1 | 0.0834 | 1.09 | 0.058 | (0.97, 1.22) | 1.439 | 1.50E-01 | 4.40E-01 |
| GZMB | 0.1006 | 1.11 | 0.071 | (0.96, 1.27) | 1.415 | 1.60E-01 | 4.50E-01 |
| TNFRSF12A | 0.1099 | 1.12 | 0.081 | (0.95, 1.31) | 1.358 | 1.70E-01 | 4.70E-01 |
| CCL4 | 0.0959 | 1.1 | 0.071 | (0.96, 1.26) | 1.358 | 1.70E-01 | 4.70E-01 |
| ADA | 0.0905 | 1.09 | 0.074 | (0.95, 1.27) | 1.221 | 2.20E-01 | 5.80E-01 |
| TNFSF14 | 0.0833 | 1.09 | 0.071 | (0.95, 1.25) | 1.176 | 2.40E-01 | 6.10E-01 |
| CASP8 | -0.0816 | 0.92 | 0.072 | (0.80, 1.06) | -1.14 | 2.50E-01 | 6.10E-01 |
| VEGFA | 0.0877 | 1.09 | 0.078 | (0.94, 1.27) | 1.131 | 2.60E-01 | 6.10E-01 |
| CD70 | 0.0938 | 1.1 | 0.083 | (0.93, 1.29) | 1.124 | 2.60E-01 | 6.10E-01 |
| CD5 | 0.0871 | 1.09 | 0.079 | (0.93, 1.27) | 1.097 | 2.70E-01 | 6.20E-01 |
| MMP12 | 0.0931 | 1.1 | 0.086 | (0.93, 1.30) | 1.084 | 2.80E-01 | 6.20E-01 |
| TNF | 0.0633 | 1.07 | 0.063 | (0.94, 1.21) | 0.997 | 3.20E-01 | 6.80E-01 |
| LAP TGF-beta-1 | 0.0749 | 1.08 | 0.076 | (0.93, 1.25) | 0.989 | 3.20E-01 | 6.80E-01 |
| NOS3 | 0.0722 | 1.07 | 0.077 | (0.93, 1.25) | 0.943 | 3.50E-01 | 7.10E-01 |
|  | **logHR** | **HR** | **se(logHR)** | **95% CI** | **z** | **Pr(>\|z\|)** | **p_adj_** |
| CXCL10 | 0.0743 | 1.08 | 0.081 | (0.92, 1.26) | 0.914 | 3.60E-01 | 7.20E-01 |
| CXCL9 | 0.0711 | 1.07 | 0.08 | (0.92, 1.26) | 0.887 | 3.80E-01 | 7.20E-01 |
| ICOSLG | -0.0652 | 0.94 | 0.074 | (0.81, 1.08) | -0.885 | 3.80E-01 | 7.20E-01 |
| TNFRSF4 | 0.0686 | 1.07 | 0.078 | (0.92, 1.25) | 0.876 | 3.80E-01 | 7.20E-01 |
| Gal-9 | 0.0795 | 1.08 | 0.092 | (0.90, 1.30) | 0.862 | 3.90E-01 | 7.20E-01 |
| PGF | 0.0612 | 1.06 | 0.077 | (0.91, 1.24) | 0.799 | 4.20E-01 | 7.20E-01 |
| IL10 | 0.056 | 1.06 | 0.071 | (0.92, 1.22) | 0.784 | 4.30E-01 | 7.20E-01 |
| EGF | -0.0596 | 0.94 | 0.077 | (0.81, 1.10) | -0.771 | 4.40E-01 | 7.20E-01 |
| TWEAK | -0.0562 | 0.95 | 0.073 | (0.82, 1.09) | -0.769 | 4.40E-01 | 7.20E-01 |
| GZMH | 0.053 | 1.05 | 0.07 | (0.92, 1.21) | 0.76 | 4.50E-01 | 7.20E-01 |
| NCR1 | 0.0534 | 1.05 | 0.073 | (0.91, 1.22) | 0.728 | 4.70E-01 | 7.20E-01 |
| IL33 | -0.0477 | 0.95 | 0.066 | (0.84, 1.08) | -0.724 | 4.70E-01 | 7.20E-01 |
| CX3CL1 | 0.0567 | 1.06 | 0.078 | (0.91, 1.23) | 0.724 | 4.70E-01 | 7.20E-01 |
| MCP4 | -0.0602 | 0.94 | 0.084 | (0.80, 1.11) | -0.718 | 4.70E-01 | 7.20E-01 |
| MCP1/CCL2 | 0.0567 | 1.06 | 0.079 | (0.91, 1.24) | 0.718 | 4.70E-01 | 7.20E-01 |
| MCP2/CCL8 | -0.056 | 0.95 | 0.079 | (0.81, 1.10) | -0.711 | 4.80E-01 | 7.20E-01 |
| IL12RB1 | 0.0477 | 1.05 | 0.069 | (0.92, 1.20) | 0.689 | 4.90E-01 | 7.20E-01 |
| VEGFR2 | 0.0475 | 1.05 | 0.069 | (0.92, 1.20) | 0.685 | 4.90E-01 | 7.20E-01 |
| CD28 | 0.0524 | 1.05 | 0.08 | (0.90, 1.23) | 0.657 | 5.10E-01 | 7.20E-01 |
| CXCL1 | -0.0525 | 0.95 | 0.081 | (0.81, 1.11) | -0.652 | 5.10E-01 | 7.20E-01 |
| IL13 | 0.0353 | 1.04 | 0.054 | (0.93, 1.15) | 0.65 | 5.20E-01 | 7.20E-01 |
| KLRD1 | -0.0465 | 0.95 | 0.077 | (0.82, 1.11) | -0.6 | 5.50E-01 | 7.60E-01 |
| IL12 | 0.0447 | 1.05 | 0.08 | (0.89, 1.22) | 0.562 | 5.70E-01 | 7.80E-01 |
| CAIX | 0.0424 | 1.04 | 0.082 | (0.89, 1.22) | 0.519 | 6.00E-01 | 8.10E-01 |
| DCN | 0.0376 | 1.04 | 0.076 | (0.89, 1.21) | 0.495 | 6.20E-01 | 8.20E-01 |
| FasLG | -0.0349 | 0.97 | 0.079 | (0.83, 1.13) | -0.44 | 6.60E-01 | 8.20E-01 |
| CSF1 | 0.0308 | 1.03 | 0.072 | (0.90, 1.19) | 0.429 | 6.70E-01 | 8.20E-01 |
| PTN | 0.0396 | 1.04 | 0.094 | (0.86, 1.25) | 0.419 | 6.80E-01 | 8.20E-01 |
| CD244 | -0.0323 | 0.97 | 0.077 | (0.83, 1.13) | -0.418 | 6.80E-01 | 8.20E-01 |
| CD40L | 0.0319 | 1.03 | 0.077 | (0.89, 1.20) | 0.412 | 6.80E-01 | 8.20E-01 |
| IL7 | -0.0291 | 0.97 | 0.072 | (0.84, 1.12) | -0.404 | 6.90E-01 | 8.20E-01 |
| IL8 | -0.0283 | 0.97 | 0.071 | (0.85, 1.12) | -0.399 | 6.90E-01 | 8.20E-01 |
| ANG1 | -0.0329 | 0.97 | 0.084 | (0.82, 1.14) | -0.394 | 6.90E-01 | 8.20E-01 |
| CXCL11 | -0.0257 | 0.97 | 0.077 | (0.84, 1.13) | -0.336 | 7.40E-01 | 8.60E-01 |
| CD27 | 0.0225 | 1.02 | 0.081 | (0.87, 1.20) | 0.279 | 7.80E-01 | 9.00E-01 |
| PD-L2 | -0.0204 | 0.98 | 0.077 | (0.84, 1.14) | -0.264 | 7.90E-01 | 9.00E-01 |
| CXCL5 | -0.0199 | 0.98 | 0.084 | (0.83, 1.16) | -0.236 | 8.10E-01 | 9.10E-01 |
| VEGFC | -0.0166 | 0.98 | 0.073 | (0.85, 1.14) | -0.226 | 8.20E-01 | 9.10E-01 |
| PDGF subunit B | -0.0176 | 0.98 | 0.081 | (0.84, 1.15) | -0.217 | 8.30E-01 | 9.10E-01 |
| TNFRSF21 | 0.016 | 1.02 | 0.078 | (0.87, 1.18) | 0.205 | 8.40E-01 | 9.10E-01 |
| CCL20 | -0.014 | 0.99 | 0.073 | (0.86, 1.14) | -0.193 | 8.50E-01 | 9.10E-01 |
| FGF2 | -0.0107 | 0.99 | 0.068 | (0.87, 1.13) | -0.157 | 8.80E-01 | 9.30E-01 |
| PD-L1 | -0.0097 | 0.99 | 0.071 | (0.86, 1.14) | -0.136 | 8.90E-01 | 9.30E-01 |
| IL1 alpha | -0.0089 | 0.99 | 0.076 | (0.85, 1.15) | -0.117 | 9.10E-01 | 9.30E-01 |
| IFN-gamma | 0.0074 | 1.01 | 0.076 | (0.87, 1.17) | 0.097 | 9.20E-01 | 9.30E-01 |
| TRAIL | -0.0059 | 0.99 | 0.062 | (0.88, 1.12) | -0.095 | 9.20E-01 | 9.30E-01 |
| CXCL12 | 0.006 | 1.01 | 0.082 | (0.86, 1.18) | 0.073 | 9.40E-01 | 9.40E-01 |

# Table S5. Hazard ratios (95% CIs) for all proteins (per SD higher NPX), adjusted for age and sex and stratified by region, with time in study as the time scale

|  | **logHR** | **HR** | **se(logHR)** | **95% CI** | **z** | **Pr(>\|z\|)** | **p_adj_** |
| --- | --- | --- | --- | --- | --- | --- | --- |
| MCP3/CCL7 | 0.2714 | 1.31 | 0.0761 | (1.13, 1.52) | 3.566 | 0.00036 | 0.013 |
| ANGPT2 | 0.2542 | 1.29 | 0.072 | (1.12, 1.48) | 3.53 | 0.00042 | 0.013 |
| IL18 | 0.2229 | 1.25 | 0.0731 | (1.08, 1.44) | 3.047 | 0.00231 | 0.053 |
| LAMP3 | 0.2295 | 1.26 | 0.0778 | (1.08, 1.47) | 2.949 | 0.00319 | 0.056 |
| IL6 | 0.1784 | 1.2 | 0.0615 | (1.06, 1.35) | 2.902 | 0.00371 | 0.056 |
| CCL3 | 0.1723 | 1.19 | 0.0655 | (1.04, 1.35) | 2.63 | 0.00853 | 0.099 |
| IL2 | 0.5879 | 1.8 | 0.2262 | (1.16, 2.80) | 2.599 | 0.00934 | 0.099 |
| CD8A | 0.193 | 1.21 | 0.0747 | (1.05, 1.40) | 2.583 | 0.00979 | 0.099 |
| HO1 | 0.1878 | 1.21 | 0.0776 | (1.04, 1.40) | 2.42 | 0.01552 | 0.128 |
| CD4 | 0.1567 | 1.17 | 0.065 | (1.03, 1.33) | 2.412 | 0.01587 | 0.128 |
| HGF | 0.1946 | 1.21 | 0.0815 | (1.04, 1.43) | 2.389 | 0.0169 | 0.128 |
| CD40 | 0.1725 | 1.19 | 0.0744 | (1.03, 1.37) | 2.32 | 0.02036 | 0.143 |
| IL5 | -0.1839 | 0.83 | 0.0859 | (0.70, 0.98) | -2.141 | 0.03231 | 0.21 |
| TNFRSF9 | 0.1554 | 1.17 | 0.0743 | (1.01, 1.35) | 2.092 | 0.03648 | 0.221 |
| GZMA | 0.1719 | 1.19 | 0.0841 | (1.01, 1.40) | 2.043 | 0.04102 | 0.225 |
| ADGRG1 | 0.1444 | 1.16 | 0.0718 | (1.00, 1.33) | 2.01 | 0.04443 | 0.225 |
| TIE2 | 0.1475 | 1.16 | 0.0734 | (1.00, 1.34) | 2.01 | 0.04445 | 0.225 |
| IL4 | -0.1398 | 0.87 | 0.0713 | (0.76, 1.00) | -1.961 | 0.04989 | 0.239 |
| CCL17 | 0.1544 | 1.17 | 0.0818 | (0.99, 1.37) | 1.888 | 0.05899 | 0.267 |
| CXCL13 | 0.1494 | 1.16 | 0.0799 | (0.99, 1.36) | 1.869 | 0.06164 | 0.267 |
| Gal-1 | 0.1443 | 1.16 | 0.0789 | (0.99, 1.35) | 1.83 | 0.06732 | 0.278 |
| CD83 | 0.133 | 1.14 | 0.0742 | (0.99, 1.32) | 1.793 | 0.07305 | 0.287 |
| CRTAM | 0.144 | 1.15 | 0.0816 | (0.98, 1.36) | 1.765 | 0.07764 | 0.287 |
| ARG1 | -0.1308 | 0.88 | 0.0748 | (0.76, 1.02) | -1.748 | 0.08046 | 0.287 |
| MIC-A/B | -0.1297 | 0.88 | 0.0746 | (0.76, 1.02) | -1.739 | 0.08201 | 0.287 |
| MMP7 | 0.1394 | 1.15 | 0.0854 | (0.97, 1.36) | 1.632 | 0.10271 | 0.336 |
| IFN-beta | -0.0871 | 0.92 | 0.0535 | (0.83, 1.02) | -1.629 | 0.10332 | 0.336 |
| TNFRSF12A | 0.12 | 1.13 | 0.0752 | (0.97, 1.31) | 1.595 | 0.11073 | 0.34 |
| LAP TGF-beta-1 | 0.1113 | 1.12 | 0.0707 | (0.97, 1.28) | 1.575 | 0.11526 | 0.34 |
| CCL19 | 0.1142 | 1.12 | 0.0726 | (0.97, 1.29) | 1.572 | 0.11588 | 0.34 |
| MMP12 | 0.1245 | 1.13 | 0.0825 | (0.96, 1.33) | 1.508 | 0.1316 | 0.374 |
| CCL4 | 0.1008 | 1.11 | 0.0706 | (0.96, 1.27) | 1.428 | 0.1533 | 0.401 |
| CCL23 | 0.1029 | 1.11 | 0.0722 | (0.96, 1.28) | 1.426 | 0.1538 | 0.401 |
| TNFSF14 | 0.0985 | 1.1 | 0.0691 | (0.96, 1.26) | 1.424 | 0.15434 | 0.401 |
| ADA | 0.1008 | 1.11 | 0.0743 | (0.96, 1.28) | 1.357 | 0.17476 | 0.432 |
| CAIX | 0.1003 | 1.11 | 0.074 | (0.96, 1.28) | 1.354 | 0.17573 | 0.432 |
| VEGFA | 0.098 | 1.1 | 0.0738 | (0.95, 1.27) | 1.328 | 0.18434 | 0.439 |
| GZMB | 0.0933 | 1.1 | 0.0709 | (0.96, 1.26) | 1.316 | 0.18831 | 0.439 |
| PDCD1 | 0.0729 | 1.08 | 0.0571 | (0.96, 1.20) | 1.278 | 0.20122 | 0.458 |
| MCP1/CCL2 | 0.0888 | 1.09 | 0.0763 | (0.94, 1.27) | 1.163 | 0.24477 | 0.543 |
| VEGFR2 | 0.0757 | 1.08 | 0.0668 | (0.95, 1.23) | 1.134 | 0.25693 | 0.557 |
| Gal-9 | 0.0987 | 1.1 | 0.0891 | (0.93, 1.31) | 1.108 | 0.26771 | 0.567 |
| IL10 | 0.0708 | 1.07 | 0.0697 | (0.94, 1.23) | 1.016 | 0.30982 | 0.626 |
|  | **logHR** | **HR** | **se(logHR)** | **95% CI** | **z** | **Pr(>\|z\|)** | **p_adj_** |
| ICOSLG | -0.0746 | 0.93 | 0.0736 | (0.80, 1.07) | -1.014 | 0.31077 | 0.626 |
| CASP8 | -0.0701 | 0.93 | 0.07 | (0.81, 1.07) | -1.002 | 0.31629 | 0.626 |
| TNFRSF4 | 0.0754 | 1.08 | 0.0763 | (0.93, 1.25) | 0.988 | 0.32307 | 0.626 |
| CD70 | 0.0786 | 1.08 | 0.0829 | (0.92, 1.27) | 0.947 | 0.3434 | 0.629 |
| TNF | 0.0585 | 1.06 | 0.062 | (0.94, 1.20) | 0.944 | 0.34539 | 0.629 |
| CD40L | 0.069 | 1.07 | 0.0733 | (0.93, 1.24) | 0.942 | 0.34628 | 0.629 |
| CD5 | 0.0716 | 1.07 | 0.077 | (0.92, 1.25) | 0.93 | 0.35236 | 0.629 |
| CX3CL1 | 0.0615 | 1.06 | 0.0745 | (0.92, 1.23) | 0.825 | 0.4096 | 0.717 |
| PGF | 0.0563 | 1.06 | 0.0734 | (0.92, 1.22) | 0.766 | 0.44357 | 0.762 |
| CXCL9 | 0.0576 | 1.06 | 0.0771 | (0.91, 1.23) | 0.747 | 0.45513 | 0.767 |
| IL33 | -0.0451 | 0.96 | 0.0648 | (0.84, 1.09) | -0.697 | 0.48601 | 0.788 |
| CD28 | 0.0548 | 1.06 | 0.0804 | (0.90, 1.24) | 0.682 | 0.49523 | 0.788 |
| NOS3 | 0.0497 | 1.05 | 0.0739 | (0.91, 1.21) | 0.672 | 0.50147 | 0.788 |
| CXCL10 | 0.0527 | 1.05 | 0.0786 | (0.90, 1.23) | 0.671 | 0.50234 | 0.788 |
| NCR1 | 0.0457 | 1.05 | 0.0702 | (0.91, 1.20) | 0.65 | 0.51539 | 0.788 |
| TWEAK | -0.0461 | 0.95 | 0.0715 | (0.83, 1.10) | -0.644 | 0.51967 | 0.788 |
| CSF1 | 0.0419 | 1.04 | 0.0702 | (0.91, 1.20) | 0.598 | 0.54997 | 0.805 |
| GZMH | 0.0403 | 1.04 | 0.0691 | (0.91, 1.19) | 0.583 | 0.55966 | 0.805 |
| CD27 | 0.0465 | 1.05 | 0.08 | (0.90, 1.23) | 0.581 | 0.56106 | 0.805 |
| FasLG | -0.0442 | 0.96 | 0.077 | (0.82, 1.11) | -0.574 | 0.56595 | 0.805 |
| DCN | 0.0386 | 1.04 | 0.0747 | (0.90, 1.20) | 0.517 | 0.6055 | 0.848 |
| IL13 | 0.0288 | 1.03 | 0.0574 | (0.92, 1.15) | 0.501 | 0.61651 | 0.85 |
| IL12 | 0.0355 | 1.04 | 0.0778 | (0.89, 1.21) | 0.456 | 0.64834 | 0.866 |
| IL12RB1 | 0.0315 | 1.03 | 0.0692 | (0.90, 1.18) | 0.455 | 0.64879 | 0.866 |
| CXCL5 | 0.0357 | 1.04 | 0.0802 | (0.89, 1.21) | 0.445 | 0.65645 | 0.866 |
| MCP2/CCL8 | -0.0338 | 0.97 | 0.0795 | (0.83, 1.13) | -0.425 | 0.67054 | 0.872 |
| IL8 | 0.0265 | 1.03 | 0.0667 | (0.90, 1.17) | 0.398 | 0.69086 | 0.879 |
| PDGF subunit B | 0.0303 | 1.03 | 0.0773 | (0.89, 1.20) | 0.392 | 0.69537 | 0.879 |
| ANG1 | 0.0277 | 1.03 | 0.0789 | (0.88, 1.20) | 0.351 | 0.72579 | 0.905 |
| CCL20 | 0.0188 | 1.02 | 0.0689 | (0.89, 1.17) | 0.273 | 0.78476 | 0.965 |
| KLRD1 | -0.0188 | 0.98 | 0.0734 | (0.85, 1.13) | -0.256 | 0.79766 | 0.968 |
| EGF | -0.0148 | 0.99 | 0.0736 | (0.85, 1.14) | -0.201 | 0.84046 | 0.985 |
| CD244 | -0.0139 | 0.99 | 0.0756 | (0.85, 1.14) | -0.184 | 0.85438 | 0.985 |
| VEGFC | 0.0112 | 1.01 | 0.0699 | (0.88, 1.16) | 0.16 | 0.87292 | 0.985 |
| MCP4 | -0.0122 | 0.99 | 0.0792 | (0.85, 1.15) | -0.153 | 0.87807 | 0.985 |
| IFN-gamma | -0.0083 | 0.99 | 0.0768 | (0.85, 1.15) | -0.108 | 0.91395 | 0.985 |
| FGF2 | 0.0073 | 1.01 | 0.0685 | (0.88, 1.15) | 0.107 | 0.91479 | 0.985 |
| CXCL11 | -0.0069 | 0.99 | 0.0739 | (0.86, 1.15) | -0.093 | 0.92584 | 0.985 |
| PTN | -0.009 | 0.99 | 0.0969 | (0.82, 1.20) | -0.092 | 0.92635 | 0.985 |
| IL1 alpha | 0.0066 | 1.01 | 0.0725 | (0.87, 1.16) | 0.091 | 0.92773 | 0.985 |
| CXCL1 | -0.0066 | 0.99 | 0.0774 | (0.85, 1.16) | -0.086 | 0.93165 | 0.985 |
| TNFRSF21 | 0.006 | 1.01 | 0.0764 | (0.87, 1.17) | 0.079 | 0.93708 | 0.985 |
| CXCL12 | 0.0036 | 1 | 0.0772 | (0.86, 1.17) | 0.046 | 0.96321 | 0.985 |
| TRAIL | 0.0018 | 1 | 0.0619 | (0.89, 1.13) | 0.029 | 0.97684 | 0.985 |
| PD-L1 | -0.0018 | 1 | 0.0674 | (0.87, 1.14) | -0.027 | 0.97856 | 0.985 |
| IL7 | -0.0017 | 1 | 0.0681 | (0.87, 1.14) | -0.025 | 0.98003 | 0.985 |
| PD-L2 | -0.0014 | 1 | 0.0746 | (0.86, 1.16) | -0.018 | 0.9853 | 0.985 |

# Table S6. Hazard ratios (95% CIs) for all proteins (per SD higher NPX) and their interaction with time since sample collection, adjusted for age, age^2^, sex, region, smoking, alcohol drinking, education, diabetes and time since last meal, with time in study as the time scale

|  | **logHR** | **HR** | **se(logHR)** | **z** | **Pr(>\|z\|)** |
| --- | --- | --- | --- | --- | --- |
| IL8 | 0.06 | 1.06 | 0.07 | 0.89 | 0.37 |
| IL8 log(t + 0.01) | -0.08 | 0.92 | 0.04 | -2.28 | 0.02 |
| TNFRSF9 | 0.24 | 1.27 | 0.1 | 2.36 | 0.02 |
| TNFRSF9 log(t + 0.01) | -0.08 | 0.93 | 0.04 | -1.93 | 0.05 |
| TIE2 | 0.15 | 1.17 | 0.1 | 1.51 | 0.13 |
| TIE2 log(t + 0.01) | -0.02 | 0.98 | 0.05 | -0.5 | 0.62 |
| MCP3/CCL7 | 0.34 | 1.4 | 0.1 | 3.34 | 0 |
| MCP3/CCL7 log(t + 0.01) | -0.05 | 0.95 | 0.05 | -1.03 | 0.3 |
| CD40L | 0.05 | 1.05 | 0.11 | 0.46 | 0.64 |
| CD40L log(t + 0.01) | -0.02 | 0.99 | 0.05 | -0.31 | 0.76 |
| IL1 alpha | -1.02 | 0.36 | 0.41 | -2.46 | 0.01 |
| IL1 alpha log(t + 0.01) | 0.54 | 1.72 | 0.19 | 2.91 | 0 |
| CD244 | 0.02 | 1.03 | 0.11 | 0.23 | 0.82 |
| CD244 log(t + 0.01) | -0.04 | 0.96 | 0.05 | -0.85 | 0.4 |
| EGF | -0.14 | 0.87 | 0.11 | -1.24 | 0.21 |
| EGF log(t + 0.01) | 0.05 | 1.05 | 0.05 | 0.96 | 0.34 |
| ANG1 | -0.06 | 0.94 | 0.11 | -0.55 | 0.58 |
| ANG1 log(t + 0.01) | 0.02 | 1.02 | 0.05 | 0.33 | 0.74 |
| IL7 | -0.16 | 0.85 | 0.11 | -1.52 | 0.13 |
| IL7 log(t + 0.01) | 0.09 | 1.09 | 0.05 | 1.66 | 0.1 |
| PGF | 0.15 | 1.16 | 0.1 | 1.51 | 0.13 |
| PGF log(t + 0.01) | -0.07 | 0.94 | 0.05 | -1.47 | 0.14 |
| IL6 | 0.33 | 1.39 | 0.08 | 4.18 | 0 |
| IL6 log(t + 0.01) | -0.09 | 0.91 | 0.03 | -2.87 | 0 |
| ADGRG1 | 0.25 | 1.28 | 0.08 | 3 | 0 |
| ADGRG1 log(t + 0.01) | -0.07 | 0.93 | 0.04 | -1.81 | 0.07 |
| MCP1/CCL2 | 0.09 | 1.09 | 0.12 | 0.74 | 0.46 |
| MCP1/CCL2 log(t + 0.01) | -0.02 | 0.98 | 0.06 | -0.33 | 0.74 |
| CRTAM | 0.26 | 1.3 | 0.11 | 2.44 | 0.01 |
| CRTAM log(t + 0.01) | -0.07 | 0.93 | 0.05 | -1.37 | 0.17 |
| CXCL11 | -0.03 | 0.97 | 0.11 | -0.27 | 0.79 |
| CXCL11 log(t + 0.01) | 0 | 1 | 0.05 | -0.01 | 0.99 |
| MCP4 | -0.08 | 0.93 | 0.11 | -0.67 | 0.5 |
| MCP4 log(t + 0.01) | 0.01 | 1.01 | 0.05 | 0.14 | 0.89 |
| TRAIL | -0.12 | 0.89 | 0.08 | -1.45 | 0.15 |
| TRAIL log(t + 0.01) | 0.08 | 1.09 | 0.04 | 2.22 | 0.03 |
| FGF2 | 0.07 | 1.07 | 0.07 | 0.93 | 0.35 |
| FGF2 log(t + 0.01) | -0.07 | 0.93 | 0.04 | -2.05 | 0.04 |
| CXCL9 | 0.15 | 1.16 | 0.11 | 1.41 | 0.16 |
| CXCL9 log(t + 0.01) | -0.06 | 0.94 | 0.05 | -1.1 | 0.27 |
|  |  |  |  |  |  |
|  | **logHR** | **HR** | **se(logHR)** | **z** | **Pr(>\|z\|)** |
| CD8A | 0.33 | 1.39 | 0.11 | 3.04 | 0 |
| CD8A log(t + 0.01) | -0.1 | 0.91 | 0.05 | -2.01 | 0.04 |
| CAIX | 0.08 | 1.08 | 0.1 | 0.81 | 0.42 |
| CAIX log(t + 0.01) | -0.03 | 0.97 | 0.04 | -0.81 | 0.42 |
| IFN-beta | -0.07 | 0.93 | 0.07 | -0.98 | 0.32 |
| IFN-beta log(t + 0.01) | -0.02 | 0.98 | 0.02 | -0.61 | 0.54 |
| ADA | 0.11 | 1.12 | 0.11 | 1.04 | 0.3 |
| ADA log(t + 0.01) | -0.01 | 0.99 | 0.05 | -0.24 | 0.81 |
| CD4 | 0.22 | 1.24 | 0.1 | 2.18 | 0.03 |
| CD4 log(t + 0.01) | -0.04 | 0.96 | 0.05 | -0.77 | 0.44 |
| NOS3 | 0.11 | 1.11 | 0.1 | 1.05 | 0.3 |
| NOS3 log(t + 0.01) | -0.02 | 0.98 | 0.04 | -0.53 | 0.6 |
| IL2 | 1.1 | 3 | 0.27 | 3.99 | 0 |
| IL2 log(t + 0.01) | -0.49 | 0.62 | 0.14 | -3.38 | 0 |
| Gal-9 | 0.22 | 1.25 | 0.13 | 1.69 | 0.09 |
| Gal-9 log(t + 0.01) | -0.1 | 0.91 | 0.06 | -1.56 | 0.12 |
| VEGFR2 | -0.05 | 0.95 | 0.09 | -0.51 | 0.61 |
| VEGFR2 log(t + 0.01) | 0.07 | 1.07 | 0.04 | 1.66 | 0.1 |
| CD40 | 0.32 | 1.37 | 0.1 | 3.2 | 0 |
| CD40 log(t + 0.01) | -0.12 | 0.88 | 0.05 | -2.66 | 0.01 |
| IL18 | 0.27 | 1.31 | 0.1 | 2.81 | 0 |
| IL18 log(t + 0.01) | -0.04 | 0.96 | 0.04 | -0.9 | 0.37 |
| GZMH | 0.13 | 1.13 | 0.1 | 1.25 | 0.21 |
| GZMH log(t + 0.01) | -0.05 | 0.95 | 0.05 | -1.05 | 0.3 |
| VEGFC | -0.15 | 0.86 | 0.11 | -1.38 | 0.17 |
| VEGFC log(t + 0.01) | 0.09 | 1.09 | 0.05 | 1.69 | 0.09 |
| LAP TGF-beta-1 | 0.04 | 1.04 | 0.11 | 0.32 | 0.75 |
| LAP TGF-beta-1 log(t + 0.01) | 0.02 | 1.03 | 0.06 | 0.42 | 0.67 |
| CXCL1 | -0.13 | 0.88 | 0.11 | -1.19 | 0.23 |
| CXCL1 log(t + 0.01) | 0.05 | 1.05 | 0.05 | 0.95 | 0.34 |
| TNFSF14 | 0.11 | 1.12 | 0.09 | 1.2 | 0.23 |
| TNFSF14 log(t + 0.01) | -0.02 | 0.98 | 0.04 | -0.49 | 0.62 |
| IL33 | 0 | 1 | 0.06 | -0.07 | 0.94 |
| IL33 log(t + 0.01) | -0.04 | 0.96 | 0.02 | -2.81 | 0 |
| TWEAK | -0.13 | 0.88 | 0.1 | -1.27 | 0.2 |
| TWEAK log(t + 0.01) | 0.05 | 1.05 | 0.05 | 1.01 | 0.31 |
| PDGF subunit B | -0.06 | 0.94 | 0.11 | -0.54 | 0.59 |
| PDGF subunit B log(t + 0.01) | 0.03 | 1.03 | 0.05 | 0.53 | 0.59 |
| PDCD1 | 0.11 | 1.11 | 0.06 | 1.74 | 0.08 |
| PDCD1 log(t + 0.01) | -0.02 | 0.98 | 0.02 | -0.99 | 0.32 |
| FasLG | -0.1 | 0.9 | 0.11 | -0.91 | 0.36 |
| FasLG log(t + 0.01) | 0.05 | 1.05 | 0.05 | 0.87 | 0.38 |
| CD28 | 0.01 | 1.01 | 0.11 | 0.13 | 0.9 |
| CD28 log(t + 0.01) | 0.03 | 1.03 | 0.05 | 0.53 | 0.59 |
| CCL19 | 0.04 | 1.04 | 0.1 | 0.38 | 0.7 |
| CCL19 log(t + 0.01) | 0.06 | 1.06 | 0.05 | 1.31 | 0.19 |
|  | **logHR** | **HR** | **se(logHR)** | **z** | **Pr(>\|z\|)** |
| MCP2/CCL8 | -0.13 | 0.88 | 0.11 | -1.26 | 0.21 |
| MCP2/CCL8 log(t + 0.01) | 0.05 | 1.05 | 0.05 | 1.08 | 0.28 |
| CCL4 | 0.1 | 1.1 | 0.1 | 0.98 | 0.33 |
| CCL4 log(t + 0.01) | 0 | 1 | 0.05 | 0.06 | 0.95 |
| Gal-1 | 0.19 | 1.21 | 0.12 | 1.64 | 0.1 |
| Gal-1 log(t + 0.01) | -0.02 | 0.98 | 0.05 | -0.41 | 0.68 |
| PD-L1 | 0.04 | 1.04 | 0.08 | 0.51 | 0.61 |
| PD-L1 log(t + 0.01) | -0.04 | 0.96 | 0.04 | -1.14 | 0.25 |
| CD27 | 0.2 | 1.22 | 0.11 | 1.72 | 0.08 |
| CD27 log(t + 0.01) | -0.13 | 0.88 | 0.05 | -2.36 | 0.02 |
| CXCL5 | -0.11 | 0.9 | 0.12 | -0.93 | 0.35 |
| CXCL5 log(t + 0.01) | 0.06 | 1.06 | 0.05 | 1.07 | 0.29 |
| IL5 | -0.27 | 0.77 | 0.16 | -1.72 | 0.09 |
| IL5 log(t + 0.01) | 0.07 | 1.08 | 0.08 | 0.94 | 0.35 |
| HGF | 0.43 | 1.54 | 0.12 | 3.6 | 0 |
| HGF log(t + 0.01) | -0.17 | 0.85 | 0.06 | -2.99 | 0 |
| GZMA | 0.23 | 1.25 | 0.11 | 2.08 | 0.04 |
| GZMA log(t + 0.01) | -0.03 | 0.97 | 0.05 | -0.58 | 0.56 |
| HO1 | 0.14 | 1.15 | 0.13 | 1.15 | 0.25 |
| HO1 log(t + 0.01) | 0.03 | 1.03 | 0.07 | 0.43 | 0.67 |
| CX3CL1 | 0.13 | 1.14 | 0.11 | 1.2 | 0.23 |
| CX3CL1 log(t + 0.01) | -0.06 | 0.94 | 0.05 | -1.05 | 0.29 |
| CXCL10 | 0.03 | 1.03 | 0.11 | 0.29 | 0.77 |
| CXCL10 log(t + 0.01) | 0.03 | 1.03 | 0.05 | 0.58 | 0.56 |
| CD70 | 0.2 | 1.22 | 0.12 | 1.6 | 0.11 |
| CD70 log(t + 0.01) | -0.07 | 0.93 | 0.06 | -1.18 | 0.24 |
| IL10 | 0.05 | 1.06 | 0.09 | 0.57 | 0.57 |
| IL10 log(t + 0.01) | 0 | 1 | 0.04 | 0 | 1 |
| TNFRSF12A | 0.18 | 1.2 | 0.11 | 1.62 | 0.11 |
| TNFRSF12A log(t + 0.01) | -0.05 | 0.95 | 0.05 | -1 | 0.32 |
| CCL23 | 0.28 | 1.32 | 0.1 | 2.69 | 0.01 |
| CCL23 log(t + 0.01) | -0.1 | 0.9 | 0.05 | -2.15 | 0.03 |
| CD5 | 0.1 | 1.1 | 0.11 | 0.92 | 0.36 |
| CD5 log(t + 0.01) | -0.01 | 0.99 | 0.05 | -0.17 | 0.86 |
| CCL3 | 0.23 | 1.26 | 0.08 | 2.81 | 0 |
| CCL3 log(t + 0.01) | -0.04 | 0.96 | 0.04 | -1.18 | 0.24 |
| MMP7 | 0.6 | 1.82 | 0.19 | 3.21 | 0 |
| MMP7 log(t + 0.01) | -0.3 | 0.74 | 0.09 | -3.23 | 0 |
| ARG1 | -0.08 | 0.92 | 0.1 | -0.85 | 0.4 |
| ARG1 log(t + 0.01) | -0.05 | 0.95 | 0.05 | -1.07 | 0.29 |
| NCR1 | 0.15 | 1.16 | 0.1 | 1.46 | 0.14 |
| NCR1 log(t + 0.01) | -0.07 | 0.93 | 0.05 | -1.43 | 0.15 |
| DCN | 0.14 | 1.15 | 0.11 | 1.26 | 0.21 |
| DCN log(t + 0.01) | -0.07 | 0.93 | 0.05 | -1.45 | 0.15 |
| TNFRSF21 | 0.13 | 1.13 | 0.11 | 1.11 | 0.27 |
| TNFRSF21 log(t + 0.01) | -0.08 | 0.92 | 0.05 | -1.5 | 0.13 |
|  |  |  |  |  |  |
|  | **logHR** | **HR** | **se(logHR)** | **z** | **Pr(>\|z\|)** |
| TNFRSF4 | 0.15 | 1.16 | 0.1 | 1.51 | 0.13 |
| TNFRSF4 log(t + 0.01) | -0.06 | 0.94 | 0.04 | -1.37 | 0.17 |
| MIC-A/B | -0.13 | 0.88 | 0.11 | -1.17 | 0.24 |
| MIC-A/B log(t + 0.01) | -0.01 | 0.99 | 0.05 | -0.18 | 0.86 |
| CCL17 | 0.18 | 1.2 | 0.12 | 1.56 | 0.12 |
| CCL17 log(t + 0.01) | -0.02 | 0.98 | 0.06 | -0.4 | 0.69 |
| ANGPT2 | 0.31 | 1.37 | 0.11 | 2.74 | 0.01 |
| ANGPT2 log(t + 0.01) | -0.05 | 0.96 | 0.05 | -0.84 | 0.4 |
| PTN | 0.15 | 1.16 | 0.1 | 1.44 | 0.15 |
| PTN log(t + 0.01) | -0.1 | 0.91 | 0.04 | -2.22 | 0.03 |
| CXCL12 | 0.11 | 1.11 | 0.1 | 1.13 | 0.26 |
| CXCL12 log(t + 0.01) | -0.08 | 0.93 | 0.04 | -1.78 | 0.08 |
| IFN-gamma | 0.03 | 1.03 | 0.11 | 0.25 | 0.81 |
| IFN-gamma log(t + 0.01) | -0.01 | 0.99 | 0.05 | -0.32 | 0.75 |
| LAMP3 | 0.27 | 1.31 | 0.12 | 2.28 | 0.02 |
| LAMP3 log(t + 0.01) | -0.02 | 0.98 | 0.05 | -0.41 | 0.68 |
| CASP8 | -0.06 | 0.94 | 0.1 | -0.63 | 0.53 |
| CASP8 log(t + 0.01) | -0.02 | 0.98 | 0.05 | -0.35 | 0.73 |
| ICOSLG | -0.12 | 0.89 | 0.1 | -1.17 | 0.24 |
| ICOSLG log(t + 0.01) | 0.04 | 1.04 | 0.05 | 0.72 | 0.47 |
| MMP12 | 0.27 | 1.31 | 0.1 | 2.61 | 0.01 |
| MMP12 log(t + 0.01) | -0.13 | 0.88 | 0.04 | -2.99 | 0 |
| CXCL13 | 0.15 | 1.16 | 0.11 | 1.29 | 0.2 |
| CXCL13 log(t + 0.01) | 0 | 1 | 0.06 | 0.06 | 0.95 |
| PD-L2 | 0.12 | 1.12 | 0.11 | 1.07 | 0.29 |
| PD-L2 log(t + 0.01) | -0.1 | 0.9 | 0.05 | -1.89 | 0.06 |
| VEGFA | 0.12 | 1.13 | 0.11 | 1.18 | 0.24 |
| VEGFA log(t + 0.01) | -0.03 | 0.97 | 0.05 | -0.51 | 0.61 |
| IL4 | -0.22 | 0.8 | 0.24 | -0.92 | 0.36 |
| IL4 log(t + 0.01) | 0.04 | 1.04 | 0.14 | 0.27 | 0.79 |
| IL12RB1 | 0.04 | 1.04 | 0.1 | 0.42 | 0.67 |
| IL12RB1 log(t + 0.01) | 0 | 1 | 0.05 | 0.02 | 0.99 |
| IL13 | 0.05 | 1.06 | 0.06 | 0.89 | 0.38 |
| IL13 log(t + 0.01) | -0.01 | 0.99 | 0.02 | -0.8 | 0.42 |
| CCL20 | -0.02 | 0.98 | 0.1 | -0.24 | 0.81 |
| CCL20 log(t + 0.01) | 0.01 | 1.01 | 0.05 | 0.14 | 0.89 |
| TNF | 0.05 | 1.05 | 0.08 | 0.61 | 0.54 |
| TNF log(t + 0.01) | 0.01 | 1.01 | 0.03 | 0.4 | 0.69 |
| KLRD1 | -0.07 | 0.93 | 0.1 | -0.66 | 0.51 |
| KLRD1 log(t + 0.01) | 0.01 | 1.01 | 0.05 | 0.25 | 0.8 |
| GZMB | 0.18 | 1.19 | 0.11 | 1.64 | 0.1 |
| GZMB log(t + 0.01) | -0.05 | 0.95 | 0.06 | -0.94 | 0.35 |
| CD83 | 0.2 | 1.22 | 0.11 | 1.77 | 0.08 |
| CD83 log(t + 0.01) | -0.06 | 0.94 | 0.05 | -1.11 | 0.27 |
| IL12 | 0.05 | 1.05 | 0.1 | 0.46 | 0.65 |
| IL12 log(t + 0.01) | 0 | 1 | 0.04 | -0.11 | 0.91 |
| CSF1 | 0.12 | 1.13 | 0.1 | 1.26 | 0.21 |
| CSF1 log(t + 0.01) | -0.07 | 0.93 | 0.04 | -1.54 | 0.12 |

# Table S7. Hazard ratios (95% CIs) for all proteins (per SD higher NPX), adjusted for age and sex and stratified by region, with age as the time scale and entry at age at baseline

|  | **logHR** | **HR** | **se(logHR)** | **95% CI** | **z** | **Pr(>\|z\|)** | **p_adj_** |
| --- | --- | --- | --- | --- | --- | --- | --- |
| MCP3/CCL7 | 0.24 | 1.28 | 0.07 | (1.11, 1.46) | 3.46 | 0 | 0.04 |
| IL18 | 0.23 | 1.26 | 0.07 | (1.10, 1.44) | 3.34 | 0 | 0.04 |
| ANGPT2 | 0.23 | 1.26 | 0.07 | (1.09, 1.44) | 3.25 | 0 | 0.04 |
| IL6 | 0.16 | 1.18 | 0.06 | (1.04, 1.33) | 2.67 | 0.01 | 0.12 |
| CD8A | 0.2 | 1.23 | 0.07 | (1.06, 1.41) | 2.77 | 0.01 | 0.12 |
| HO1 | 0.19 | 1.21 | 0.08 | (1.05, 1.40) | 2.55 | 0.01 | 0.14 |
| LAMP3 | 0.2 | 1.22 | 0.07 | (1.06, 1.42) | 2.69 | 0.01 | 0.12 |
| CRTAM | 0.18 | 1.2 | 0.08 | (1.03, 1.40) | 2.32 | 0.02 | 0.17 |
| CD4 | 0.15 | 1.17 | 0.07 | (1.02, 1.33) | 2.32 | 0.02 | 0.17 |
| CD40 | 0.18 | 1.2 | 0.08 | (1.03, 1.39) | 2.34 | 0.02 | 0.17 |
| HGF | 0.18 | 1.2 | 0.08 | (1.03, 1.40) | 2.4 | 0.02 | 0.17 |
| CXCL13 | 0.17 | 1.18 | 0.07 | (1.02, 1.37) | 2.24 | 0.02 | 0.18 |
| TNFRSF9 | 0.16 | 1.18 | 0.07 | (1.02, 1.36) | 2.22 | 0.03 | 0.18 |
| MIC-A/B | -0.15 | 0.86 | 0.07 | (0.75, 0.98) | -2.2 | 0.03 | 0.18 |
| IL5 | -0.17 | 0.84 | 0.08 | (0.72, 0.99) | -2.1 | 0.04 | 0.21 |
| GZMA | 0.17 | 1.18 | 0.08 | (1.01, 1.38) | 2.08 | 0.04 | 0.21 |
| CCL3 | 0.13 | 1.14 | 0.06 | (1.00, 1.29) | 2 | 0.05 | 0.24 |
| TIE2 | 0.13 | 1.14 | 0.07 | (0.99, 1.30) | 1.85 | 0.06 | 0.32 |
| ADGRG1 | 0.12 | 1.13 | 0.07 | (0.99, 1.30) | 1.77 | 0.08 | 0.36 |
| CCL17 | 0.13 | 1.14 | 0.08 | (0.98, 1.33) | 1.71 | 0.09 | 0.38 |
| CD83 | 0.12 | 1.13 | 0.07 | (0.98, 1.31) | 1.7 | 0.09 | 0.38 |
| TNFSF14 | 0.11 | 1.12 | 0.07 | (0.98, 1.27) | 1.62 | 0.1 | 0.39 |
| ARG1 | -0.12 | 0.89 | 0.07 | (0.77, 1.02) | -1.66 | 0.1 | 0.39 |
| CCL19 | 0.11 | 1.12 | 0.07 | (0.98, 1.29) | 1.61 | 0.11 | 0.39 |
| Gal-1 | 0.12 | 1.13 | 0.08 | (0.97, 1.31) | 1.59 | 0.11 | 0.39 |
| TNFRSF12A | 0.12 | 1.13 | 0.08 | (0.97, 1.32) | 1.6 | 0.11 | 0.39 |
| IFN-beta | -0.09 | 0.91 | 0.06 | (0.81, 1.02) | -1.57 | 0.12 | 0.39 |
| LAP TGF-beta-1 | 0.1 | 1.11 | 0.07 | (0.97, 1.27) | 1.49 | 0.14 | 0.44 |
| MMP7 | 0.12 | 1.13 | 0.08 | (0.96, 1.33) | 1.45 | 0.15 | 0.44 |
| MMP12 | 0.11 | 1.12 | 0.08 | (0.96, 1.31) | 1.43 | 0.15 | 0.44 |
| GZMB | 0.1 | 1.1 | 0.07 | (0.96, 1.27) | 1.43 | 0.15 | 0.44 |
| CAIX | 0.1 | 1.1 | 0.07 | (0.96, 1.26) | 1.41 | 0.16 | 0.44 |
| CCL23 | 0.1 | 1.1 | 0.07 | (0.96, 1.27) | 1.4 | 0.16 | 0.44 |
| ADA | 0.1 | 1.1 | 0.07 | (0.96, 1.27) | 1.38 | 0.17 | 0.44 |
| CD5 | 0.1 | 1.11 | 0.07 | (0.96, 1.28) | 1.36 | 0.17 | 0.44 |
| VEGFA | 0.09 | 1.09 | 0.07 | (0.94, 1.26) | 1.14 | 0.25 | 0.64 |
| NOS3 | 0.07 | 1.08 | 0.07 | (0.94, 1.23) | 1.11 | 0.27 | 0.64 |
| IL4 | -0.2 | 0.82 | 0.18 | (0.57, 1.17) | -1.11 | 0.27 | 0.64 |
| MCP2/CCL8 | -0.08 | 0.92 | 0.07 | (0.80, 1.07) | -1.08 | 0.28 | 0.64 |
| GZMH | 0.07 | 1.07 | 0.07 | (0.94, 1.23) | 1.03 | 0.3 | 0.64 |
| CD70 | 0.08 | 1.08 | 0.08 | (0.93, 1.26) | 1.04 | 0.3 | 0.64 |
| CD40L | 0.07 | 1.08 | 0.07 | (0.93, 1.24) | 1.02 | 0.31 | 0.64 |
|  | **logHR** | **HR** | **se(logHR)** | **95% CI** | **z** | **Pr(>\|z\|)** | **p_adj_** |
| CX3CL1 | 0.08 | 1.08 | 0.08 | (0.93, 1.25) | 0.99 | 0.32 | 0.64 |
| PDCD1 | 0.06 | 1.06 | 0.06 | (0.94, 1.18) | 0.95 | 0.34 | 0.64 |
| TNFRSF4 | 0.07 | 1.07 | 0.07 | (0.93, 1.24) | 0.96 | 0.34 | 0.64 |
| PGF | 0.07 | 1.07 | 0.08 | (0.93, 1.24) | 0.94 | 0.35 | 0.64 |
| TNF | 0.06 | 1.06 | 0.06 | (0.94, 1.20) | 0.94 | 0.35 | 0.64 |
| VEGFR2 | 0.06 | 1.06 | 0.07 | (0.93, 1.21) | 0.91 | 0.36 | 0.64 |
| ICOSLG | -0.07 | 0.94 | 0.07 | (0.81, 1.08) | -0.92 | 0.36 | 0.64 |
| DCN | 0.07 | 1.07 | 0.07 | (0.93, 1.23) | 0.9 | 0.37 | 0.64 |
| CCL4 | 0.06 | 1.06 | 0.07 | (0.93, 1.21) | 0.87 | 0.38 | 0.64 |
| IL12RB1 | 0.06 | 1.06 | 0.07 | (0.93, 1.21) | 0.89 | 0.38 | 0.64 |
| Gal-9 | 0.07 | 1.08 | 0.09 | (0.91, 1.27) | 0.85 | 0.4 | 0.64 |
| IL33 | -0.09 | 0.92 | 0.1 | (0.75, 1.12) | -0.84 | 0.4 | 0.64 |
| CASP8 | -0.06 | 0.94 | 0.07 | (0.82, 1.08) | -0.84 | 0.4 | 0.64 |
| MCP1/CCL2 | 0.06 | 1.06 | 0.07 | (0.92, 1.23) | 0.82 | 0.41 | 0.64 |
| TWEAK | -0.06 | 0.94 | 0.07 | (0.82, 1.08) | -0.83 | 0.41 | 0.64 |
| IL13 | 0.04 | 1.04 | 0.05 | (0.95, 1.14) | 0.82 | 0.41 | 0.64 |
| CD28 | 0.06 | 1.06 | 0.07 | (0.92, 1.21) | 0.79 | 0.43 | 0.66 |
| IL2 | 0.41 | 1.51 | 0.56 | (0.50, 4.57) | 0.73 | 0.46 | 0.7 |
| IL10 | 0.05 | 1.05 | 0.07 | (0.91, 1.20) | 0.66 | 0.51 | 0.74 |
| NCR1 | 0.05 | 1.05 | 0.07 | (0.91, 1.20) | 0.66 | 0.51 | 0.74 |
| CXCL10 | 0.05 | 1.05 | 0.08 | (0.91, 1.22) | 0.65 | 0.52 | 0.74 |
| CD27 | 0.05 | 1.05 | 0.08 | (0.90, 1.22) | 0.61 | 0.54 | 0.75 |
| CCL20 | 0.04 | 1.04 | 0.07 | (0.91, 1.18) | 0.59 | 0.55 | 0.75 |
| TRAIL | -0.04 | 0.96 | 0.06 | (0.85, 1.09) | -0.58 | 0.56 | 0.75 |
| CSF1 | 0.04 | 1.04 | 0.07 | (0.91, 1.19) | 0.59 | 0.56 | 0.75 |
| IL8 | 0.03 | 1.03 | 0.06 | (0.91, 1.17) | 0.52 | 0.6 | 0.8 |
| CXCL9 | 0.04 | 1.04 | 0.08 | (0.89, 1.21) | 0.49 | 0.63 | 0.82 |
| IL12 | 0.03 | 1.04 | 0.07 | (0.90, 1.20) | 0.47 | 0.64 | 0.82 |
| FasLG | -0.03 | 0.97 | 0.07 | (0.84, 1.12) | -0.45 | 0.65 | 0.82 |
| PDGF subunit B | 0.03 | 1.03 | 0.07 | (0.89, 1.19) | 0.42 | 0.68 | 0.84 |
| CXCL5 | 0.03 | 1.03 | 0.07 | (0.89, 1.19) | 0.39 | 0.7 | 0.86 |
| PD-L1 | 0.03 | 1.03 | 0.07 | (0.90, 1.17) | 0.38 | 0.71 | 0.86 |
| FGF2 | 0.02 | 1.02 | 0.06 | (0.90, 1.16) | 0.35 | 0.73 | 0.87 |
| PTN | 0.03 | 1.03 | 0.09 | (0.86, 1.23) | 0.33 | 0.74 | 0.87 |
| CXCL11 | -0.02 | 0.98 | 0.07 | (0.85, 1.12) | -0.31 | 0.75 | 0.87 |
| IFN-gamma | -0.03 | 0.97 | 0.1 | (0.80, 1.18) | -0.33 | 0.75 | 0.87 |
| ANG1 | 0.02 | 1.02 | 0.07 | (0.88, 1.18) | 0.29 | 0.77 | 0.88 |
| CXCL12 | -0.02 | 0.98 | 0.08 | (0.84, 1.15) | -0.23 | 0.82 | 0.92 |
| TNFRSF21 | 0.01 | 1.01 | 0.07 | (0.88, 1.17) | 0.2 | 0.84 | 0.93 |
| MCP4 | -0.01 | 0.99 | 0.08 | (0.85, 1.15) | -0.19 | 0.85 | 0.93 |
| EGF | -0.01 | 0.99 | 0.07 | (0.86, 1.13) | -0.17 | 0.86 | 0.93 |
| IL7 | 0.01 | 1.01 | 0.07 | (0.89, 1.15) | 0.12 | 0.91 | 0.97 |
| CXCL1 | -0.01 | 0.99 | 0.07 | (0.86, 1.15) | -0.08 | 0.93 | 0.98 |
| IL1 alpha | 0 | 1 | 0.07 | (0.88, 1.13) | -0.06 | 0.95 | 0.98 |
| KLRD1 | 0 | 1 | 0.07 | (0.87, 1.15) | 0.06 | 0.95 | 0.98 |
| PD-L2 | 0 | 1 | 0.07 | (0.87, 1.15) | 0.02 | 0.98 | 1 |
| CD244 | 0 | 1 | 0.07 | (0.87, 1.15) | 0 | 1 | 1 |
| VEGFC | 0 | 1 | 0.07 | (0.87, 1.15) | 0 | 1 | 1 |

# Table S8. Hazard ratios (95% CIs) for all proteins (per SD higher NPX), adjusted for age, age^2^, sex, region, smoking, alcohol, education, diabetes, fasting time and stratified by region, with age as the time scale and entry at age at baseline

|  | **logHR** | **HR** | **se(logHR)** | **95% CI** | **z** | **Pr(>\|z\|)** | **p_adj_** |
| --- | --- | --- | --- | --- | --- | --- | --- |
| MCP3/CCL7 | 0.25 | 1.29 | 0.08 | (1.10, 1.51) | 3.19 | 0 | 0.07 |
| IL6 | 0.19 | 1.21 | 0.07 | (1.06, 1.38) | 2.82 | 0 | 0.11 |
| IL18 | 0.21 | 1.24 | 0.08 | (1.07, 1.43) | 2.83 | 0 | 0.11 |
| ANGPT2 | 0.24 | 1.27 | 0.08 | (1.10, 1.48) | 3.17 | 0 | 0.07 |
| CD8A | 0.19 | 1.21 | 0.08 | (1.04, 1.40) | 2.44 | 0.01 | 0.16 |
| CD4 | 0.16 | 1.18 | 0.07 | (1.03, 1.34) | 2.45 | 0.01 | 0.16 |
| CCL3 | 0.17 | 1.18 | 0.07 | (1.04, 1.35) | 2.48 | 0.01 | 0.16 |
| LAMP3 | 0.23 | 1.26 | 0.09 | (1.06, 1.50) | 2.62 | 0.01 | 0.15 |
| HGF | 0.19 | 1.21 | 0.08 | (1.03, 1.42) | 2.28 | 0.02 | 0.2 |
| HO1 | 0.18 | 1.2 | 0.08 | (1.03, 1.40) | 2.29 | 0.02 | 0.2 |
| IL2 | 0.62 | 1.86 | 0.28 | (1.08, 3.20) | 2.24 | 0.03 | 0.2 |
| GZMA | 0.18 | 1.2 | 0.09 | (1.01, 1.43) | 2.09 | 0.04 | 0.25 |
| IL4 | -0.16 | 0.86 | 0.07 | (0.74, 0.99) | -2.11 | 0.04 | 0.25 |
| ADGRG1 | 0.14 | 1.15 | 0.07 | (1.00, 1.33) | 1.97 | 0.05 | 0.29 |
| CRTAM | 0.17 | 1.18 | 0.09 | (1.00, 1.40) | 1.97 | 0.05 | 0.29 |
| ARG1 | -0.15 | 0.86 | 0.08 | (0.74, 1.00) | -1.92 | 0.05 | 0.29 |
| CD40 | 0.15 | 1.16 | 0.08 | (0.99, 1.35) | 1.87 | 0.06 | 0.29 |
| Gal-1 | 0.16 | 1.17 | 0.08 | (1.00, 1.38) | 1.91 | 0.06 | 0.29 |
| IL5 | -0.15 | 0.86 | 0.08 | (0.73, 1.01) | -1.86 | 0.06 | 0.29 |
| CXCL13 | 0.15 | 1.16 | 0.08 | (0.99, 1.36) | 1.85 | 0.06 | 0.29 |
| CCL23 | 0.14 | 1.15 | 0.07 | (0.99, 1.33) | 1.82 | 0.07 | 0.29 |
| TNFRSF9 | 0.14 | 1.15 | 0.08 | (0.98, 1.34) | 1.73 | 0.08 | 0.31 |
| MIC-A/B | -0.13 | 0.87 | 0.08 | (0.75, 1.02) | -1.75 | 0.08 | 0.31 |
| CCL17 | 0.15 | 1.16 | 0.08 | (0.98, 1.37) | 1.74 | 0.08 | 0.31 |
| IFN-beta | -0.09 | 0.91 | 0.05 | (0.82, 1.02) | -1.64 | 0.1 | 0.36 |
| CCL19 | 0.12 | 1.13 | 0.08 | (0.97, 1.31) | 1.61 | 0.11 | 0.37 |
| TIE2 | 0.12 | 1.12 | 0.08 | (0.97, 1.30) | 1.55 | 0.12 | 0.39 |
| MMP7 | 0.14 | 1.15 | 0.09 | (0.97, 1.36) | 1.57 | 0.12 | 0.39 |
| PDCD1 | 0.08 | 1.09 | 0.06 | (0.97, 1.22) | 1.44 | 0.15 | 0.45 |
| CD83 | 0.12 | 1.12 | 0.08 | (0.96, 1.31) | 1.45 | 0.15 | 0.45 |
| GZMB | 0.1 | 1.11 | 0.07 | (0.96, 1.27) | 1.41 | 0.16 | 0.45 |
| CCL4 | 0.1 | 1.1 | 0.07 | (0.96, 1.26) | 1.36 | 0.17 | 0.47 |
| TNFRSF12A | 0.11 | 1.12 | 0.08 | (0.95, 1.31) | 1.36 | 0.17 | 0.47 |
| ADA | 0.09 | 1.09 | 0.07 | (0.95, 1.27) | 1.22 | 0.22 | 0.58 |
| TNFSF14 | 0.08 | 1.09 | 0.07 | (0.95, 1.25) | 1.18 | 0.24 | 0.61 |
| CASP8 | -0.08 | 0.92 | 0.07 | (0.80, 1.06) | -1.14 | 0.25 | 0.61 |
| CD70 | 0.09 | 1.1 | 0.08 | (0.93, 1.29) | 1.12 | 0.26 | 0.61 |
| VEGFA | 0.09 | 1.09 | 0.08 | (0.94, 1.27) | 1.13 | 0.26 | 0.61 |
| CD5 | 0.09 | 1.09 | 0.08 | (0.93, 1.27) | 1.1 | 0.27 | 0.62 |
| MMP12 | 0.09 | 1.1 | 0.09 | (0.93, 1.30) | 1.08 | 0.28 | 0.62 |
| LAP TGF-beta-1 | 0.07 | 1.08 | 0.08 | (0.93, 1.25) | 0.99 | 0.32 | 0.68 |
| TNF | 0.06 | 1.07 | 0.06 | (0.94, 1.21) | 1 | 0.32 | 0.68 |
|  | **logHR** | **HR** | **se(logHR)** | **95% CI** | **z** | **Pr(>\|z\|)** | **p_adj_** |
| NOS3 | 0.07 | 1.07 | 0.08 | (0.93, 1.25) | 0.94 | 0.35 | 0.72 |
| CXCL10 | 0.07 | 1.08 | 0.08 | (0.92, 1.26) | 0.91 | 0.36 | 0.72 |
| CXCL9 | 0.07 | 1.07 | 0.08 | (0.92, 1.26) | 0.89 | 0.38 | 0.72 |
| TNFRSF4 | 0.07 | 1.07 | 0.08 | (0.92, 1.25) | 0.88 | 0.38 | 0.72 |
| ICOSLG | -0.07 | 0.94 | 0.07 | (0.81, 1.08) | -0.89 | 0.38 | 0.72 |
| Gal-9 | 0.08 | 1.08 | 0.09 | (0.90, 1.30) | 0.86 | 0.39 | 0.72 |
| PGF | 0.06 | 1.06 | 0.08 | (0.91, 1.24) | 0.8 | 0.42 | 0.72 |
| IL10 | 0.06 | 1.06 | 0.07 | (0.92, 1.22) | 0.78 | 0.43 | 0.72 |
| EGF | -0.06 | 0.94 | 0.08 | (0.81, 1.10) | -0.77 | 0.44 | 0.72 |
| TWEAK | -0.06 | 0.95 | 0.07 | (0.82, 1.09) | -0.77 | 0.44 | 0.72 |
| GZMH | 0.05 | 1.05 | 0.07 | (0.92, 1.21) | 0.76 | 0.45 | 0.72 |
| MCP1/CCL2 | 0.06 | 1.06 | 0.08 | (0.91, 1.24) | 0.72 | 0.47 | 0.72 |
| MCP4 | -0.06 | 0.94 | 0.08 | (0.80, 1.11) | -0.72 | 0.47 | 0.72 |
| IL33 | -0.05 | 0.95 | 0.07 | (0.84, 1.08) | -0.72 | 0.47 | 0.72 |
| CX3CL1 | 0.06 | 1.06 | 0.08 | (0.91, 1.23) | 0.72 | 0.47 | 0.72 |
| NCR1 | 0.05 | 1.05 | 0.07 | (0.91, 1.22) | 0.73 | 0.47 | 0.72 |
| MCP2/CCL8 | -0.06 | 0.95 | 0.08 | (0.81, 1.10) | -0.71 | 0.48 | 0.72 |
| VEGFR2 | 0.05 | 1.05 | 0.07 | (0.92, 1.20) | 0.68 | 0.49 | 0.72 |
| IL12RB1 | 0.05 | 1.05 | 0.07 | (0.92, 1.20) | 0.69 | 0.49 | 0.72 |
| CXCL1 | -0.05 | 0.95 | 0.08 | (0.81, 1.11) | -0.65 | 0.51 | 0.73 |
| CD28 | 0.05 | 1.05 | 0.08 | (0.90, 1.23) | 0.66 | 0.51 | 0.73 |
| KLRD1 | -0.05 | 0.95 | 0.08 | (0.82, 1.11) | -0.6 | 0.55 | 0.76 |
| IL12 | 0.04 | 1.05 | 0.08 | (0.89, 1.22) | 0.56 | 0.57 | 0.79 |
| CAIX | 0.04 | 1.04 | 0.08 | (0.89, 1.22) | 0.52 | 0.6 | 0.81 |
| DCN | 0.04 | 1.04 | 0.08 | (0.89, 1.21) | 0.5 | 0.62 | 0.82 |
| FasLG | -0.03 | 0.97 | 0.08 | (0.83, 1.13) | -0.44 | 0.66 | 0.82 |
| CSF1 | 0.03 | 1.03 | 0.07 | (0.90, 1.19) | 0.43 | 0.67 | 0.82 |
| CD40L | 0.03 | 1.03 | 0.08 | (0.89, 1.20) | 0.41 | 0.68 | 0.82 |
| CD244 | -0.03 | 0.97 | 0.08 | (0.83, 1.13) | -0.42 | 0.68 | 0.82 |
| PTN | 0.04 | 1.04 | 0.09 | (0.86, 1.25) | 0.42 | 0.68 | 0.82 |
| IL8 | -0.03 | 0.97 | 0.07 | (0.85, 1.12) | -0.4 | 0.69 | 0.82 |
| ANG1 | -0.03 | 0.97 | 0.08 | (0.82, 1.14) | -0.39 | 0.69 | 0.82 |
| IL7 | -0.03 | 0.97 | 0.07 | (0.84, 1.12) | -0.4 | 0.69 | 0.82 |
| CXCL11 | -0.03 | 0.97 | 0.08 | (0.84, 1.13) | -0.34 | 0.74 | 0.86 |
| CD27 | 0.02 | 1.02 | 0.08 | (0.87, 1.20) | 0.28 | 0.78 | 0.9 |
| PD-L2 | -0.02 | 0.98 | 0.08 | (0.84, 1.14) | -0.26 | 0.79 | 0.9 |
| CXCL5 | -0.02 | 0.98 | 0.08 | (0.83, 1.16) | -0.24 | 0.81 | 0.91 |
| VEGFC | -0.02 | 0.98 | 0.07 | (0.85, 1.14) | -0.23 | 0.82 | 0.91 |
| PDGF subunit B | -0.02 | 0.98 | 0.08 | (0.84, 1.15) | -0.22 | 0.83 | 0.91 |
| TNFRSF21 | 0.02 | 1.02 | 0.08 | (0.87, 1.18) | 0.21 | 0.84 | 0.91 |
| CCL20 | -0.01 | 0.99 | 0.07 | (0.86, 1.14) | -0.19 | 0.85 | 0.91 |
| FGF2 | -0.01 | 0.99 | 0.07 | (0.87, 1.13) | -0.16 | 0.88 | 0.93 |
| PD-L1 | -0.01 | 0.99 | 0.07 | (0.86, 1.14) | -0.14 | 0.89 | 0.93 |
| IL1 alpha | -0.01 | 0.99 | 0.08 | (0.85, 1.15) | -0.12 | 0.91 | 0.93 |
| TRAIL | -0.01 | 0.99 | 0.06 | (0.88, 1.12) | -0.1 | 0.92 | 0.93 |
| IFN-gamma | 0.01 | 1.01 | 0.08 | (0.87, 1.17) | 0.1 | 0.92 | 0.93 |
| CXCL12 | 0.01 | 1.01 | 0.08 | (0.86, 1.18) | 0.07 | 0.94 | 0.94 |

# Table S9. Hazard ratios (95% CIs) for all proteins (per SD higher NPX), adjusted for age and sex and stratified by region, with time in study as the time scale; no exclusions (using all individuals)

|  | **logHR** | **HR** | **se(logHR)** | **95% CI** | **z** | **Pr(>\|z\|)** | **p_adj_** |
| --- | --- | --- | --- | --- | --- | --- | --- |
| TNFRSF9 | 0.21 | 1.23 | 0.07 | (1.07, 1.42) | 2.85 | 0 | 0.04 |
| MCP3/CCL7 | 0.3 | 1.35 | 0.07 | (1.18, 1.55) | 4.26 | 0 | 0 |
| IL6 | 0.2 | 1.23 | 0.06 | (1.09, 1.38) | 3.51 | 0 | 0.01 |
| CD8A | 0.21 | 1.23 | 0.07 | (1.07, 1.42) | 2.89 | 0 | 0.03 |
| CD40 | 0.21 | 1.24 | 0.07 | (1.08, 1.42) | 3.05 | 0 | 0.02 |
| IL18 | 0.26 | 1.3 | 0.07 | (1.13, 1.49) | 3.68 | 0 | 0.01 |
| HGF | 0.24 | 1.27 | 0.07 | (1.10, 1.47) | 3.24 | 0 | 0.01 |
| CCL3 | 0.22 | 1.25 | 0.06 | (1.10, 1.42) | 3.45 | 0 | 0.01 |
| ANGPT2 | 0.24 | 1.27 | 0.07 | (1.12, 1.46) | 3.58 | 0 | 0.01 |
| LAMP3 | 0.25 | 1.29 | 0.07 | (1.11, 1.49) | 3.41 | 0 | 0.01 |
| CXCL13 | 0.25 | 1.29 | 0.08 | (1.11, 1.49) | 3.34 | 0 | 0.01 |
| TIE2 | 0.18 | 1.2 | 0.07 | (1.05, 1.38) | 2.64 | 0.01 | 0.06 |
| CD4 | 0.17 | 1.19 | 0.06 | (1.05, 1.35) | 2.68 | 0.01 | 0.05 |
| HO1 | 0.19 | 1.21 | 0.07 | (1.05, 1.40) | 2.57 | 0.01 | 0.06 |
| TNFRSF12A | 0.18 | 1.2 | 0.07 | (1.04, 1.38) | 2.56 | 0.01 | 0.06 |
| CCL17 | 0.19 | 1.21 | 0.08 | (1.04, 1.41) | 2.45 | 0.01 | 0.08 |
| CRTAM | 0.18 | 1.2 | 0.08 | (1.03, 1.39) | 2.35 | 0.02 | 0.09 |
| TNFSF14 | 0.16 | 1.17 | 0.07 | (1.03, 1.34) | 2.38 | 0.02 | 0.09 |
| CD83 | 0.17 | 1.18 | 0.07 | (1.03, 1.36) | 2.33 | 0.02 | 0.09 |
| IL33 | -0.16 | 0.86 | 0.07 | (0.75, 0.98) | -2.21 | 0.03 | 0.11 |
| CCL19 | 0.15 | 1.17 | 0.07 | (1.02, 1.33) | 2.24 | 0.03 | 0.11 |
| CCL4 | 0.15 | 1.17 | 0.07 | (1.02, 1.34) | 2.22 | 0.03 | 0.11 |
| Gal-1 | 0.16 | 1.18 | 0.08 | (1.02, 1.37) | 2.17 | 0.03 | 0.11 |
| VEGFA | 0.15 | 1.17 | 0.07 | (1.02, 1.34) | 2.22 | 0.03 | 0.11 |
| GZMA | 0.16 | 1.17 | 0.08 | (1.01, 1.37) | 2.04 | 0.04 | 0.14 |
| CCL23 | 0.15 | 1.16 | 0.07 | (1.01, 1.33) | 2.06 | 0.04 | 0.14 |
| LAP TGF-beta-1 | 0.13 | 1.14 | 0.07 | (1.00, 1.30) | 2 | 0.05 | 0.15 |
| MMP12 | 0.16 | 1.17 | 0.08 | (1.00, 1.37) | 1.96 | 0.05 | 0.16 |
| MMP7 | 0.15 | 1.16 | 0.08 | (0.99, 1.36) | 1.83 | 0.07 | 0.21 |
| ADGRG1 | 0.12 | 1.13 | 0.07 | (0.99, 1.29) | 1.77 | 0.08 | 0.22 |
| Gal-9 | 0.15 | 1.16 | 0.08 | (0.98, 1.36) | 1.77 | 0.08 | 0.22 |
| CD27 | 0.12 | 1.13 | 0.07 | (0.98, 1.31) | 1.73 | 0.08 | 0.23 |
| CD5 | 0.13 | 1.13 | 0.07 | (0.98, 1.31) | 1.74 | 0.08 | 0.23 |
| IFN-beta | -0.11 | 0.9 | 0.07 | (0.78, 1.02) | -1.63 | 0.1 | 0.24 |
| ADA | 0.12 | 1.13 | 0.07 | (0.98, 1.30) | 1.66 | 0.1 | 0.24 |
| PDCD1 | 0.09 | 1.1 | 0.06 | (0.98, 1.23) | 1.64 | 0.1 | 0.24 |
| IL4 | -0.1 | 0.91 | 0.06 | (0.81, 1.02) | -1.65 | 0.1 | 0.24 |
| MCP1/CCL2 | 0.11 | 1.12 | 0.07 | (0.98, 1.28) | 1.6 | 0.11 | 0.24 |
| CAIX | 0.12 | 1.12 | 0.07 | (0.98, 1.29) | 1.62 | 0.11 | 0.24 |
| VEGFR2 | 0.1 | 1.11 | 0.06 | (0.98, 1.26) | 1.62 | 0.11 | 0.24 |
| GZMB | 0.11 | 1.11 | 0.07 | (0.97, 1.28) | 1.54 | 0.12 | 0.27 |
| IL10 | 0.1 | 1.1 | 0.07 | (0.97, 1.25) | 1.47 | 0.14 | 0.3 |
|  | **logHR** | **HR** | **se(logHR)** | **95% CI** | **z** | **Pr(>\|z\|)** | **p_adj_** |
| IL5 | -0.11 | 0.89 | 0.08 | (0.77, 1.04) | -1.45 | 0.15 | 0.3 |
| CXCL10 | 0.11 | 1.11 | 0.07 | (0.96, 1.29) | 1.44 | 0.15 | 0.3 |
| ARG1 | -0.1 | 0.9 | 0.07 | (0.79, 1.04) | -1.44 | 0.15 | 0.3 |
| CD40L | 0.1 | 1.1 | 0.07 | (0.96, 1.27) | 1.41 | 0.16 | 0.31 |
| NCR1 | 0.09 | 1.1 | 0.07 | (0.96, 1.25) | 1.39 | 0.16 | 0.31 |
| CSF1 | 0.09 | 1.1 | 0.07 | (0.96, 1.25) | 1.4 | 0.16 | 0.31 |
| IL2 | -0.11 | 0.9 | 0.08 | (0.77, 1.05) | -1.35 | 0.18 | 0.32 |
| TNFRSF4 | 0.1 | 1.1 | 0.07 | (0.95, 1.27) | 1.32 | 0.19 | 0.33 |
| IFN-gamma | -0.11 | 0.9 | 0.08 | (0.76, 1.06) | -1.29 | 0.2 | 0.35 |
| PGF | 0.08 | 1.08 | 0.07 | (0.95, 1.24) | 1.16 | 0.25 | 0.43 |
| CD70 | 0.08 | 1.09 | 0.08 | (0.94, 1.27) | 1.1 | 0.27 | 0.45 |
| MIC-A/B | -0.08 | 0.92 | 0.07 | (0.80, 1.06) | -1.11 | 0.27 | 0.45 |
| CXCL9 | 0.07 | 1.08 | 0.07 | (0.94, 1.24) | 1.04 | 0.3 | 0.49 |
| CCL20 | 0.07 | 1.07 | 0.07 | (0.94, 1.21) | 0.99 | 0.32 | 0.52 |
| CX3CL1 | 0.07 | 1.07 | 0.07 | (0.93, 1.23) | 0.97 | 0.33 | 0.53 |
| MCP4 | 0.07 | 1.07 | 0.08 | (0.92, 1.25) | 0.91 | 0.36 | 0.55 |
| CXCL12 | -0.06 | 0.94 | 0.07 | (0.83, 1.07) | -0.91 | 0.36 | 0.55 |
| TNF | 0.05 | 1.06 | 0.06 | (0.94, 1.19) | 0.92 | 0.36 | 0.55 |
| CASP8 | -0.06 | 0.94 | 0.07 | (0.82, 1.08) | -0.88 | 0.38 | 0.55 |
| IL12RB1 | 0.06 | 1.06 | 0.06 | (0.93, 1.20) | 0.88 | 0.38 | 0.55 |
| IL8 | 0.05 | 1.05 | 0.06 | (0.93, 1.19) | 0.83 | 0.41 | 0.57 |
| ANG1 | 0.06 | 1.06 | 0.08 | (0.92, 1.24) | 0.82 | 0.41 | 0.57 |
| ICOSLG | -0.06 | 0.94 | 0.07 | (0.82, 1.08) | -0.82 | 0.41 | 0.57 |
| TNFRSF21 | 0.06 | 1.06 | 0.07 | (0.92, 1.22) | 0.81 | 0.42 | 0.57 |
| PDGF subunit B | 0.06 | 1.06 | 0.07 | (0.92, 1.23) | 0.79 | 0.43 | 0.58 |
| CXCL5 | 0.06 | 1.06 | 0.08 | (0.91, 1.24) | 0.77 | 0.44 | 0.59 |
| GZMH | 0.05 | 1.05 | 0.07 | (0.92, 1.21) | 0.72 | 0.47 | 0.61 |
| IL12 | 0.04 | 1.04 | 0.07 | (0.90, 1.20) | 0.55 | 0.58 | 0.75 |
| KLRD1 | 0.03 | 1.04 | 0.07 | (0.90, 1.19) | 0.49 | 0.62 | 0.79 |
| CXCL11 | 0.03 | 1.03 | 0.07 | (0.90, 1.17) | 0.4 | 0.69 | 0.83 |
| CXCL1 | 0.03 | 1.03 | 0.08 | (0.89, 1.20) | 0.4 | 0.69 | 0.83 |
| TWEAK | 0.03 | 1.03 | 0.07 | (0.90, 1.17) | 0.39 | 0.69 | 0.83 |
| FasLG | 0.03 | 1.03 | 0.07 | (0.89, 1.18) | 0.38 | 0.7 | 0.83 |
| CD28 | 0.03 | 1.03 | 0.07 | (0.89, 1.19) | 0.37 | 0.71 | 0.83 |
| DCN | 0.03 | 1.03 | 0.07 | (0.90, 1.17) | 0.37 | 0.71 | 0.83 |
| PD-L1 | 0.02 | 1.02 | 0.06 | (0.91, 1.15) | 0.35 | 0.73 | 0.84 |
| VEGFC | 0.02 | 1.02 | 0.07 | (0.89, 1.17) | 0.3 | 0.76 | 0.87 |
| CD244 | 0.02 | 1.02 | 0.07 | (0.88, 1.17) | 0.24 | 0.81 | 0.9 |
| IL1 alpha | 0.01 | 1.01 | 0.06 | (0.90, 1.15) | 0.23 | 0.82 | 0.9 |
| IL7 | 0.02 | 1.02 | 0.06 | (0.89, 1.15) | 0.23 | 0.82 | 0.9 |
| TRAIL | 0.01 | 1.01 | 0.06 | (0.90, 1.14) | 0.22 | 0.83 | 0.9 |
| PD-L2 | 0.01 | 1.01 | 0.07 | (0.89, 1.15) | 0.17 | 0.87 | 0.92 |
| EGF | 0.01 | 1.01 | 0.07 | (0.88, 1.16) | 0.15 | 0.88 | 0.92 |
| IL13 | -0.01 | 0.99 | 0.07 | (0.86, 1.14) | -0.15 | 0.88 | 0.92 |
| PTN | -0.01 | 0.99 | 0.09 | (0.82, 1.18) | -0.13 | 0.9 | 0.93 |
| NOS3 | 0.01 | 1.01 | 0.07 | (0.88, 1.15) | 0.11 | 0.92 | 0.94 |
| FGF2 | -0.01 | 0.99 | 0.07 | (0.87, 1.14) | -0.08 | 0.94 | 0.94 |
| MCP2/CCL8 | 0.01 | 1.01 | 0.08 | (0.86, 1.17) | 0.07 | 0.94 | 0.94 |

# Table S10. Hazard ratios (95% CIs) for all proteins (per SD higher NPX) from multivariable models, identified using the Cox­–Battey approach, adjusted for age, age^2^, sex, smoking, alcohol drinking, education, diabetes, time since last meal, and family history of cancer, stratified by region, with time in study as the time scale.

Variables are selected using each of 3 criteria: (a) the 3 most significant variables from each regression, (b) the 2 most significant variables from each regression, and (c) variables with │z│ > 2.5.

**(a) 3 most significant**

|  | **logHR** | **HR** | **se(logHR)** | **z** | **p** |
| --- | --- | --- | --- | --- | --- |
| ARG1 | -0.36 | 0.70 | 0.12 | -2.95 | <0.01 |
| KLRD1 | -0.61 | 0.55 | 0.21 | -2.90 | <0.01 |
| MIC-A/B | -0.15 | 0.86 | 0.05 | -2.77 | 0.01 |
| TNFRSF21 | -1.20 | 0.30 | 0.51 | -2.35 | 0.02 |
| IL5 | -0.22 | 0.80 | 0.10 | -2.27 | 0.02 |
| ADGRG1 | 0.42 | 1.52 | 0.19 | 2.19 | 0.03 |
| IL4 | -1.86 | 0.16 | 0.90 | -2.06 | 0.04 |
| CD4 | 0.49 | 1.63 | 0.27 | 1.79 | 0.07 |
| ANGPT2 | 0.41 | 1.50 | 0.23 | 1.75 | 0.08 |
| CRTAM | 0.38 | 1.46 | 0.22 | 1.74 | 0.08 |
| MCP2 | -0.17 | 0.84 | 0.10 | -1.66 | 0.10 |
| HO1 | 0.37 | 1.45 | 0.23 | 1.63 | 0.10 |
| CD244 | -0.52 | 0.59 | 0.34 | -1.53 | 0.13 |
| MCP3 | 0.40 | 1.49 | 0.26 | 1.53 | 0.13 |
| CD8A | 0.18 | 1.20 | 0.12 | 1.51 | 0.13 |
| IFN-beta | -0.94 | 0.39 | 0.63 | -1.49 | 0.14 |
| LAMP3 | 0.22 | 1.24 | 0.16 | 1.39 | 0.16 |
| IL18 | 0.26 | 1.30 | 0.21 | 1.25 | 0.21 |
| CCL23 | 0.21 | 1.23 | 0.20 | 1.06 | 0.29 |
| TNF | 0.12 | 1.13 | 0.12 | 1.00 | 0.32 |
| IL2 | 20.85 | 1.14e9 | 22.32 | 0.93 | 0.35 |
| CCL19 | 0.12 | 1.12 | 0.13 | 0.92 | 0.36 |
| MMP7 | 0.06 | 1.06 | 0.08 | 0.71 | 0.48 |
| HGF | 0.21 | 1.23 | 0.31 | 0.66 | 0.51 |
| CCL3 | 0.10 | 1.11 | 0.16 | 0.62 | 0.53 |
| LAP-TGF-beta1 | -0.20 | 0.82 | 0.35 | -0.56 | 0.57 |
| CD40 | 0.10 | 1.11 | 0.26 | 0.38 | 0.70 |
| Gal-1 | 0.16 | 1.17 | 0.45 | 0.36 | 0.72 |
| ICOSLG | -0.12 | 0.89 | 0.34 | -0.35 | 0.72 |
| IL6 | -0.03 | 0.97 | 0.11 | -0.24 | 0.81 |
| CXCL13 | -0.03 | 0.97 | 0.19 | -0.18 | 0.86 |

**(b) 2 most significant**

|  | **logHR** | **HR** | **se(logHR)** | **z** | **p** |
| --- | --- | --- | --- | --- | --- |
| ARG1 | -0.37 | 0.69 | 0.12 | -3.17 | <0.01 |
| IL2 | 47.29 | 3.4e20 | 25.08 | 1.89 | 0.06 |
| IL4 | -2.25 | 0.11 | 1.20 | -1.88 | 0.06 |
| CD8A | 0.18 | 1.20 | 0.11 | 1.69 | 0.09 |
| MCP3 | 0.39 | 1.48 | 0.25 | 1.58 | 0.11 |
| IFN-beta | -0.95 | 0.39 | 0.64 | -1.48 | 0.14 |
| ANGPT2 | 0.28 | 1.32 | 0.21 | 1.32 | 0.19 |
| HO1 | 0.25 | 1.29 | 0.21 | 1.19 | 0.23 |
| LAMP3 | 0.14 | 1.15 | 0.14 | 0.98 | 0.33 |
| IL18 | 0.16 | 1.18 | 0.19 | 0.87 | 0.38 |
| IL6 | 0.05 | 1.05 | 0.10 | 0.48 | 0.63 |
| CCL3 | 0.07 | 1.07 | 0.15 | 0.47 | 0.64 |
| CCL23 | 0.01 | 1.01 | 0.18 | 0.06 | 0.95 |

**(c) │z│ > 2.5**

|  | **logHR** | **HR** | **se(logHR)** | **z** | **p** |
| --- | --- | --- | --- | --- | --- |
| ANGPT2 | 0.46 | 1.58 | 0.19 | 2.48 | 0.01 |
| MCP3 | 0.50 | 1.65 | 0.21 | 2.36 | 0.02 |

# Table S11. Comparison of the findings of Lindgaard et al. and of the present study

|  |  |  |  |  |  |  |  |  |  |  |  |
| --- | --- | --- | --- | --- | --- | --- | --- | --- | --- | --- | --- |
|  | **Lindgaard et al.** | | | | |  | **Present study** | | | | |
| **Protein** | **Index 1** | **Index 2** | **Median NPX in PDAC cases** | **Median NPX in healthy controls** | **Difference (cases - controls)** |  | **Associated with PC in first year** | **Associated with PC in first year (multivariable)** | **Median NPX in PC cases in 1st year** | **Median NPX in subcohort** | **Difference (cases - controls)** |
| ADGRG1 |  |  |  |  |  |  | X |  | 1.22 | 1.15 | 0.07 |
| ANGPT2 |  |  |  |  |  |  | X | X | 4.03 | 3.79 | 0.23 |
| ARG1 |  |  |  |  |  |  |  | X | 2.46 | 2.49 | -0.03 |
| CAIX |  |  |  |  |  |  | X |  | 3.62 | 3.32 | 0.30 |
| CASP-8 |  | X | 0.24 | -1.37 | 1.61 |  |  |  | 3.51 | 3.47 | 0.03 |
| CCL20 |  | X | 0.15 | -0.99 | 1.14 |  |  |  | 6.41 | 6.17 | 0.23 |
| CCL23 |  | X | 0.15 | -0.64 | 0.79 |  | X | X | 9.62 | 9.33 | 0.30 |
| CCL3 |  | X | 0.13 | -0.86 | 0.99 |  | X |  | 4.84 | 4.53 | 0.31 |
| CD27 |  |  |  |  |  |  | X | X | 7.88 | 7.51 | 0.37 |
| CD28 |  |  |  |  |  |  |  | X | 0.88 | 0.86 | 0.03 |
| CD4 | X | X | 0.1 | -0.61 | 0.71 |  | X | X | 0.15 | 0.09 | 0.06 |
| CD40 |  |  |  |  |  |  | X | X | 9.98 | 9.85 | 0.13 |
| CD40-L |  | X | 0.19 | 0.56 | -0.37 |  |  |  | 3.29 | 3.31 | -0.02 |
| CD5 |  |  |  |  |  |  | X |  | 4.34 | 4.32 | 0.02 |
| CD70 |  |  |  |  |  |  | X | X | 2.89 | 2.71 | 0.18 |
| CD83 |  |  |  |  |  |  | X | X | 1.86 | 1.70 | 0.16 |
| CD8A |  |  |  |  |  |  | X |  | 9.03 | 8.66 | 0.37 |
| CRTAM | X | X | 0.13 | -0.6 | 0.73 |  | X | X | 4.33 | 4.04 | 0.29 |
| CSF-1 |  | X | 0.3 | -1.04 | 1.34 |  |  | X | 7.10 | 6.99 | 0.10 |
| CX3CL1 |  |  |  |  |  |  | X |  | 5.60 | 5.40 | 0.20 |
| CXCL1 |  | X | 0.26 | 0.84 | -0.58 |  |  |  | 7.75 | 7.52 | 0.23 |
| CXCL10 |  |  |  |  |  |  |  | X | 7.59 | 7.40 | 0.19 |
| CXCL9 |  |  |  |  |  |  | X |  | 7.30 | 6.83 | 0.48 |
| FASLG | X | X | -0.23 | 0.86 | -1.09 |  |  |  | 5.90 | 5.94 | -0.04 |
| FGF2 |  |  |  |  |  |  |  | X | 0.48 | 0.48 | 0.00 |
|  | **Lindgaard et al.** | | | | |  | **Present study** | | | | |
| **Protein** | **Index 1** | **Index 2** | **Median NPX in PDAC cases** | **Median NPX in healthy controls** | **Difference (cases - controls)** |  | **Associated with PC in first year** | **Associated with PC in first year (multivariable)** | **Median NPX in PC cases in 1st year** | **Median NPX in subcohort** | **Difference (cases - controls)** |
| Gal-1 |  |  |  |  |  |  |  | X | 5.78 | 5.64 | 0.15 |
| GZMA |  |  |  |  |  |  | X | X | 5.01 | 4.95 | 0.07 |
| HGF |  |  |  |  |  |  | X | X | 7.36 | 7.01 | 0.35 |
| IFN-beta |  |  |  |  |  |  |  | X | 0.44 | 0.44 | 0.00 |
| IL1alpha |  | X | -0.13 | -0.16 | 0.03 |  |  | X | 0.42 | 0.42 | 0.00 |
| IL10 | X | X | 0.06 | -0.76 | 0.82 |  |  |  | 2.00 | 1.91 | 0.09 |
| IL12 |  | X | 0.09 | -0.3 | 0.39 |  |  |  | 5.50 | 5.49 | 0.01 |
| IL13 |  |  |  |  |  |  |  | X | 1.43 | 1.43 | 0.00 |
| IL18 |  |  |  |  |  |  | X |  | 8.23 | 8.04 | 0.19 |
| IL2 |  |  |  |  |  |  | X |  | 0.92 | 0.92 | 0.00 |
| IL33 |  | X | 0.08 | -0.56 | 0.64 |  |  | X | 0.93 | 0.93 | 0.00 |
| IL4 |  |  |  |  |  |  |  | X | 0.99 | 0.99 | 0.00 |
| IL5 |  | X | -0.28 | -0.29 | 0.01 |  |  |  | 2.20 | 2.20 | 0.00 |
| IL6 |  |  |  |  |  |  | X | X | 2.93 | 2.47 | 0.46 |
| IL8 | X | X | 0.14 | -1.09 | 1.23 |  | X | X | 4.81 | 4.23 | 0.58 |
| LAMP3 |  | X | -0.08 | -0.34 | 0.26 |  | X | X | 4.10 | 3.65 | 0.45 |
| LAP-TGF-beta1 |  |  |  |  |  |  |  | X | 0.43 | 0.36 | 0.07 |
| MCP-2 |  |  |  |  |  |  |  | X | 6.59 | 6.56 | 0.03 |
| MCP-3 | X | X | 0.19 | -0.96 | 1.15 |  |  | X | 1.69 | 1.37 | 0.32 |
| MIC-A/B |  | X | 0.35 | -0.08 | 0.43 |  |  |  | 3.34 | 3.37 | -0.03 |
| MMP-12 |  |  |  |  |  |  | X | X | 7.45 | 6.72 | 0.72 |
| MMP-7 | X | X | 0.44 | -1.23 | 1.67 |  | X | X | 9.79 | 8.83 | 0.96 |
| NCR1 |  |  |  |  |  |  | X | X | 3.49 | 3.26 | 0.23 |
| PD-L2 |  | X | 0.16 | -0.68 | 0.84 |  |  | X | 1.70 | 1.60 | 0.10 |
| PDCD1 |  |  |  |  |  |  | X | X | 2.96 | 2.67 | 0.29 |
| PGF |  |  |  |  |  |  | X |  | 7.87 | 7.66 | 0.21 |
| PTN |  |  |  |  |  |  | X |  | 1.27 | 0.40 | 0.87 |
|  | **Lindgaard et al.** | | | | |  | **Present study** | | | | |
| **Protein** | **Index 1** | **Index 2** | **Median NPX in PDAC cases** | **Median NPX in healthy controls** | **Difference (cases - controls)** |  | **Associated with PC in first year** | **Associated with PC in first year (multivariable)** | **Median NPX in PC cases in 1st year** | **Median NPX in subcohort** | **Difference (cases - controls)** |
| TNFRSF12A |  |  |  |  |  |  | X | X | 5.40 | 5.20 | 0.20 |
| TNFRSF21 |  |  |  |  |  |  | X |  | 7.36 | 7.21 | 0.15 |
| TNFRSF4 |  |  |  |  |  |  | X |  | 2.85 | 2.59 | 0.26 |
| TNFRSF9 |  |  |  |  |  |  | X | X | 5.03 | 4.61 | 0.42 |
| TRAIL | X | X | -0.18 | 0.53 | -0.71 |  |  | X | 7.25 | 7.30 | -0.05 |
| VEGFC | X | X | -0.05 | 0.37 | -0.42 |  |  |  | 0.06 | 0.04 | 0.02 |
|  |  |  |  |  |  |  |  |  |  |  |  |

PDAC: pancreatic ductal adenocarcinoma; PC: pancreatic cancer; NPX: normalised protein expression

# Figure S1. Histograms of proteins

The blue vertical line denotes the lower limit of detection (LOD).

**
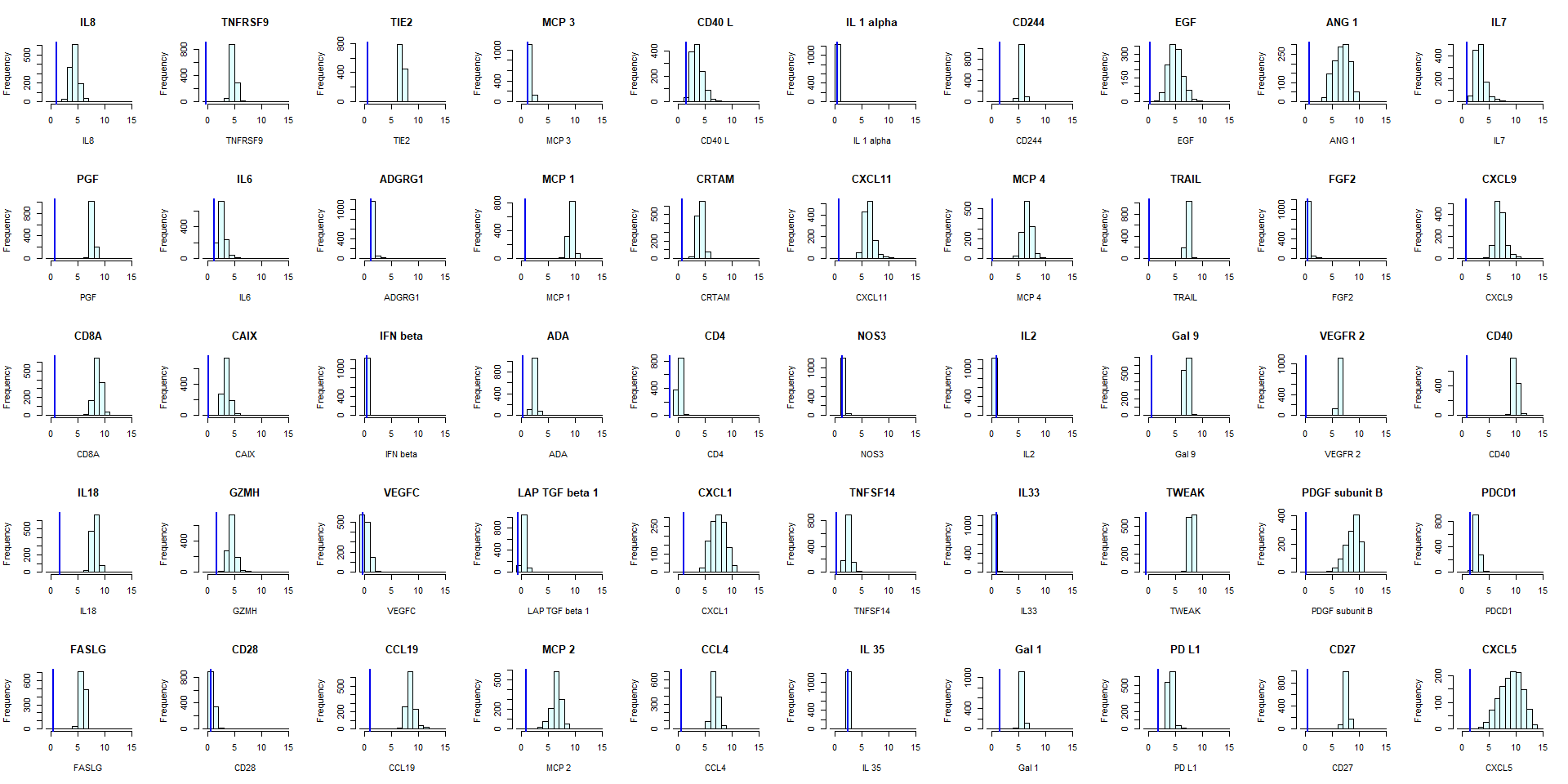
**

**
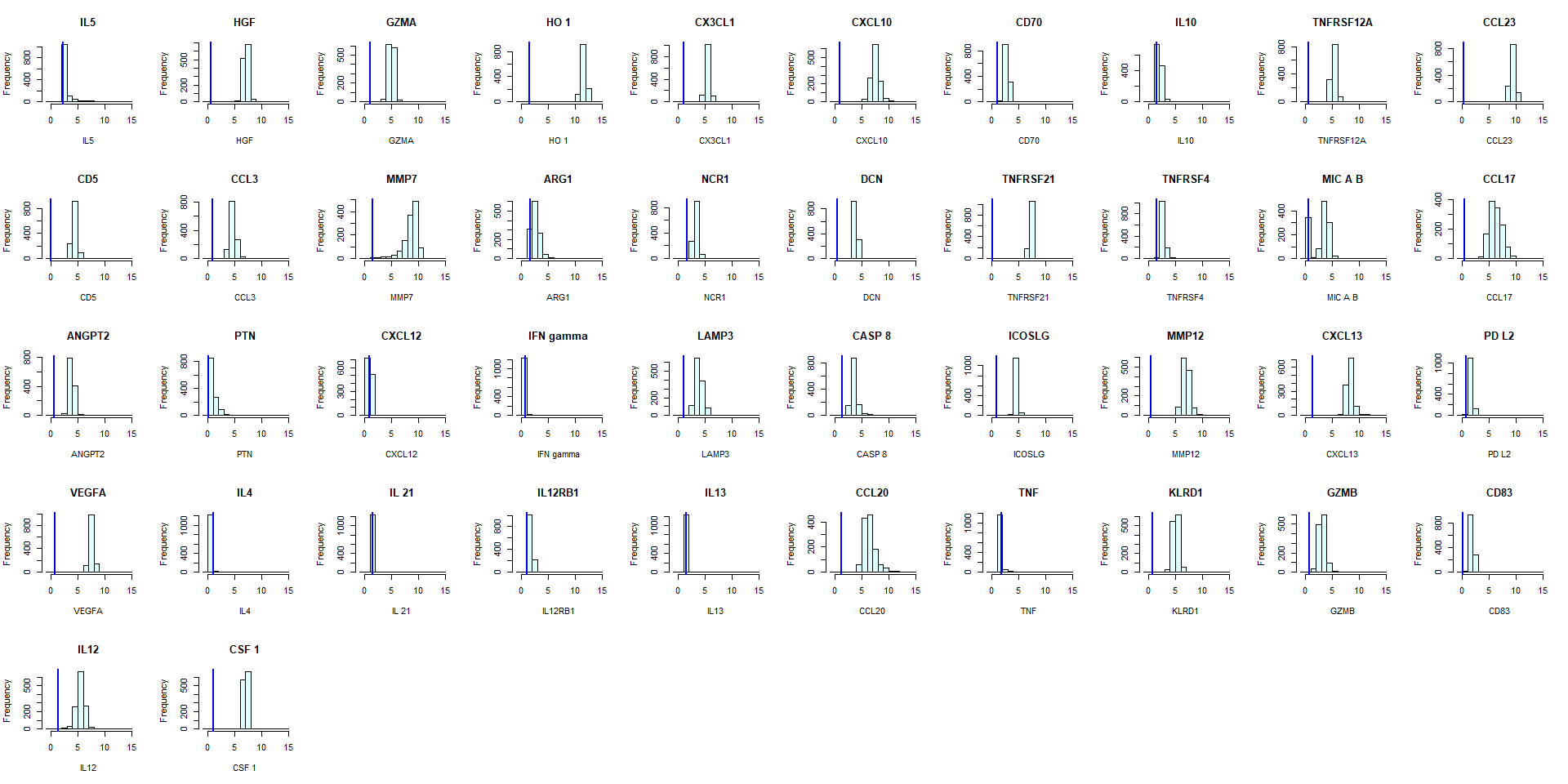
**

# Figure S2. Correlations between proteins in the subcohort

**
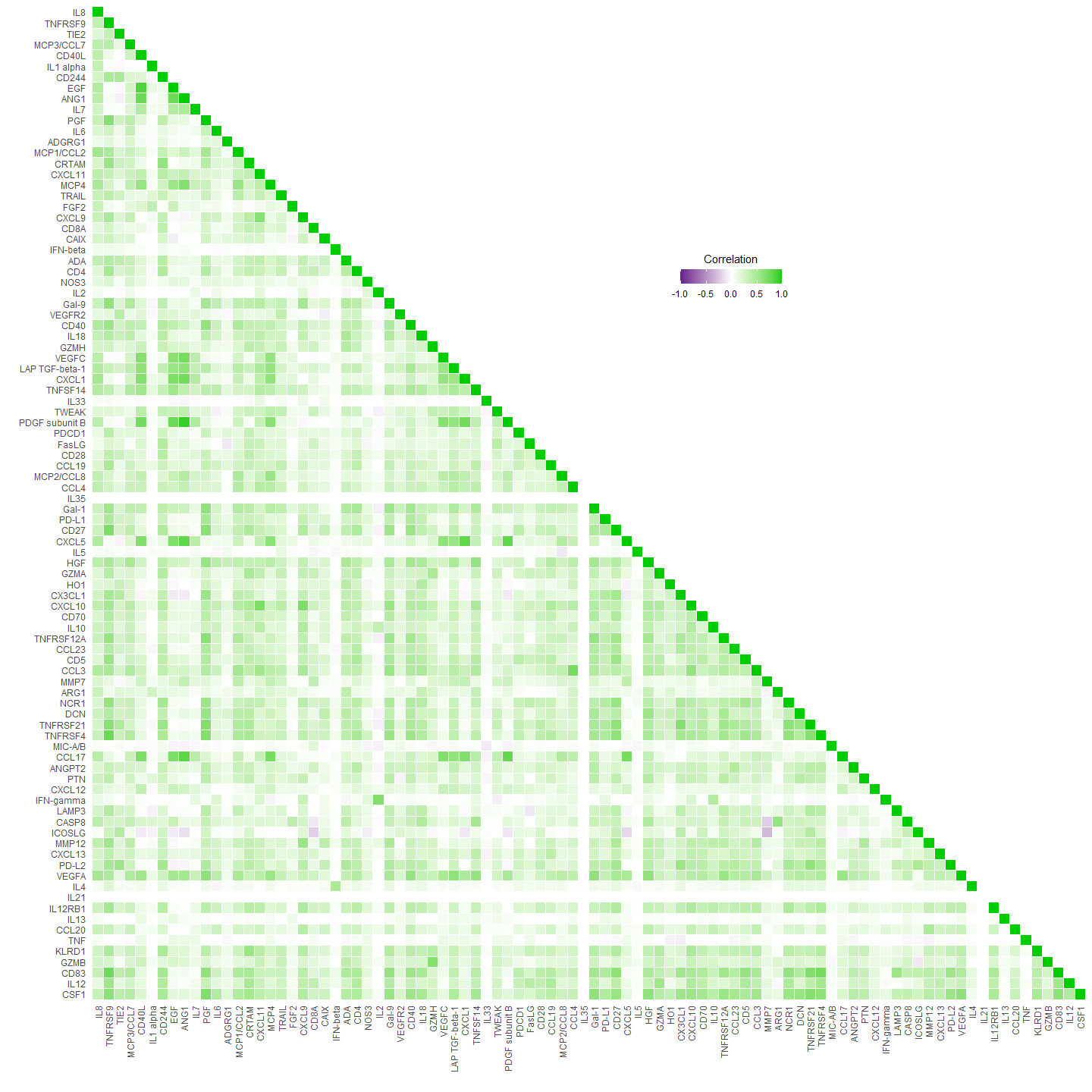
**

# Figure S3. Hazard ratios (95% CI) for selected proteins (for a SD higher NPX), adjusted for age, sex and stratified by region.

Time in study was used as the time scale. MCP3: Monocyte chemotactic protein 3; ANGPT2: Angiopoietin-2; IL18: Interleukin-18; LAMP3: Lysosome-associated membrane glycoprotein 3; IL6: Interleukin-6; CCL3: C-C motif chemokine 3; IL2: Interleukin-2; CD8A: T-cell surface glycoprotein CD8 alpha chain; HO1: Heme oxygenase 1; CD4: T-cell surface glycoprotein; HGF: Hepatocyte growth factor; CD40: CD40L receptor; IL5: Interleukin-5; TNFRSF9: Tumor necrosis factor receptor superfamily member 9; GZMA: Granzyme A; ADGRG1: Adhesion G-protein couples receptor G1; TIE2: Angiopoietin-1 receptor.

**
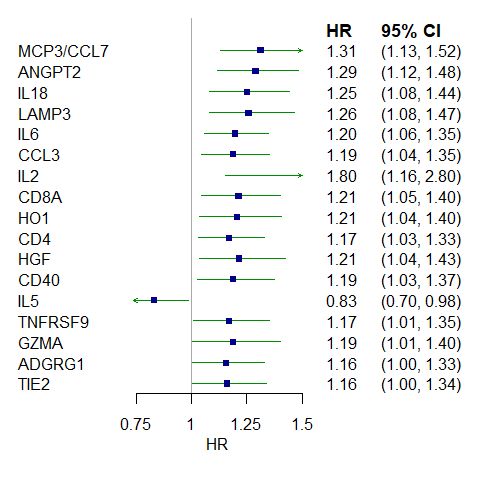
**

# Figure S4. Hazard ratios (95% CI) for NPX greater than versus less than or equal to the limit of detection, for proteins for which at least 500 individuals had values below the limit of detection.

Models were adjusted for age, age^2^, sex, smoking status, alcohol drinking, education, diabetes, time since last meal and stratified by region. Time in study was used as the time scale. TNF: Tumor necrosis factor; IL2: Interleukin-2; IL4: Interleukin-4; IL5: Interleukin-5; IL13: Interleukin-13; IFN-beta: Interferon beta; IL33: Interleukin-33; NOS3: Nitric oxide synthase, endothelial; FGF2: Fibroblast growth factor 2; CXCL12: Stromal cell-derived factor 1; IL1 alpha: Interleukin-1 alpha; ADGRG1: Adhesion G-protein coupled receptor G1; IFN-gamma: Interferon gamma.


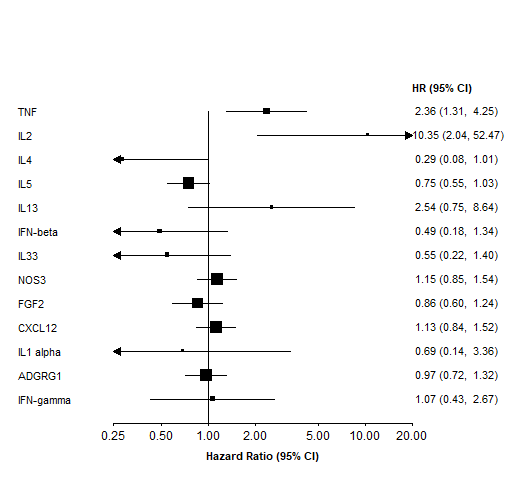


# Figure S5. Adjusted hazard ratios for pancreatic cancer associated with per SD higher NPX by category of proteins.

Models were adjusted for age, age^2^, sex, smoking status, alcohol drinking, education, diabetes, time since last meal and stratified by region.
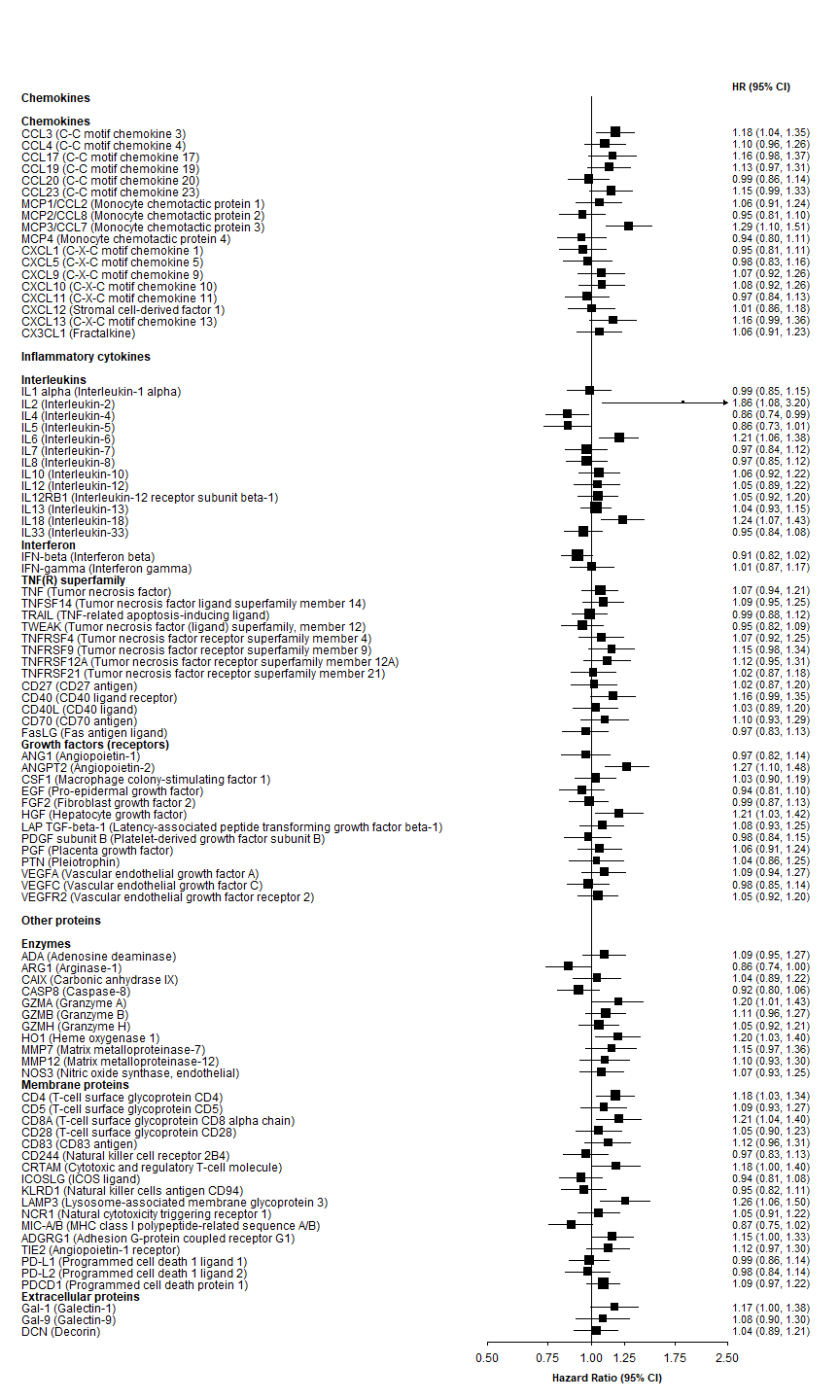


# Figure S6. Hazard ratios (95% CI) for all proteins with at least 500 individuals with values over the limit of detection, by NPX split at quartiles.

Proteins were split at tertiles when quartiles were not unique. Models were adjusted for age, age^2^, sex, smoking status, alcohol drinking, education, diabetes, time since last meal and stratified by region. Time in study was used as the time scale. **
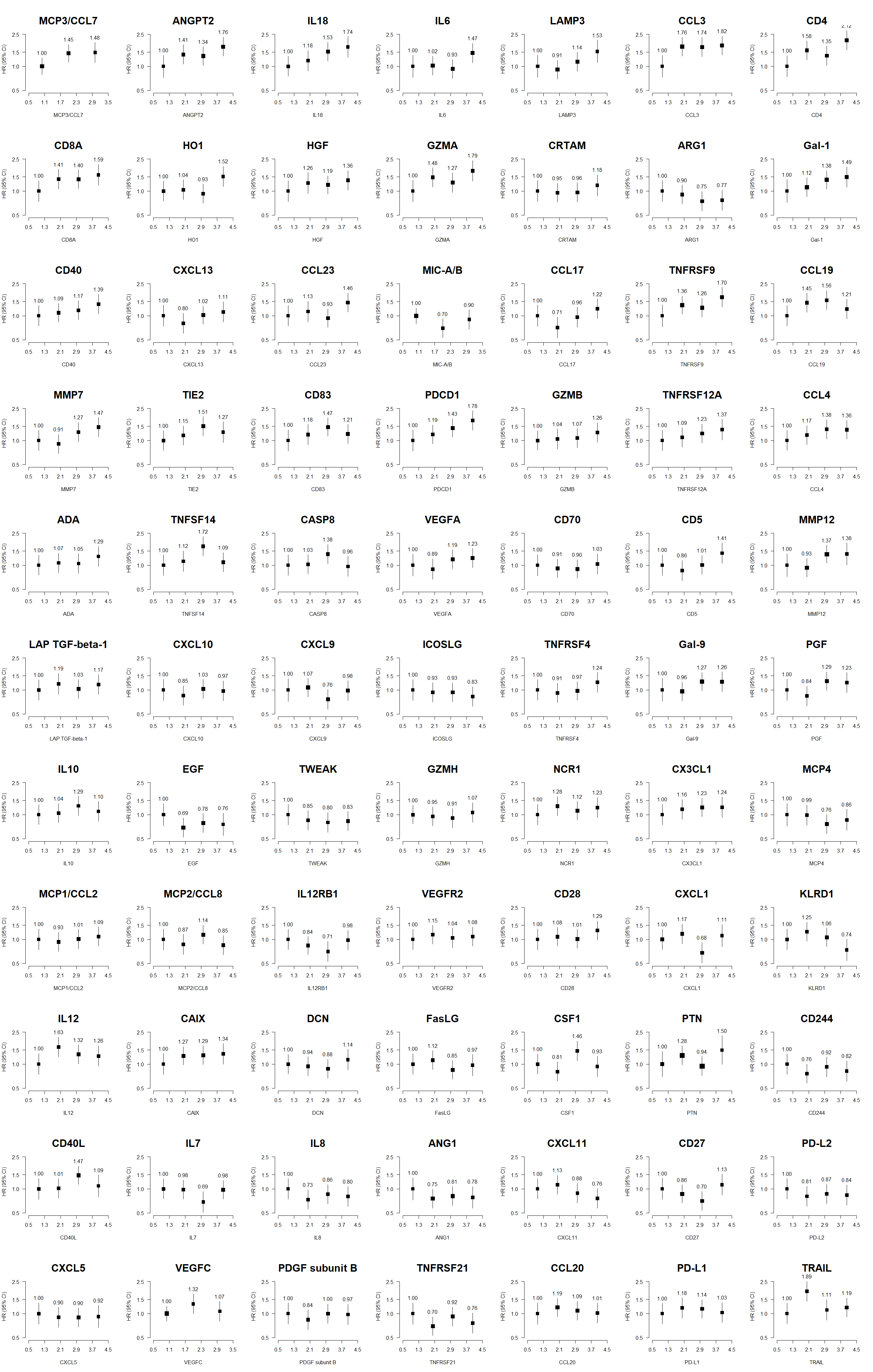
**

# Figure S7. Spline fits for NPX for all proteins with at least 500 individuals with values over the limit of detection. Models were adjusted for age, age^2^, sex, smoking status, alcohol drinking, education, diabetes, time since last meal and stratified by region. Time in study was used as the time scale. x axis values are NPX divided by its standard deviation.

**
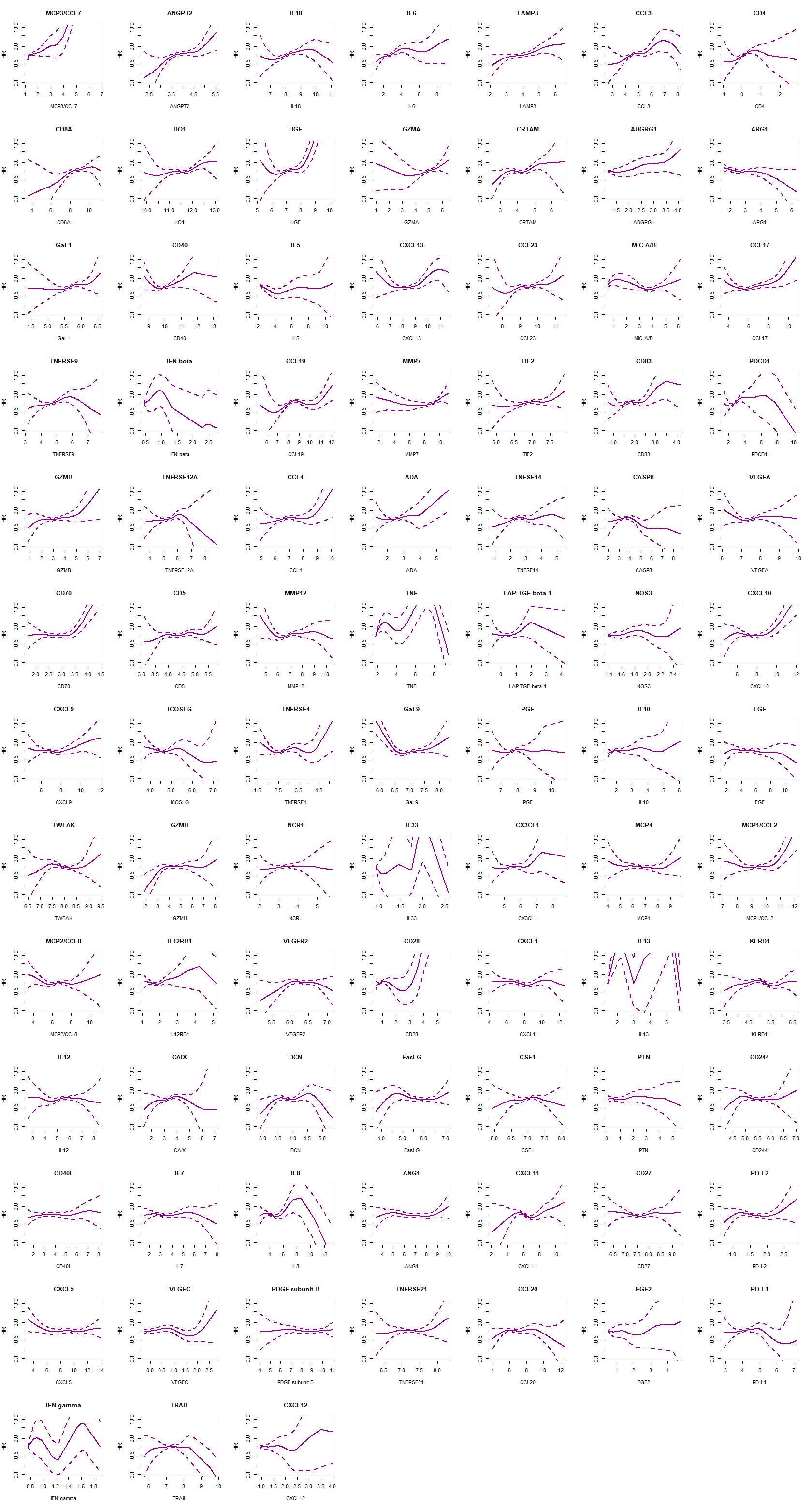
**

# Figure S8. Scaled Schoenfeld residuals for selected proteins for which there is evidence of time dependence of hazard ratios.

Proteins with χ^2^ test p-value ≤ 0.05, ordered in increasing p-value.

**
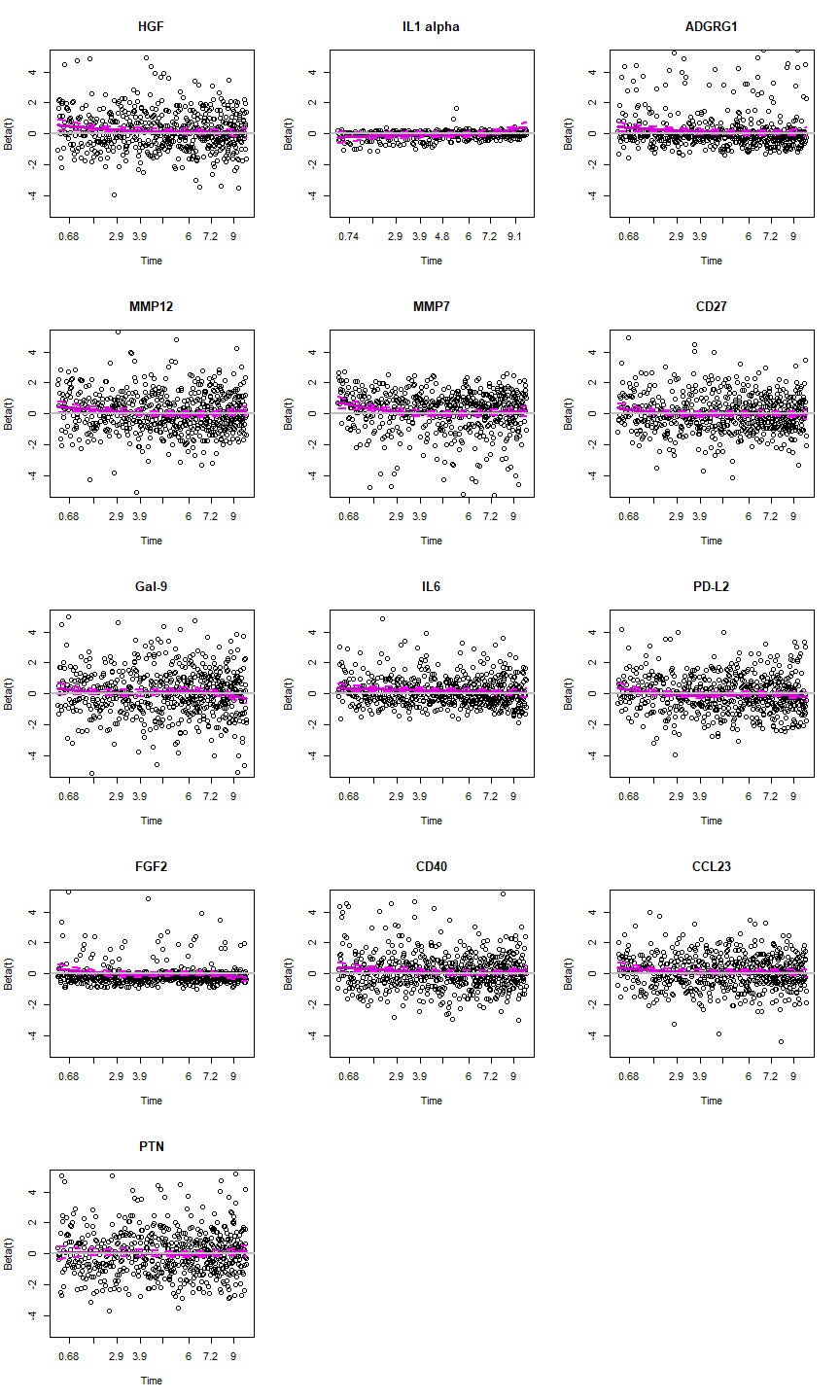
**

# Figure S9. Scaled Schoenfeld residuals for selected proteins for which there is no evidence of time dependence of hazard ratios.

Proteins with χ^2^ test p-value > 0.05, ordered in decreasing p-value. **
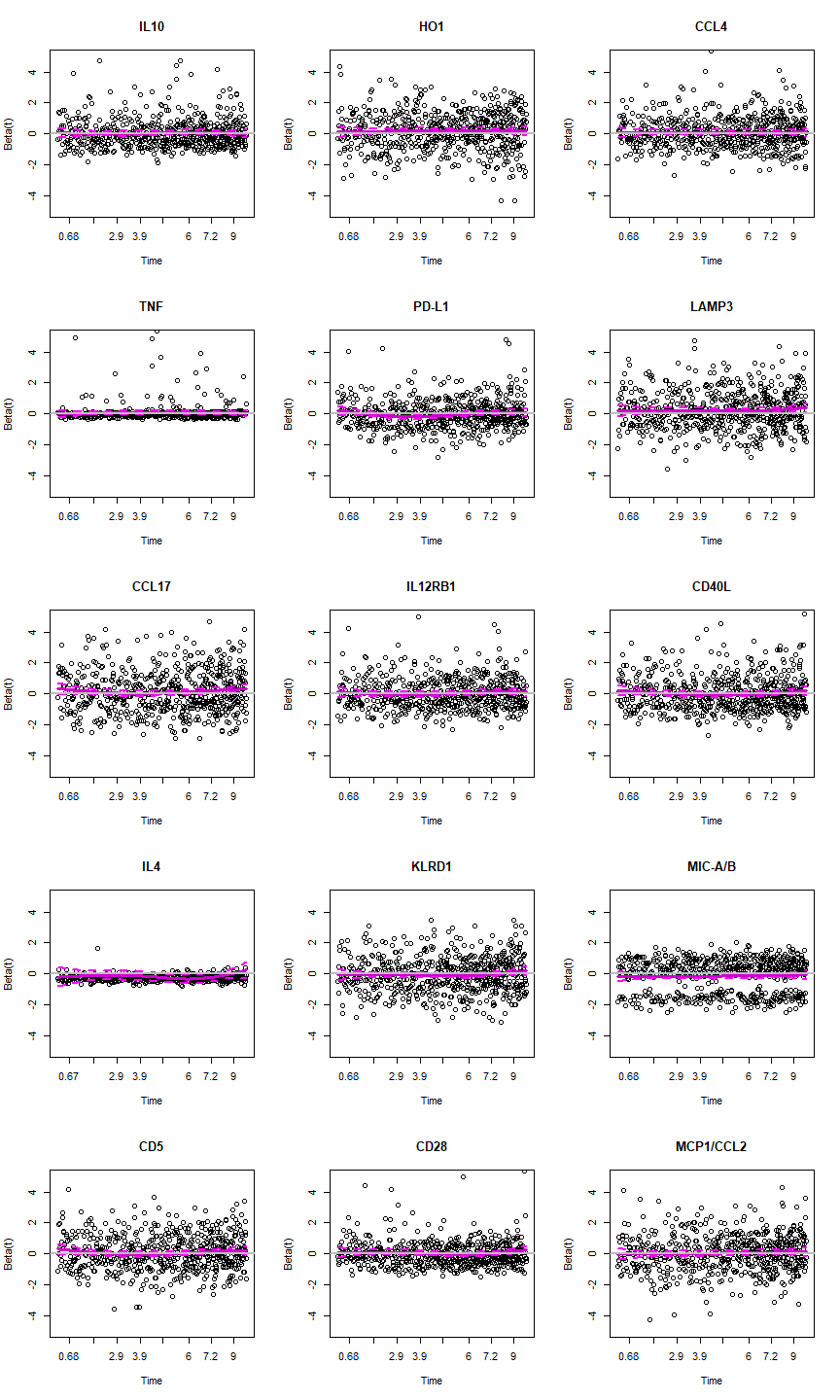
**

**
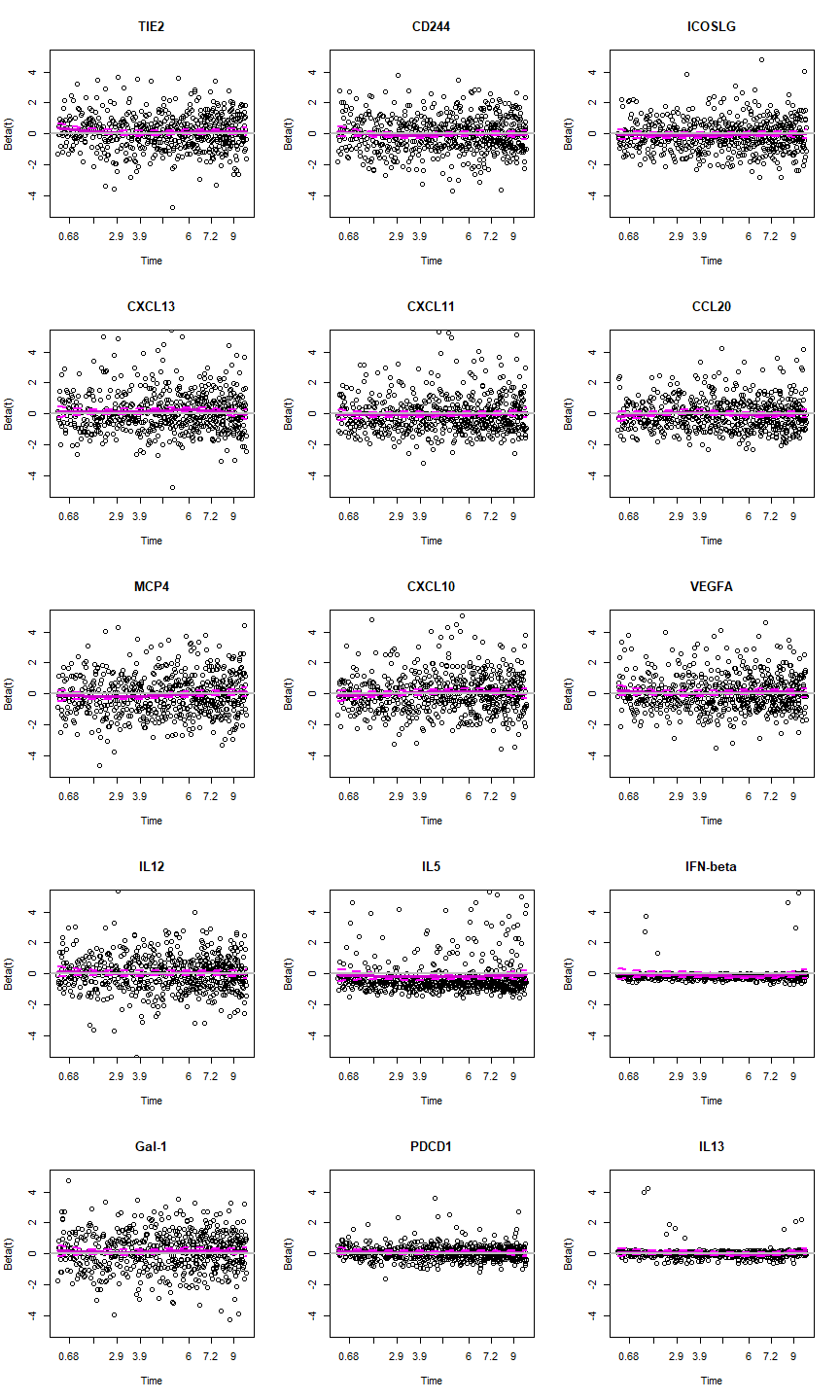
**

**
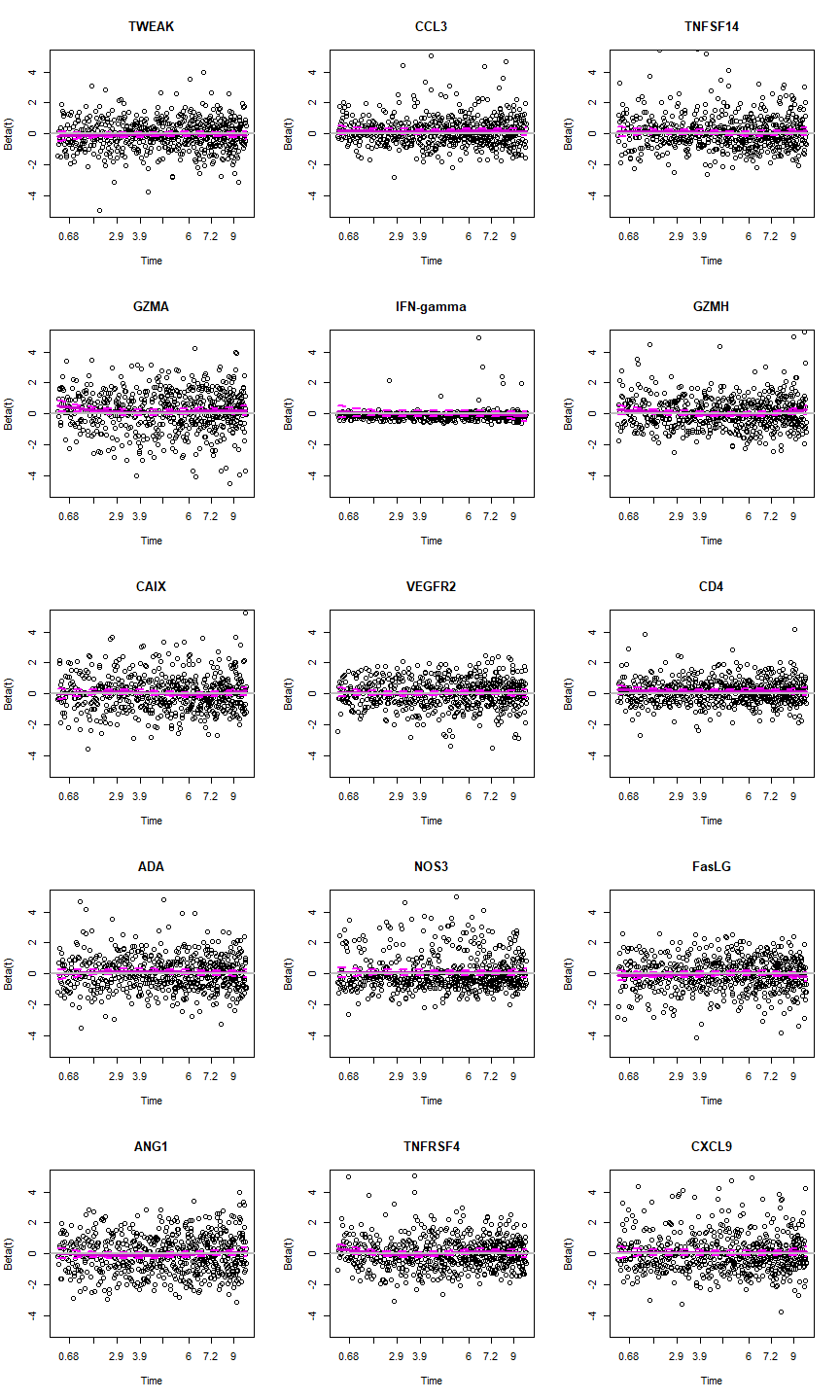
**

**
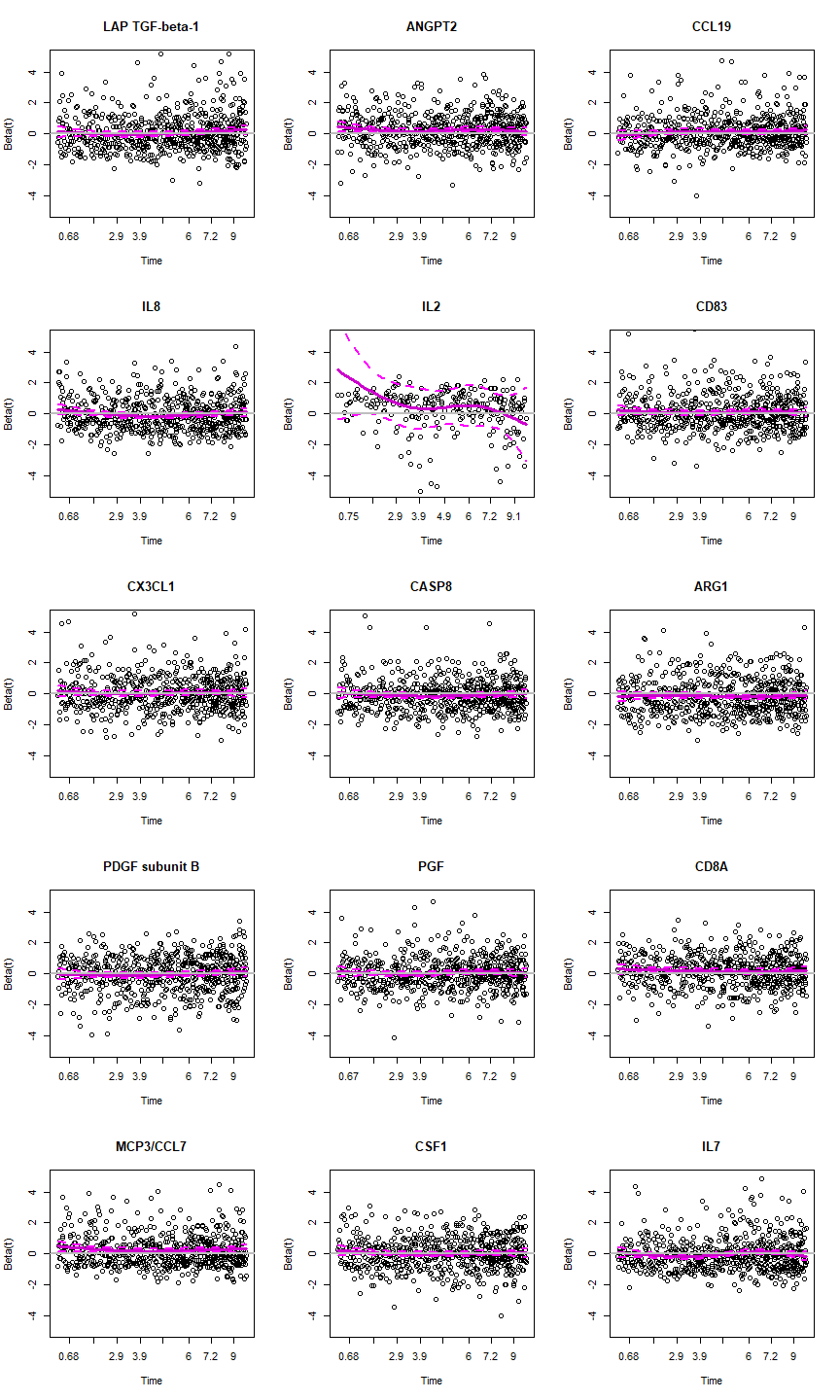
**

**
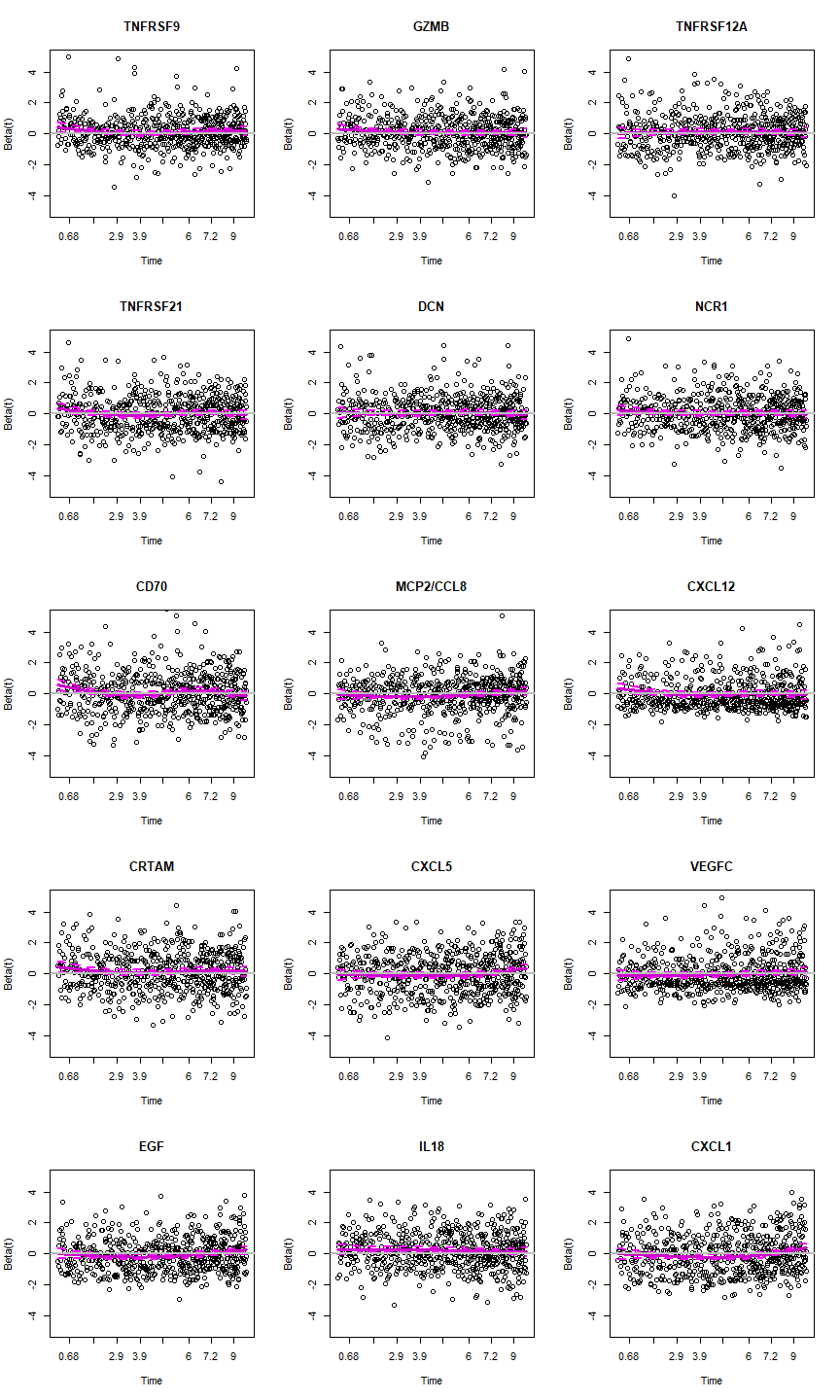
**

**
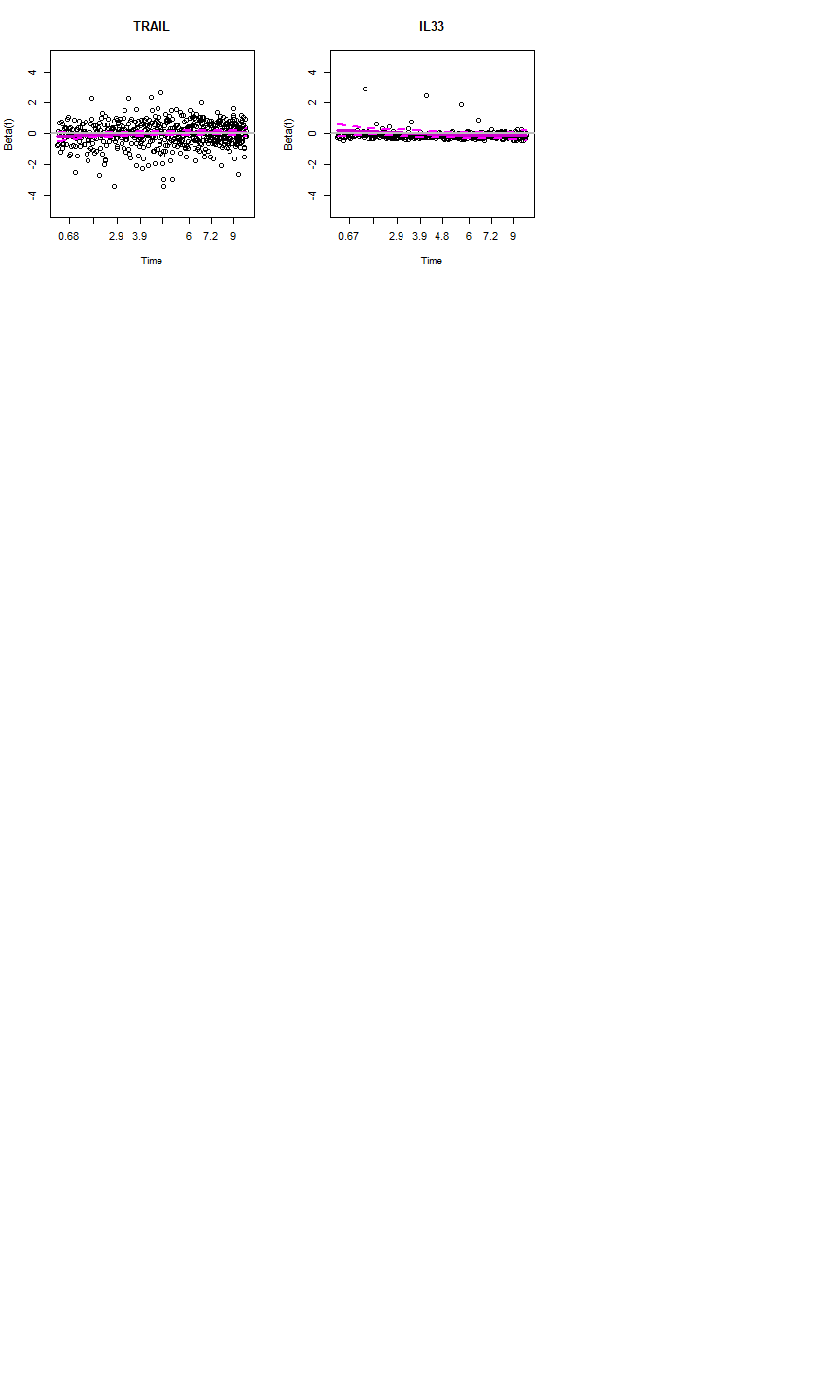
**

# Figure S10. Transformed p-values against their expected values for associations within the first year of follow-up.

Models were adjusted for age, age^2^, sex, smoking status, alcohol drinking, education, time since last meal and stratified by region. Time in study was used as the time scale. The green dashed line is a line with slope 1.

**
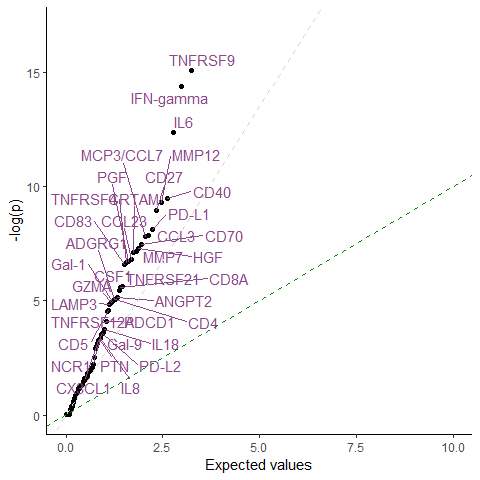
**

# Figure S11. Hazard ratios (95% CI) per SD higher NPX by time since study entry.

Models were adjusted for age, age^2^, sex, smoking status, alcohol drinking, education, time since last meal and stratified by region. Time in study was used as the time scale.

**
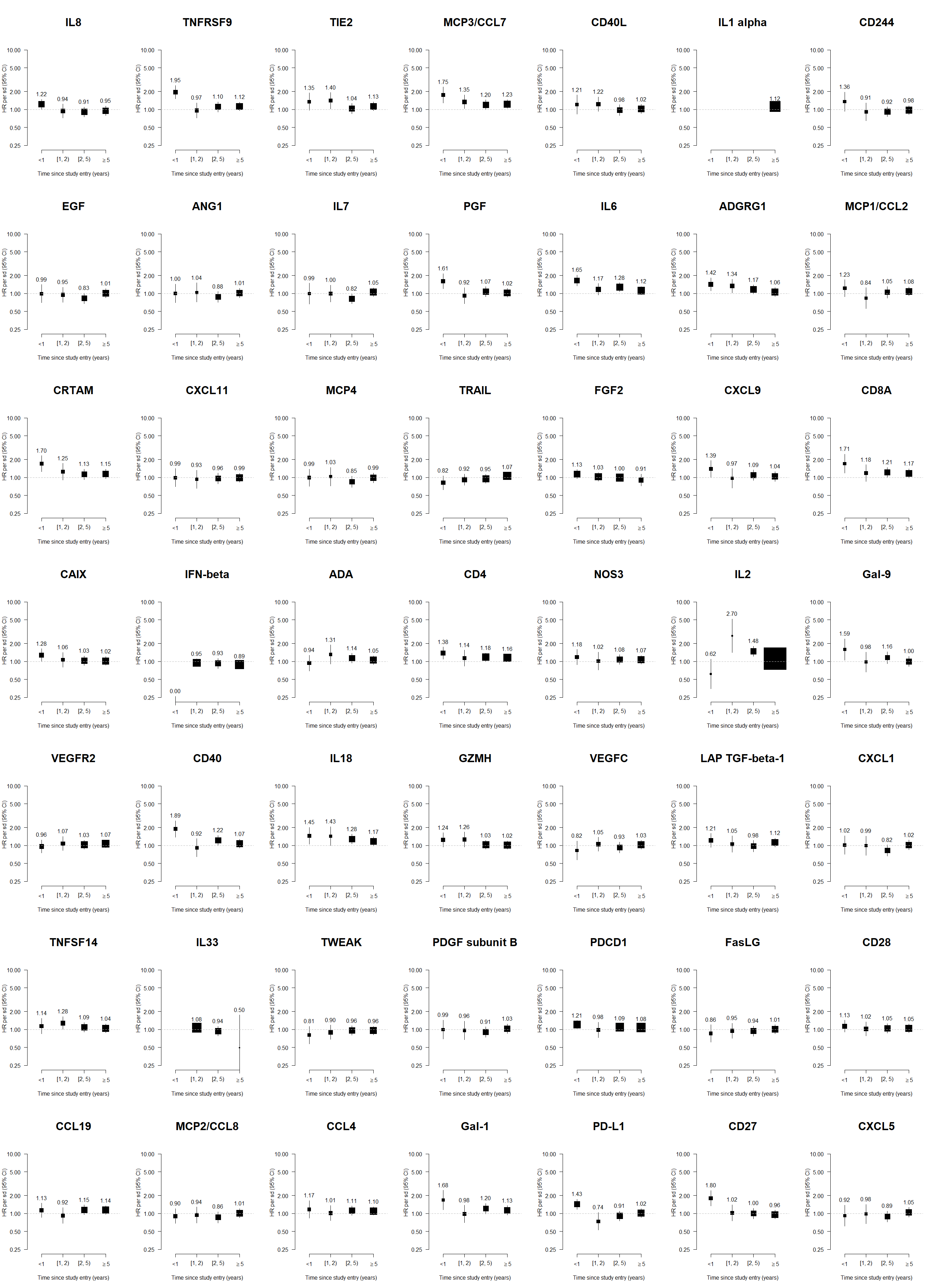
**

**
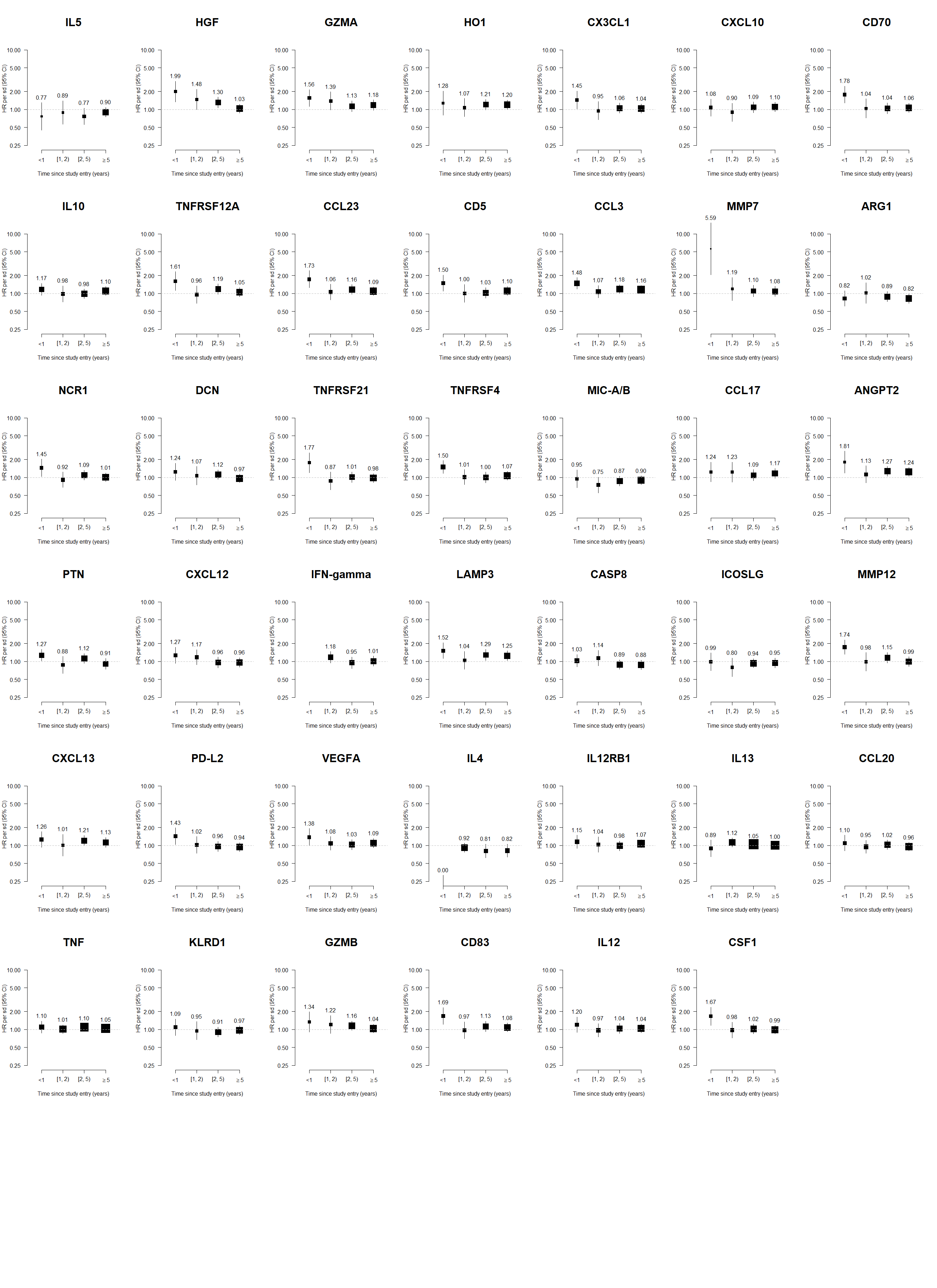
**

# Figure S12. Hazard ratios (95% CI) per SD higher NPX in individuals aged <60 and ≥60 years at sample collection.

Models were adjusted for age, age^2^, sex, smoking status, alcohol drinking, education, diabetes, time since last meal and stratified by region. Time in study was used as the time scale.

**
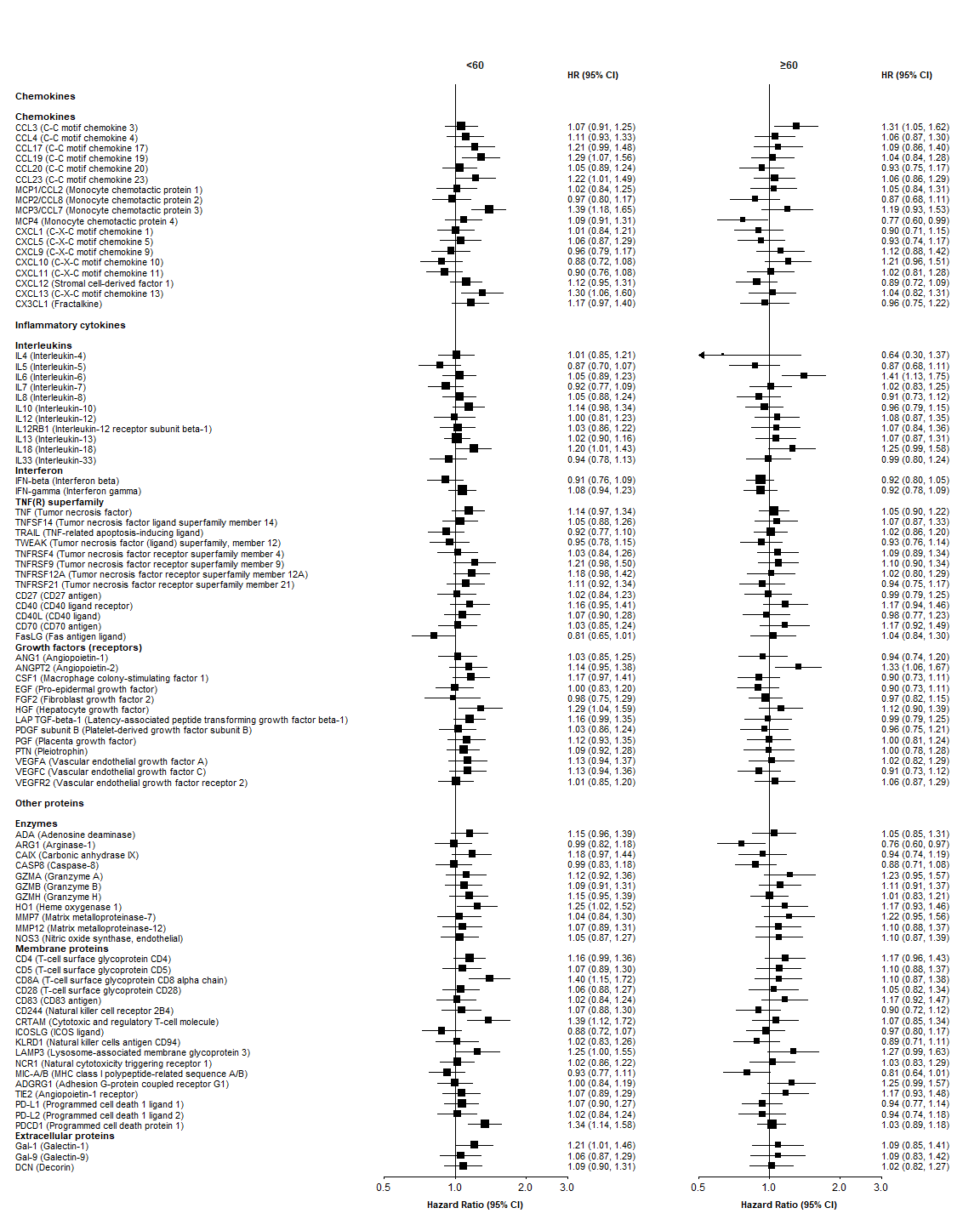
**

# Figure S13. Hazard ratios (95% CI) per SD higher NPX in males and females.

Models were adjusted for age, age^2^, smoking status, alcohol drinking, education, diabetes, time since last meal and stratified by region. Time in study was used as the time scale.

**
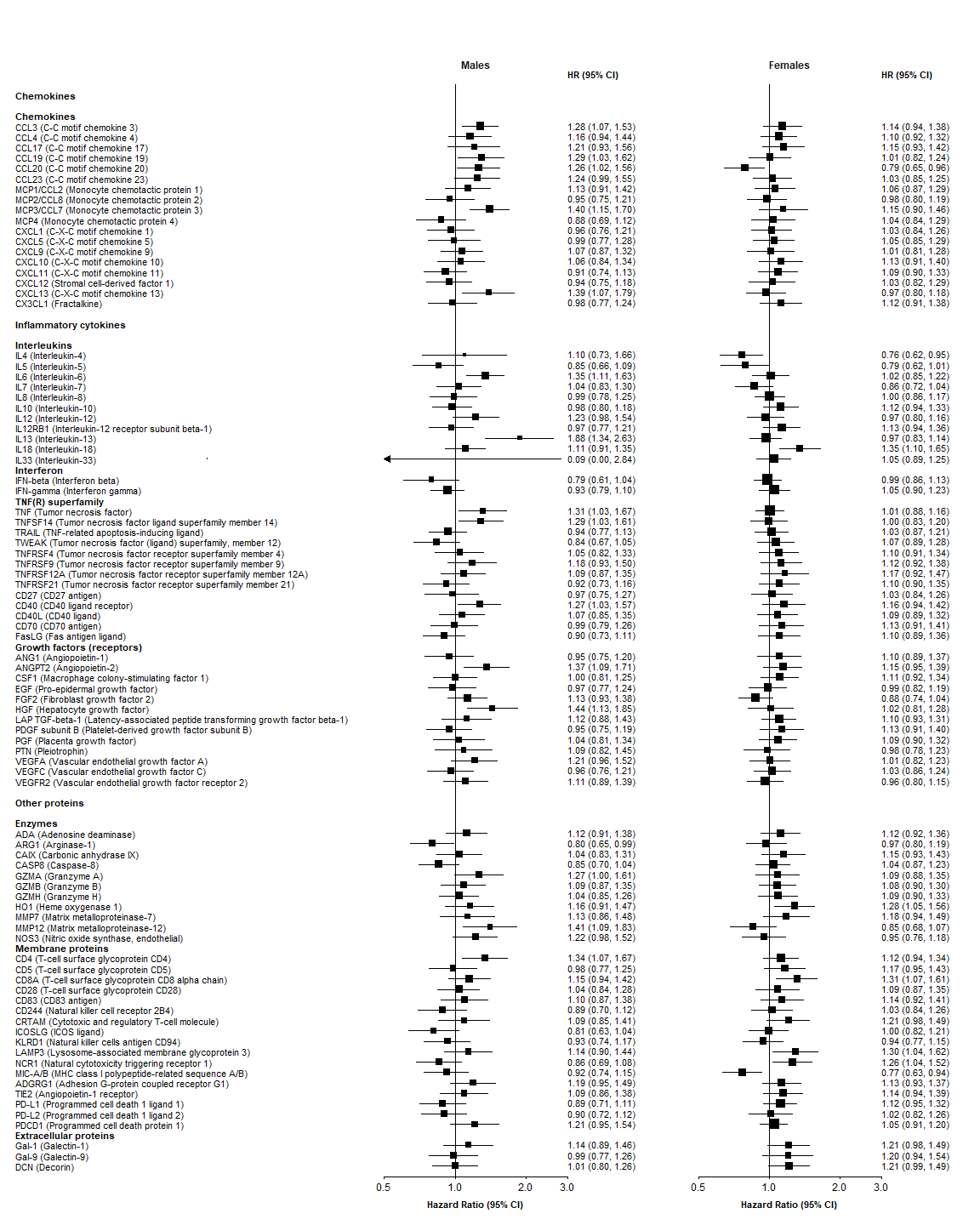
**

# Figure S14. Hazard ratios (95% CI) per SD higher NPX in individuals from rural and urban regions.

Models were adjusted for age, age^2^, sex, smoking status, alcohol drinking, education, diabetes, time since last meal and stratified by region. Time in study was used as the time scale.

**
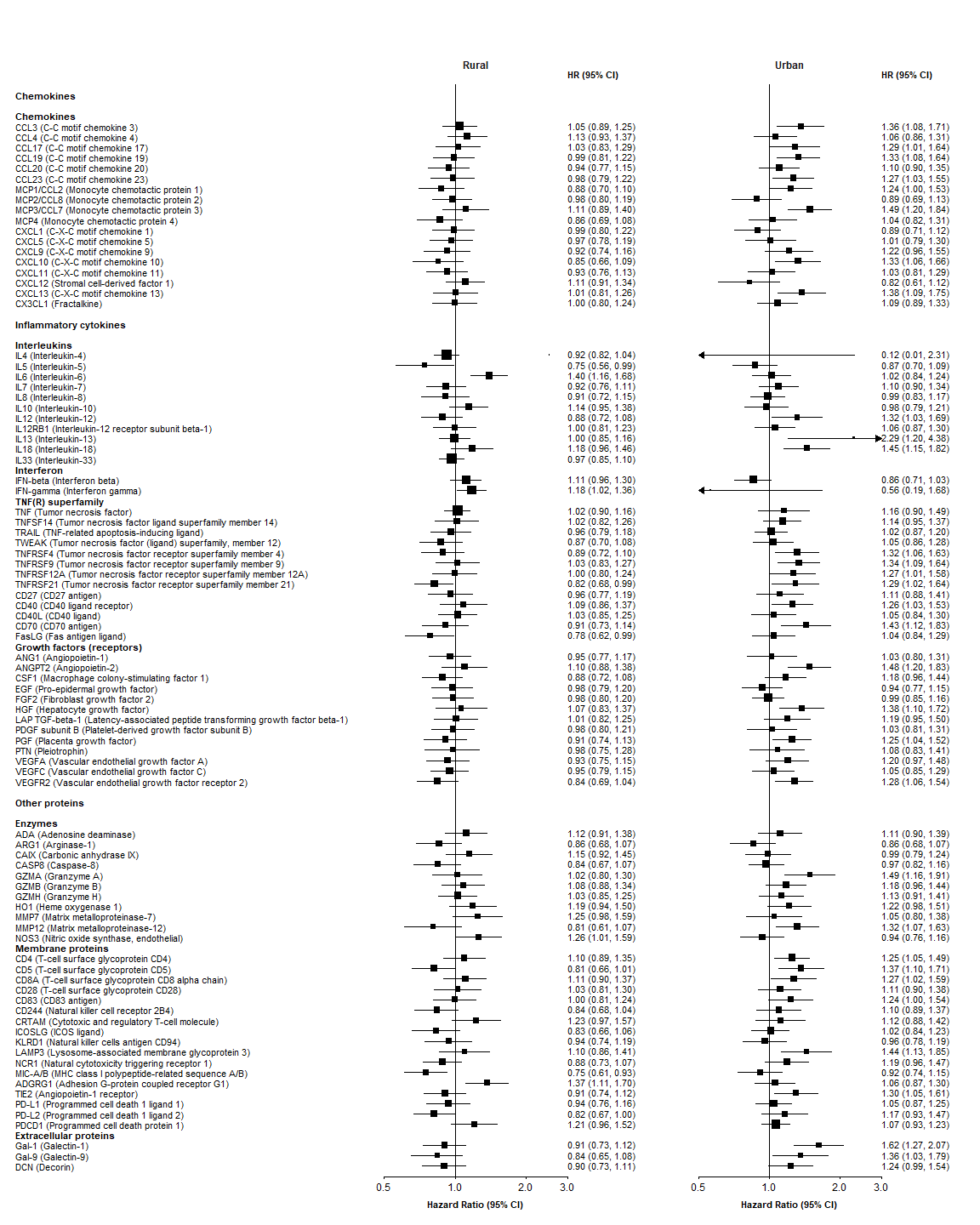
**

# Figure S15. Hazard ratios (95% CI) per SD higher NPX by diabetes status at sample collection.

Models were adjusted for age, age^2^, sex, smoking status, alcohol drinking, education, time since last meal and stratified by region. Time in study was used as the time scale.

**
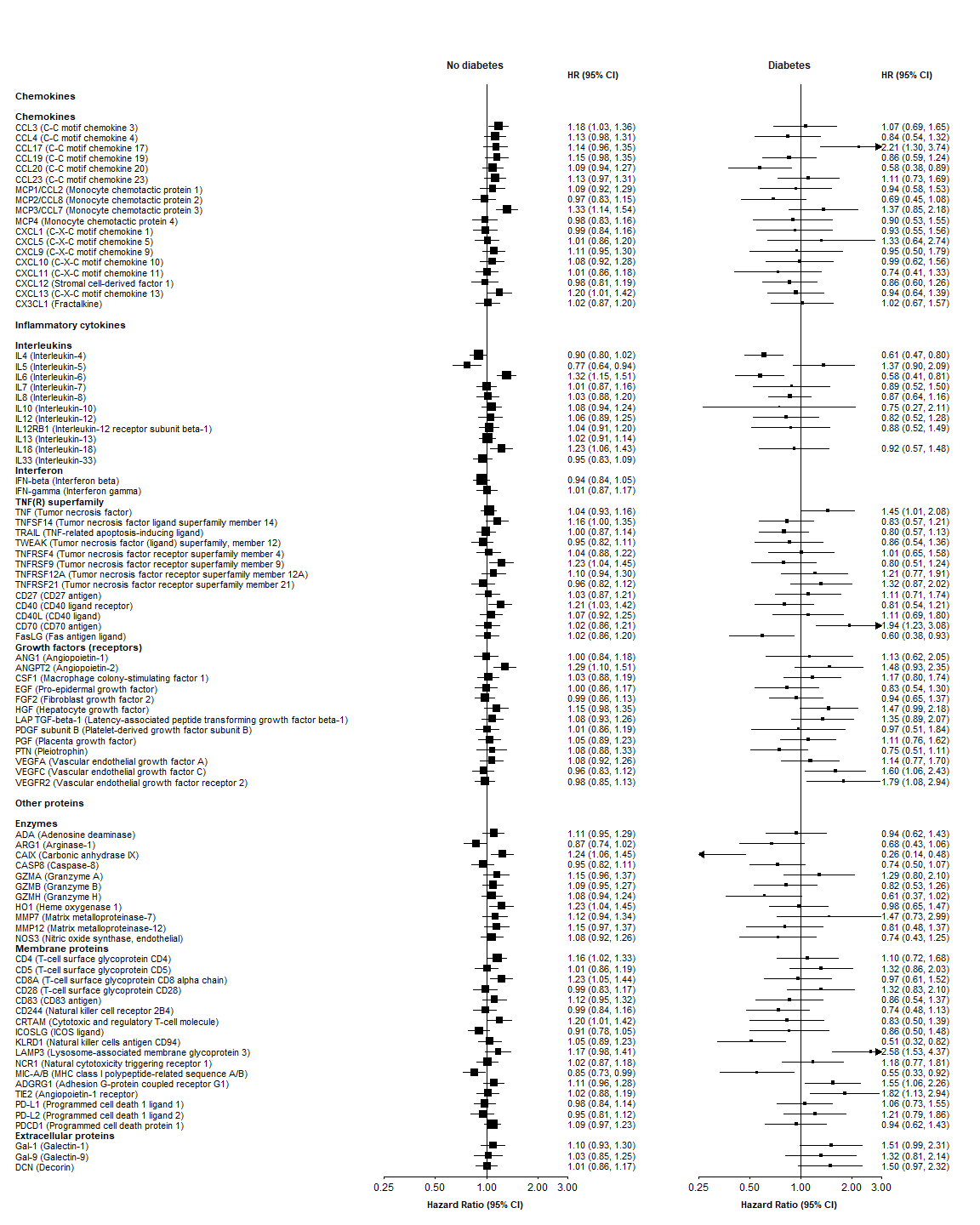
**

# Figure S16. Hazard ratios (95% CI) per SD higher NPX by smoking status at sample collection.

Models were adjusted for age, age^2^, sex, smoking status, alcohol drinking, education, time since last meal and stratified by region. Time in study was used as the time scale.

**
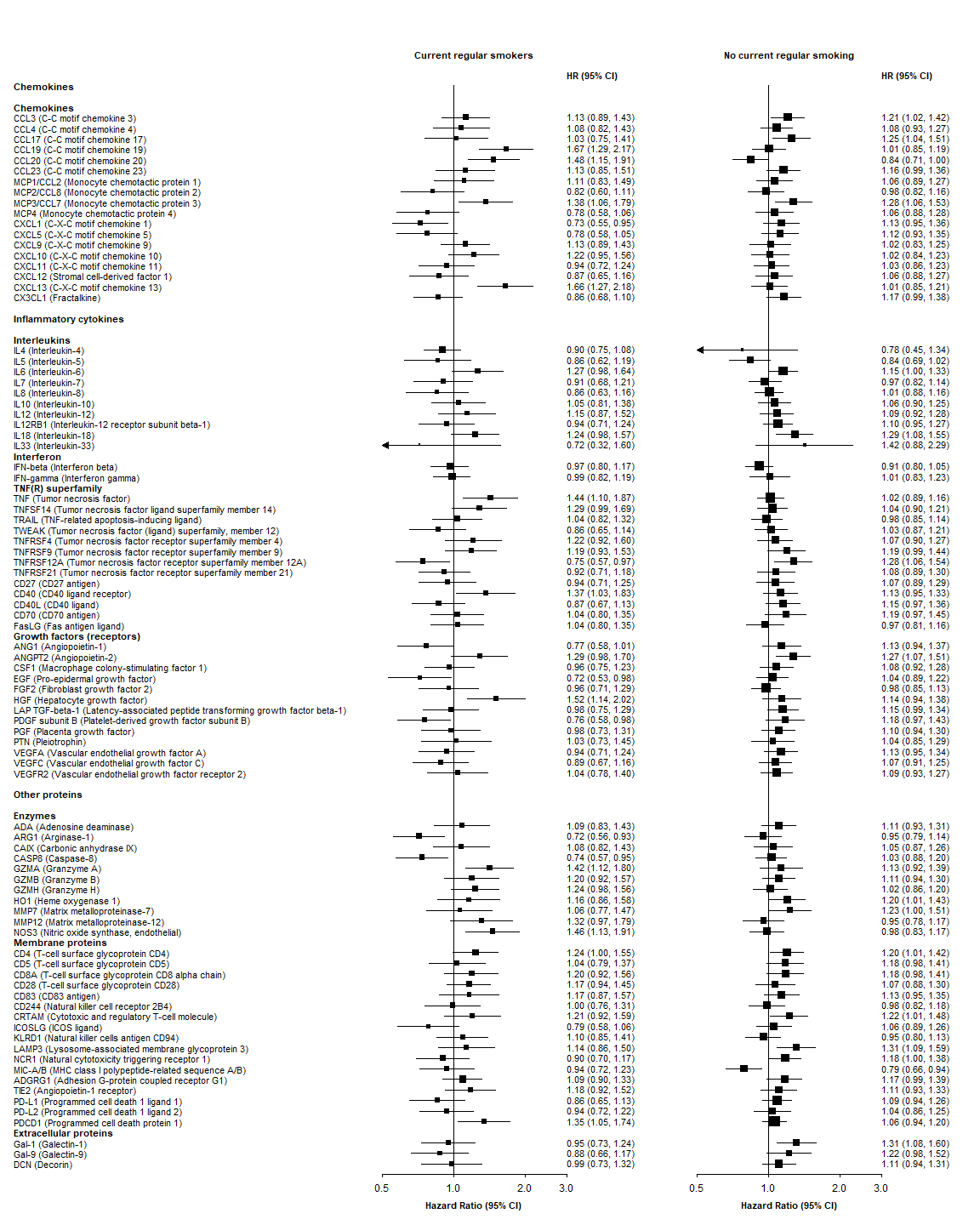
**

# Figure S17. Hazard ratios (95% CI) for selected proteins (for a SD higher NPX), with age as the time scale and entry at age at baseline.

Models were adjusted for age, age^2^, sex, smoking status, alcohol drinking, education, diabetes, time since last meal and stratified by region. IL18: Interleukin-18; MCP3: Monocyte chemotactic protein 3; ANGPT2: Angiopoietin-2; IL6: Interleukin-6; LAMP3: Lysosome-associated membrane glycoprotein 3; HO1: Heme oxygenase 1; MIC-A/B: MHC class I polypeptide-related sequence A/B; CD4: T-cell surface glycoprotein; CD8A: T-cell surface glycoprotein CD8 alpha chain; HGF: Hepatocyte growth factor; CRTAM: Cytotoxic and regulatory T-cell molecule; CXLC13: Stromal cell-derived factor 1.


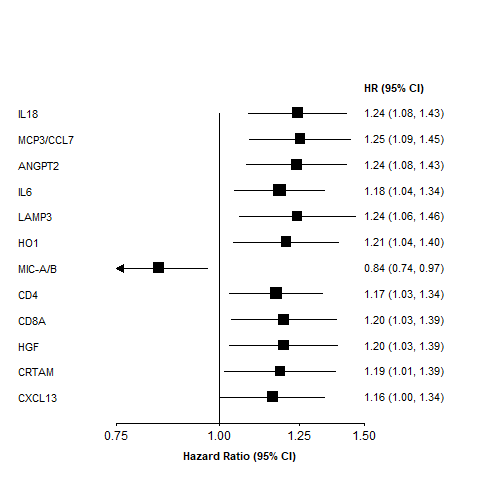

Supplement: supplementary [file EMS143804-supplement-supplementary.docx]
